# Tailoring electrolyte phase separation for high-rate solid-state lithium metal batteries

**DOI:** 10.1038/s41467-026-74094-w

**Published:** 2026-06-08

**Authors:** Shiyu Zhang, Jiantao Li, Benli Jiang, Guanyi Wang, Chengkun Zhang, Chengyu Wang, Xinchao Hu, Jie Shen, Ziyi Fang, Liang Lin, Guiyang Gao, Yuming Jin, Baisheng Sa, Laisen Wang, Jie Lin, Qingshui Xie, Dong-Liang Peng

**Affiliations:** 1 State Key Laboratory of Physical Chemistry of Solid Surface, College of Materials, Xiamen University, Xiamen, China; 2 Department of Chemical and Biological Engineering, Northwestern University, Evanston, IL USA; 3 Division of Materials Science and Engineering, Boston University, Boston, MA USA; 4 Department of Chemical and Paper Engineering, Western Michigan University, Kalamazoo, MI USA; 5 College of Materials Science and Engineering, Fuzhou University, Fuzhou, China

**Keywords:** Energy, Batteries, Batteries, Batteries

## Abstract

Solid polymer electrolytes are attractive for solid-state lithium metal batteries due to their flexibility and safety but suffer from low ionic conductivity and unstable interfaces. Conventional polymerization-induced phase separation strategies enhance ion transport yet rely on external components such as deep eutectic solvents or ionic liquids, increasing cost and complexity. Here, a LiTFSI-mediated in-situ polymerization strategy is developed to induce controllable phase separation in a poly(vinylene carbonate) matrix using a single solvent. Electrostatic interactions between lithium salts and the polymer drive self-organized dual phases that combine mechanical robustness with efficient ion transport. The resulting PVC electrolyte achieves a tunable ionic conductivity from 0.20 to 0.92 mS/cm at 25 °C and a high lithium-ion transference number of 0.78. Li|PVC-24h | LiFePO_4_ cells achieve 121.4 mAh/g at 5 C (12 min) with 90% capacity retention after 4000 cycles, demonstrating a scalable approach for high-performance polymer electrolytes.

## Abstract (web summary)

The growing demand for portable electronics calls for lithium-ion batteries with higher energy density and longer cycle life. Here, the authors develop a facile phase-separation strategy to tailor polymer electrolytes through precise control of polymerization time, enabling high ionic conductivity, a large lithium-ion transference number and extended cycling stability in lithium metal batteries.

## Introduction

The increasing prevalent portable and consumer electronic devices demand lithium-ion batteries (LIBs) with high-energy-density and long cycle life. While, the development of high-energy-density LIBs is still constrained by fire and explosion risks, which is caused by the inflammability characteristic of liquid electrolytes^1^. Replacing liquid electrolytes with nonflammable solid-state electrolytes (SSEs) is a promising solution^2^. Among various SSEs, solid polymer electrolytes (SPEs) show the merits of flexibility, adjustable molecule structures and lightweight. Yet, conventional SPEs always suffer from drawbacks such as low ionic conductivity at room temperature, narrow electrochemical stability window, and poor electrode interface compatibility, hindering the commercialization of polymer-based lithium metal batteries significantly^3–8^.

In-situ polymerization has proven to be an effective strategy to enhance the interfacial contact between electrode materials and SSEs and has been widely employed in poly(diol oligomer), poly(vinylene carbonate) (PVC), poly(propylene carbonate), and poly(acrylonitrile-co-ethylene carbonate) systems^9–15^. Over the past decade, significant progress has been made in tailoring the polymer phase structures for improving the ionic conductivity of SPEs^16^. Lee et al. developed a mechanically robust, elastomeric bi-continuous SSE featuring a three-dimensional interconnected plastic-crystal phase with enhanced ionic conductivity^17^. Wu et al. reported an intrinsically stretchable ionic conductor that maintains stable ion transport under strain via polymerization-induced phase separation (PIPS)^18,19^.

Phase separation provides an effective route to integrate the complementary advantages of multiple phases in SPEs, enabling a balance between mechanical robustness and ionic mobility. However, conventional PIPS strategies typically rely on the introduction of additional components such as deep eutectic solvents or ionic liquids to form ion-conducting domains. These external additives not only increase material and processing costs but also complicate the system composition. Moreover, the phase separation process of PIPS is difficult to precisely control, particularly in terms of morphology and domain distribution, leading to poor reproducibility and limited scalability. These inherent limitations represent the central challenge that restricts the practical implementation of PIPS-based polymer electrolytes.

In this work, we propose a facile phase separation strategy by in-situ polymerization of lithium salt in a single-solvent without any additional phases, and the separation degree can be tailored precisely by simply adjusting the polymerization time. Electrostatic interactions between lithium salt anions and the polymer matrix regulate the solubility of PVC, driving the polymer phase out and separated from the precursor that respectively provide mechanical strength and efficient ionic transport. As a result, the PVC electrolyte exhibits an ionic conductivity of 0.60 mS/cm and a high lithium-ion transference number of 0.78. The symmetric Li|PVC|Li cells demonstrate stable lithium stripping/plating behavior for up to 6000 h at 30 °C. When applied in Li|PVC|LiFePO4 full cells, a discharge capacity of 121.4 mAh/g is achieved at 5 C and 30 °C with 90% capactity retention after 4000 cycles. This single-solvent polymerization strategy effectively addresses both interfacial and ionic transport challenges, providing a scalable pathway for high-performance polymer-based lithium metal batteries through self-optimized phase-separated architectures.

## Results

### Lithium salt-induced phase separation behavior

**Fig. 1:**
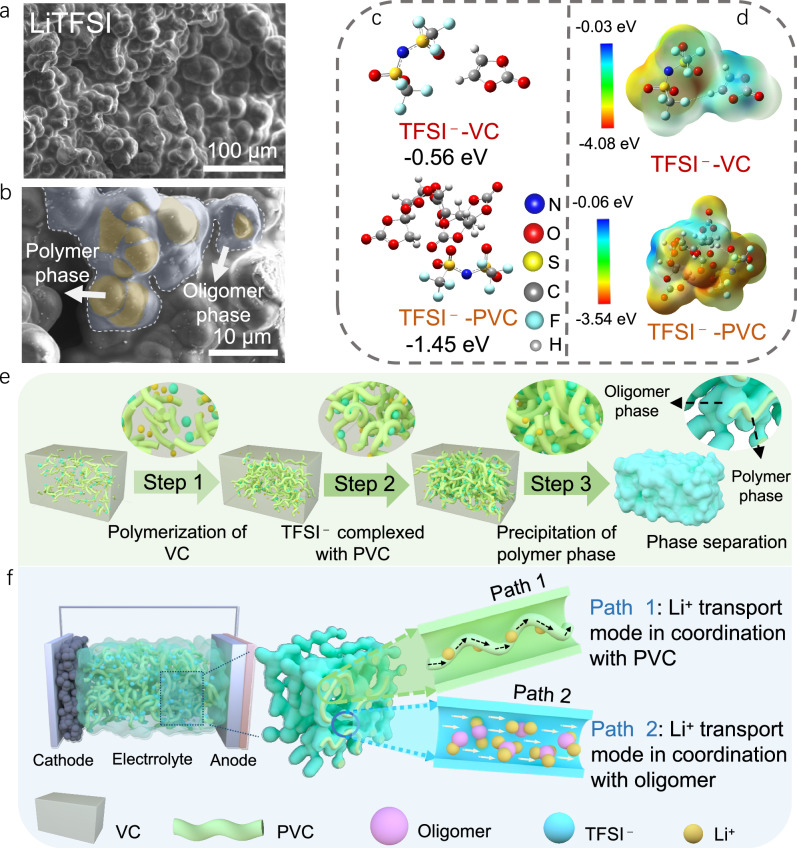
Schematics, calculation results, and morphological structures. **a**, **b** SEM images at different magnification of LiTFSI-regulated PVC electrolyte after in-situ polymerization. **c** The binding energy and **d** electron cloud density distribution probability of TFSI^−^ coordinated VC and PVC, respectively, the optimized geometry is provided in Supplementary Data 1. The schematic diagrams of (**e**) phase separation process and (**f**) lithium ion transport mode of LiTFSI-regulated PVC electrolyte.

The selection of lithium salts critically governs polymer electrolyte phase behavior through anion-mediated molecular interactions^20,21^. In this work, the effects of Lithium bis (trifluoromethanesulfonyl) imide (LiTFSI), Lithium difluoro (oxalato) borate (LiDFOB), LiPF_6_ and LiClO_4_ on the phase structure of the synthesized SPEs during in-situ polymerization process are investigated systematically, the anion volumes of TFSI^−^, DFOB^−^, ClO_4_^−^ and PF_6_^−^ are 147, 99, 55 and 69 Å^3^, respectively (Table S1)^22,23^. PVC electrolytes are synthesized by free radical polymerization of VC, the polymerization reaction equation and optical images of electrolyte are shown in Fig. S1a, b. The signal of the -C=C- peak (~1650 cm⁻¹) almost disappears in FT-IR spectroscopy (Fig. S2), while the characteristic peaks of -C=O (~1800 cm^−1^) and C-O-C (~1160 cm^−1^) remain. This indicates that the polymerization involves the consumption of -C=C-, without altering the fundamental molecular structure of VC^19^. As shown in ^1^H NMR spectra (Fig. S3a), the strong signal at 7.75 ppm corresponds to the protons of -CH=CH- in the VC molecular^24^. The new signal appeared at 5.35 ppm after polymerization corresponds to the protons of -CH-CH- in the PVC molecular. In ^13^C liquid phase NMR spectra (Fig. S3b), the carbon signals of VC at 135.0 and 153.8 ppm are ascribed to the protons of -CH=CH- and -C=O in the VC, respectively^24^. The new signals appeared at 76.0 and 152.3 ppm indicate the successful formation of PVC after polymerization. And the retained VC signal after polymerization implies the presence of oligomers in PVC electrolyte. Figure S4 show the phase behavior evolution by different anions during in-situ polymerization, the LiTFSI-regulated electrolyte consists of granular polymer and there is a precipitation process of solids during polymerization process while other anions-regulated electrolytes exhibit uniform polymerization without granular solid precipitation. Besides, the microstructures of LiTFSI-regulated PVC electrolyte from scanning electron microscope (SEM) show obvious phase separation characteristics of continuous phase enveloping granular phase (Fig. 1a, b), whereas that regulated by LiDFOB, LiClO_4_, and LiPF_6_ anions show homogeneous morphology (Fig. S5a–f).

The specific modes of interactions between TFSI⁻ and PVC/VC are further investigated. Raman spectroscopy results of the produced various PVC electrolytes reveal that with prolonged polymerization time, the -CF₃ and -S-N-S- groups in TFSI⁻ exhibit significant synchronous shifts, whereas the -C-O-C- groups in VC and PVC do not shift correspondingly (Fig. S6a–c). This indicates that TFSI⁻ is unlikely to interact directly with the carbonate groups in PVC/VC. Therefore, the Raman peak shifts of TFSI⁻ are mainly attributed to electrostatic interactions with the =C-H/-C-H groups in VC and PVC, forming weak hydrogen bonds, which are further enhanced as the polymer phase content increases by increasing polymerization time. To further elucidate the interaction mechanism between TFSI⁻ and PVC/VC, PVC electrolytes with varying concentrations of LiTFSI have been prepared. As shown in Fig. S7, the chemical shift of H 1 s in PVC/VC significantly moves upfield with increasing lithium salt concentration, which is associated with the coordination between TFSI⁻ and hydrogen atoms. This finding is also corroborated by 2D NMR heteronuclear correlation spectroscopy, which demonstrates a distinct interaction between H and F atoms. Notably, cross-peaks appear between F atoms (from TFSI⁻) and the H atoms (from -CH=CH- in VC and the resulting -CH-CH- in PVC) (Fig. S8), providing direct evidence of the interaction between TFSI⁻ and both PVC and VC.

The strength for the formed hydrogen bond primarily depends on the electropositivity of the hydrogen atom, and stronger electropositivity enhances the electrostatic attraction with electronegative atoms such as F, facilitating hydrogen bond formation. Compared to the sp²-hybridized carbon (=C-H) in VC, the sp³-hybridized carbon (-C-H) in PVC exhibits lower electronegativity, resulting in a weaker attraction for the electron cloud of the hydrogen atom. This allows the hydrogen atom to retain a higher share of the electron cloud. More importantly, the lower electronegativity of carbon leads to reduced polarity in the C-H bond, which consequently increases the partial positive charge on the hydrogen atom. This enhanced partial positive charge promotes the formation of weak hydrogen bonds with highly electronegative F atoms in TFSI⁻. Therefore, PVC-TFSI⁻ complex exhibits a higher binding energy (Fig. 1c).

Moreover, the distribution probability of electron cloud density represents the change of electron clouds around polymer molecules after complexing with anions. The complexation between TFSI^−^ and PVC results in a significant change of electronic cloud density around the polymer molecules (Fig. 1d and Fig. S9a–c). Compared with DFOB^−^, the distribution of the surrounding electron cloud density of PVC reduces after complexing with TFSI^−^, showing the weak electrostatic interaction between LiTFSI-regulated PVC and VC solvent, which leads to the precipitation of PVC from the solution and then induces phase separation.

The size and volume of anions will also affect their interaction with the polymer molecules. Anions with larger volume create higher spatial hindrance, reducing polymer solubility, while smaller anions readily infiltrate polymer matrices, enhancing phase miscibility. To validate this hypothesis, the phase evolution during polymerization is examined using structurally analogous lithium salts with varying anion sizes: Lithium bis fluorosulfonimide (LiFSI), LiTFSI, and Lithium bis (pentafluoroethanesulfonyl) imide (LiBETI) (molecular structures and results shown in Fig. S10). Under identical conditions, LiFSI fails to induce phase separation. Throughout the polymerization process, the mixture remains a transparent liquid without precipitation and ultimately solidifies into a translucent pale-yellow solid. In contrast, both LiTFSI and LiBETI generate granular precipitates after polymerization for 1 h, exhibiting clear phase separation behavior and finally forming powdered solid polymers. These results suggest that the molecular volume of lithium salts substantially influences phase separation behavior. Accordingly, the phase separation can be precisely tailored through different anions during in-situ polymerization process.

The schematic diagram of phase separation behavior of PVC during polymerization is shown in Fig. 1e, wherein PVC undergoes three stages of phase separation behavior over time. Initially, most VC polymerizes to form low-molecular-weight PVC oligomer phase, which could still dissolve in the precursor. With increased polymerization time, the interaction with LiTFSI promotes PVC to separate from the pecursor solution. As the PVC that has complexed with TFSI⁻ continuously precipitates from the precursor solution with polymerization process progresses, the newly synthesized PVC will continue to complex with the remaining TFSI⁻ and precipitate by means of the concentration difference, which causes TFSI⁻ to accumulate mainly in the polymer-rich phase. In the final stage of polymerization, the remaining unreacted monomers and low-molecular-weight polymers will form a continuous oligomer phase on the surface of the precipitated polymer phase, ultimately completing the phase separation process. And the corresponding Li^+^ transportation is schematically shown in Fig. 1f.

By in situ induced phase separation, the produced PVC polymer electrolyte can form a two-phase structure, including polymer and oligomer phases. In the polymer phase, Li^+^ can interact with PVC and transport through the polymer chain creep, while Li^+^ is coordinated with oligomer and transport rapidly in the continuous oligomer phase. The two modes of Li^+^ transport synergistically improve the ionic conductivity of the PVC polymer electrolyte.

### Phase separation behavior regulation

**Fig. 2:**
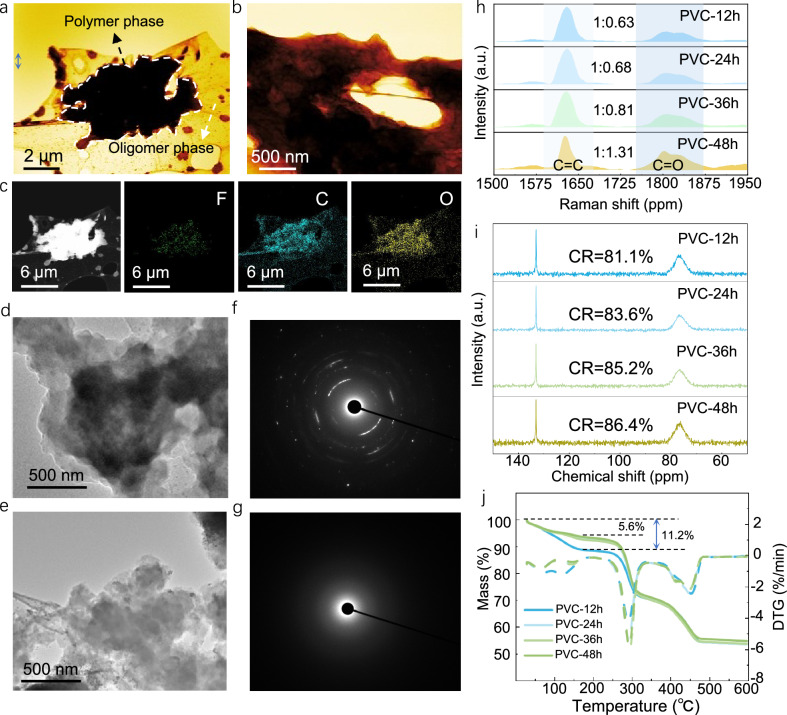
Morphology and structure characterization of PVC electrolytes. **a**, **b** TEM images (In order to highlight the contrast difference between the polymer phase and oligomer phase, the TEM images has been re-colored) and **c** EDS mappings of PVC-24h electrolyte. **d** Cryo-TEM image and **e** corresponding FFT pattern of oligomer phase. **f** Cryo-TEM image and **g** corresponding FFT pattern of polymer phase. **h** Raman spectra and **i** ^13^C NMR spectra of PVC electrolytes with different polymerization time. **j** TG and DTG curves of various PVC electrolytes with different polymerization time.

Based on the above results, LiTFSI, which induces phase separation behavior in PVC electrolyte, is selected as the lithium salt for subsequent studies on controlling phase separation degree. All electrochemical tests are conducted using the synthesized PVC electrolytes with LiTFSI as lithium salt. The microstructure of in-situ polymerized PVC electrolyte was examined using transmission electron microscopy (TEM). TEM images (Fig. 2a, b) reveal distinct phase-separated domains, confirming that polymer-rich phases are surrounded by oligomer-rich phases, and the phase diagram of the atomic force microscope (AFM) also shows obvious two-phase characteristics (Fig. S11)^25^. Elemental mappings further demonstrate the enrichment of F element (from LiTFSI) in polymer-rich phases (Fig. 2c), validating the anion-induced phase separation mechanism and its contribution to enhance Li-ion transference number. Cryo-TEM analysis at 100 K is further employed to visualize microphase separation. As shown in Fig. 2d, e, the persistence of biphasic structures at low temperatures is evidenced. Selected-area electron diffraction (SAED) patterns exhibit sharp diffraction spots in polymer-rich phase, indicating its pronounced crystallinity, while oligomer-rich phase maintains a highly amorphous state (Fig. 2f). This dual-phase stability corroborates their robust microphase-separated architecture (Fig. 2g).

PVC electrolytes with different polymerization time show different color, indicating their varying degrees of phase separation (Fig. S12). The relative intensity of C═C bond in Raman spectra shown in Fig. 2h reduces with increasing polymerization time gradually, and the relative intensity ratio of I_C=C_/I_C=O_ decreases from 1:0.63 to 1:1.36, which indicates the low content of residual oligomers with polymerization time. The corresponding conversion rate of PVC electrolytes at different polymerization time are analyzed using the ^13^C spectra of solid-state NMR corresponding to the -C=C- group. As shown in Fig. 2i, the conversion rate of VC reaches 81.1% after 12 h reaction, implying that the majority of highly reactive VC monomers has participated in the polymerization. When the polymerization time was extended to 24 and 48 h, the conversion rate slightly increases to 83.6% and 86.4%, respectively^26^. Solid-state NMR spectroscopy was employed to probe the structural evolution. As shown in ^19^F NMR spectra (Fig. S13a), a pronounced upfield shift occurs with prolonged polymerization time, arising from strengthened PVC-anion interactions that amplify the electron cloud shielding effect around ^19^F in TFSI^-^. The signal peak of ^7^Li also shows an upfield shift (Fig. S13b). This indicates that the coordination number between Li^+^ and TFSI⁻ has decreased, implies that more PVC and VC molecules participate in the solvation structure of Li⁺. As a result, the lone pair electrons from the carbonyl (C=O) groups in these solvent molecules are preferentially oriented toward the Li⁺ ions, compensating for the diminished electron shielding resulting from the weakened TFSI⁻ coordination, which facilitates lithium salt dissociation and enhancement of lithium-ion transference number^27^.

Small-angle X-ray scattering (SAXS) is further used to evaluate the interphase distance (d), where *d* = 2π/q and *q* denotes the position of the SAXS scattering peak. After subtracting the polyimide film background, the SAXS results (Fig. S14a, b) reveal that as the phase separation degree deepens, the diffraction peak intensity enhances gradually, and the peak position progressively shifts toward higher *q*-values. This further confirms that the PVC electrolyte forms a more ordered and compact structure with the enhanced phase separation degree. In SAXS tests at larger angles, only the amorphous diffraction halo of the PVC electrolyte is observed, and no significant changes occur during subsequent processing (Fig. S14c, d). This indicates that the polymerization time only affects the degree of phase separation in the PVC electrolytes without the formation of by-products. Thermal gravimetric analysis (TGA) measurements were performed to analyze the effect of phase separation degree on thermal stability of PVC electrolyte. As shown in Fig. 2j, PVC electrolytes experience three main weight-loss temperatures. According to the thermogravimetry differential electrochemical mass spectrometry (TG-DEMS) results (Fig. S15), the signals prior to 200 °C, H_2_O (fragments at m/z 18, 17, 16) and CO₂ (m/z 44, 12, 16, 22, 28, 45, 46) are observed within a broad mass loss regime, which is attributed to decomposition of oligomeric phase. Signals at approximately 300 °C result from the released H_2_O and CO₂ due to the depolymerization of the PVC electrolyte. Besides, signals between 400 and 500 °C ascribed to SO (m/z 48) and CO_2_ signals emerge due to thermal cleavage of LiTFSI. The relative molecular weight (Mw) values of PVC measured by GPC progressively increase from 62.7 K to 204.6 K as increasing the polymerization time (Fig. S16), indicating a decrease proportion of oligomer phase. Accordingly, changing the aggregation time can effectively regulate the degree of phase separation.

### The electrochemical properties of PVC electrolyte

**Fig. 3:**
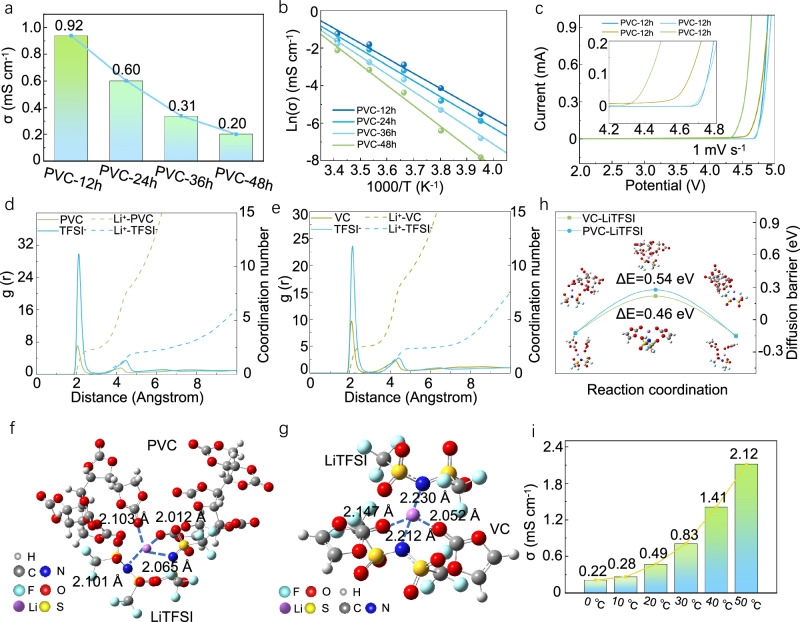
Characterization of lithium ion transport mechanism. **a** Ionic conductivity and **b** arrhenius plots at elevated temperatures of PVC electrolytes with different polymerization time. **c** LSV profiles of various PVC electrolytes with different polymerization time. RDF (solid lines) and coordination number (dashed lines) of **d** Li-PVC, **e** Li-VC and Li-TFSI^−^ ion pairs and molecular configuration of **f** polymer phase and **g** oligomer phase based on theoretical calculations in PVC electrolytes. **h** The diffusion barriers of Li between the different coordination structures in polymer phase and oligomer phase, The initial and final configurations of the MD simulation are available in Supplementary Data 3. **i** The temperature dependency of ionic conductivity of PVC-24h electrolyte.

The electrochemical impedance spectroscopy (EIS) measurements of PVC-based electrolytes with different phase separation degree under 25 °C are shown in Fig. S17, as the residual monomer content in the oligomer phase decreases (Fig. 3a), the ion conductivity of PVC electrolytes decreases from 0.92 mS/cm (PVC-12h) to 0.21 mS/cm (PVC-48h). This demonstrates that the oligomer phase plays a critical role in the improvement of ionic conductivity. To assess the low-temperature ion transport capability of PVC electrolytes, EIS data at low temperature (from −20 °C to 20 °C) was tested to analyze ion transport behavior of the designed electrolytes under extreme conditions. As shown in Fig. S18a–d, the ionic conductivity of PVC electrolytes correlates directly with both the degree of phase separation and content of oligomer phase. Notably, due to the enhanced crystallinity in high-molecular-weight polymer phases at low temperature, PVC-48h exhibits nearly an order of magnitude higher impedance than PVC-12h at −20 °C. Concurrently, activation energies for ionic conduction have been also calculated across PVC electrolytes with varying phase separation degree. The results demonstrate that as phase separation degree intensifies, the activation energy increases from 1.46 eV to 2.10 eV. This trend further confirms the low-temperature ion transport performance of oligomer-rich phase^10,17^. The results suggest that oligomers exhibit higher ion transport ability. Linear sweep voltammetry (LSV) shows that as increasing the extent of phase separation, the oxidation potential of the PVC-based electrolyte increases significantly from 4.30 V to 4.65 V (vs Li^+^/Li, Fig. 3c), while the oxidation current of VC electrolyte increases significantly at 3.3 V (Fig. S19). This indicates that the residual monomers in the oligomer phase will reduce the electrochemical stability of PVC electrolyte, and the polymer phase is responsible for the improved oxidation stability of the electrolyte. Namely, the designed phase-separated structure effectively integrates the high ionic conductivity of the oligomer phase with the electrochemical stability of the polymer phase, thereby imparting good overall performance to the electrolyte. AFM measurements shown in Fig. S20 indicate a progressive increase in Young’s modulus of the produced PVC electrolytes with the deepening of phase separation. The 2D modulus mappings exhibit two distinct regions with different mechanical properties. Despite the different phase-separation states, the surface of various PVC electrolytes maintains a consistent smooth morphology. Molecular dynamics (MD) simulations were carried out to investigate Li^+^ coordination configurations across different phase regions, and the phase migration energy barriers were also calculated to analyze their kinetic differences. The results of MD simulation reveal distinct Li^+^ solvation environments between polymer-rich and oligomer-rich phases. Notably, the structural homology between VC and PVC results in nearly identical first-shell solvation structures and coordination numbers of Li^+^ in both phases (Fig. 3d, e and Fig. S21a), indicating analogous ion transport mechanism. Crucially, the oligomer phase exhibits longer Li^+^-solvent coordination bond length (2.16 ± 0.11 Å) than polymer phase (2.07 ± 0.06 Å) (Fig. 3f, g and Fig. S21b), facilitating to enhance ion mobility and accelerate desolvation kinetics at interfaces.

Regardless of phase composition (single/mixed phases), as the competitive coordination effects of PVC-TFSI⁻, the Li⁺-TFSI⁻ interaction exhibits weakened coordination strength and an increased spatial distance, persistently lower anion coordination number is maintained within the primary solvation sheath. The reduced interaction between Li⁺ and TFSI⁻ lowers the desolvation energy barrier of TFSI⁻, which promotes the preferential desolvation of TFSI⁻ at the electrode-electrolyte interface to form a SEI layer rich in inorganic components. Diffusion barrier calculations quantitatively confirm faster Li^+^ transport through oligomer domains, exhibiting a substantially lower energy barrier (0.46 eV) than the polymer phase (0.54 eV), as shown in Fig. 3h. Pulsed Field Gradient Nuclear Magnetic Resonance (PFG NMR) spectroscopy was employed to quantify the diffusion coefficients of lithium (${{{{\rm{D}}}}}_{{7}_{{Li}}}$, corresponding to Li^+^) in various PVC-based electrolytes (Fig. S22a). As illustrated in Fig. S22b, ${{{{\rm{D}}}}}_{{7}_{{Li}}}$ progressively increases with the phase separation degree. Meanwhile, PFG-NMR measurements reveal a gradual decrease in the diffusion coefficient of ${{{{\rm{D}}}}}_{{19}_{F}}$ (from TFSI⁻) (Fig. S22c, d), indicating that the enhanced PVC-TFSI⁻ interaction restricts the migration of TFSI⁻ anions and reduces the Li⁺-TFSI⁻ coordination number. Consequently, the phase-separated PVC-based electrolyte exhibits a high lithium-ion transference efficiency. This reduction directly accounts for the improved ionic conductivity observed in oligomer-rich PVC electrolytes. The above results indicate that PVC SPE display good mechanical, thermal, and electrochemical stability^28^.

By taking the ionic conductivity and thermal stability into account, the 24 h is identified as the optimal polymerization time and PVC-24h electrolyte is used to assemble various batteries. Fig. S23a–c present the SEM images of PVC-24h electrolyte. As expected, the pores of glass fiber are filled with PVC electrolyte after wetting and polymerization, and the thickness of the PVC-24h electrolyte is about 100 μm. PVC-24h electrolyte shows well-connected pathways for ionic transport. After the in-situ polymerization, the intimate interface between the LFP positive electrode and PVC electrolyte can be observed (Fig. S23d). Namely, the PVC electrolyte has penetrated into the positive electrode adequately (Fig. S23e). XRD patterns of PVC electrolytes do not show any diffraction peaks within 10-90° (Fig. S24), indicating the amorphous structure of PVC electrolytes, which benefits to promote segmental vibration and partial relaxation of polymer chains. The ion conduction capability was tested through EIS from 0 to 50 °C (Fig. S25). As shown in Fig. 3i, the ionic conductivity of PVC-24h electrolyte increases as elevating temperature.

**Fig. 4:**
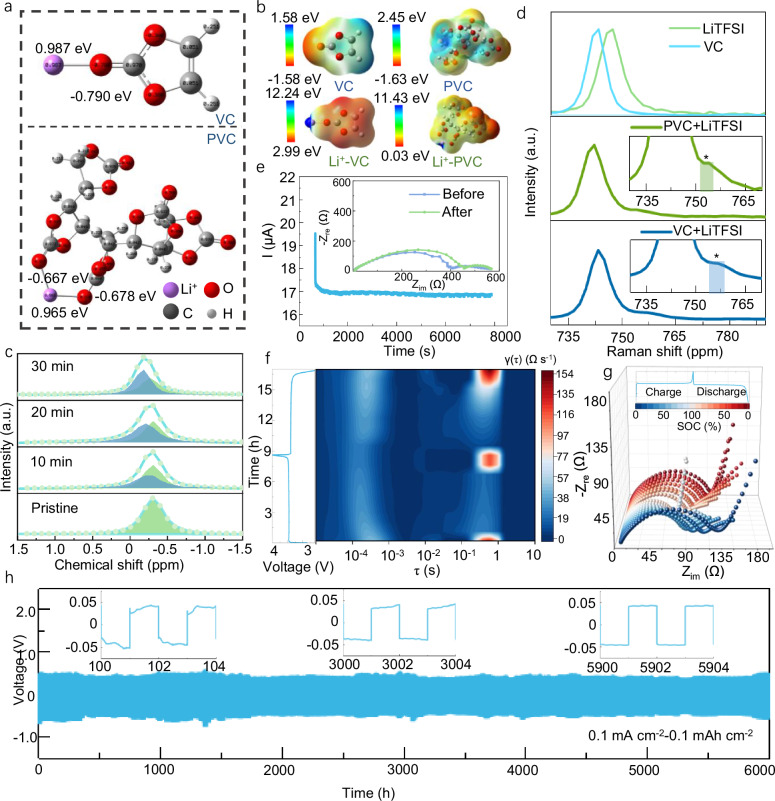
Characterization of lithium ion transport and interface dynamics. **a** The AVS calculation of VC and PVC and **b** the probability of electron cloud density distribution before and after VC and PVC complexation with lithium ions. The optimized geometry of VC and PVC are provided in Supplementary Data 5. **c** ^6^Li NMR spectra of PVC electrolyte after different discharge time of Li|PVC-24h|Li cells. **d** Raman spectra of LiTFSI with different coordination environments. **e** Li|PVC-24h|Li symmetrical battery chronograph current curve and EIS data before and after 10 mV polarization voltage. **f** Interfacial impedance evolution and **g** nyquist plots of Li|PVC-24h|LiFePO_4_ cell during the first cycle at different state of charge. **h** The corresponding charging/discharging curves and Cycling performance of Li|PVC-24h|Li symmetrical cell.

In order to explore the mechanism of Li^+^ transport in PVC electrolyte, the interactions between Li^+^ and oligomer PVC/VC groups was investigated by theoretical simulations. The atomic valence state (AVS) of O from C=O group in VC is −0.790 eV (Fig. 4a), which is slightly higher than that of oxygen atom in PVC (−0.667 eV and −0.678 eV), indicating that the Li^+^ tends to interact with VC rather than with the PVC^14,29^. The probability of electron cloud density distribution of VC and PVC was calculated by Gaussian software (Fig. 4b). Natural bond orbital charge indicates that Li^+^ trends to interact with both VC and PVC. However, the dispersion of PVC polymer weakens the electron cloud density around Li^+^. The peak at −0.3 ppm in ^6^Li NMR spectra represents the interaction between Li^+^ and C=O in VC. As shown in Fig. 4c, the new signal peak appeared at −0.1 ppm during cycling indicates the decreased electron cloud density around Li^+^, which corresponds to the interaction between Li^+^ with C=O in PVC^30^. These results confirm that Li^+^ transportation takes place through two different paths in PVC electrolyte (Fig. 1f).

The lithium ion transference number (${t}_{{{Li}}^{+}}$), a critical parameter governing Li deposition uniformity, was systematically investigated. Raman spectroscopy reveals LiTFSI dissociation dynamics in PVC-24h electrolyte: the TFSI⁻ vibration peak shifts from 748 (Li^+^-coordinated) to 754 cm⁻¹ (free anion), indicating enhanced ion-pair dissociation through PVC-TFSI⁻ interactions (Fig. 4d). This molecular-level modification enables higher ${t}_{{{Li}}^{+}}$ of 0.78 (Fig. 4e), attributed to TFSI⁻ immobilization via strong polymer-anion electrostatic interactions. Interfacial kinetics in Li|PVC|LiFePO4 cells were evaluated through in situ EIS (Fig. 4f, g) at different state of charge (SOC) with distribution of relaxation times (DRT) analysis (Fig. S26) during the initial charge-discharge cycle. The DRT profiles consistently exhibit two dominant relaxation processes. A characteristic peak at 10⁻³–10⁻² s (attributed to R_SEI_) corresponds to CEI/SEI formation dynamics, the other peak centered at 0.1 s (assigned to R_CT_) directly reflect interfacial Li⁺ charge-transfer kinetics. Critically, significantly reduced impedance during the voltage plateau clearly demonstrates good interfacial charge-transfer capability of the produced PVC-24h electrolyte. Furthermore, R_SEI_ maintains good stability throughout the whole cycling, conclusively verifying that no deleterious side reactions, compromising electrode-electrolyte interfaces, take place^31,32^. Fig. 4h depicts the cycling performance of the constructed Li|PVC-24h|Li symmetric cell at 0.10 mA cm^−2^ and 0.10 mAh cm^−2^. The slight and synchronous voltage fluctuation could be observed in the initial deposition/striping process, during the subsequent long-term cycling, the voltage fluctuation is effectively reduced. In addition, there is no observable short circuit phenomenon after cycling 6000 h. The rate capability of Li|PVC-24h|Li cell was evaluated at different current densities (Fig. S27), which demonstrates stable performance untill a current density and deposition capacity of 1 mA cm⁻² and 1 mAh cm⁻², with minor voltage fluctuations. This improved stability is attributed to the relatively high mechanical strength of the polymer phase, showing that the PVC-24h electrolyte with high ionic conductivity and *t*_Li_^+^ can induce uniform lithium deposition and ensure good compatibility with lithium negative electrode.

### Battery performance and interface stability

**Fig. 5:**
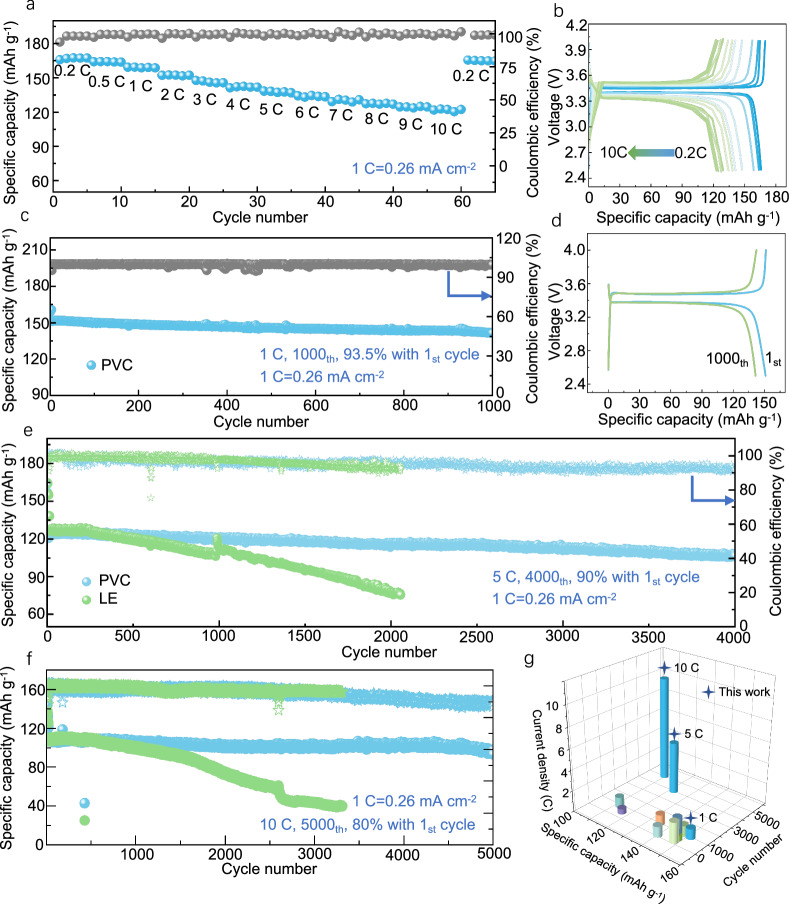
Electrochemical performance. **a** Rate performance and **b** corresponding charge-discharge curves of Li|PVC-24h|LiFePO_4_ full cells at 30 °C. **c** Cycle performance and **d** charge-discharge curves at 1st and 1000th cycles of Li|PVC-24h|LiFePO_4_ cell at 1 C and 30 °C. Cycle performance of Li|PVC-24h|LiFePO_4_ and Li|LE|LiFePO_4_ full cells at (**e**) 5 C and **f** 10 C under 30 °C. **g** Comparison of the electrochemical performance of Li|PVC-24h|LiFePO_4_ full cell in this work with other cells using polymer-based electrolytes reported previously, the source of the literature data shown in this figure can be found in Supplementary Information, Table S2.

The Li|PVC-24h|LiFePO_4_ full cell was assembled to investigate the electrochemical properties. Figure S28 reveals the obvious anodic peak at 3.6 V and cathodic peak at 3.3 V in CV curves of Li|PVC-24h|LiFePO_4_ cell, which is related to the extraction and insertion of Li^+^, respectively. The smooth and overlapped CV curves for the first five cycles demonstrate good electrochemical reaction reversibility. As shown in Fig. 5a, b, the Li|PVC-24h|LiFePO_4_ full cell displays good rate performances at 30 °C, which exhibit high specific capacity of 167.4 mAh/g at 0.2 C and of 121.9 mAh/g at 10 C. Figure 5c shows the cycling performance of Li|PVC-24h|LiFePO_4_ full cell at 0.2 C for the first three cycles and then at 1 C in the following cycles. It delivers a high reversible capacity of 151.3 mAh/g with a capacity retention of 93.5% after 1000 cycles at 30 °C. The charge–discharge curves at the 1st and 1000th cycles possess similar and minimal polarization (Fig. 5d). When cycled at a large current density of 5 C (Fig. 5e and Fig. S29a), the Li|PVC-24h|LiFePO_4_ full cell shows a high discharge capacity of 121.4 mAh/g with a capacity retention upto 90 % over 4000 cycles. While the assembled Li|LE|LiFePO_4_ full cell exhibits fluctuating CE and rapid capacity decay after 2000 cycles, which is caused by the accelerated dendrite formation during Li deposition/stripping. Even more, the Li|PVC-24h|LiFePO_4_ cell delivers a reversible specific capacity of 107.4 mAh/g after cycling 5000 times with a capacity retention of 80% at a very large current density of 10 C (Fig. 5f and S29b), which is ascribed to the high Li^+^ transfer ability of the obtained PVC-24h electrolyte by tailoring phase separation behavior.

The low-temperature operational capability is pivotal for practical battery applications. As shown in Fig. S30a, b, the Li|PVC-24h|LiFePO_4_ full cell demonstrates temperature-dependent capacity variation at 0.1 C, despite of increased polarization from restricted ion transport. Notablly, the capacity recovers to 160.3 mAh/g upon returning to 25 °C. Extended cycling at −10 °C achieves 118.6 mAh/g after 50 cycles (Fig. S30c), highlighting good low-temperature performance attributable to the electrolyte’s high ionic conductivity and Li⁺ transference number. Comparative analysis in Fig. 5g shows the electrochemical properties of Li|PVC-24h|LiFePO_4_ full cell has competitiveness compared to other polymer electrolyte-based lithium metal batteries reported previously across wide temperature ranges^9^^,^^10^^,^^28^^,^^33–37^. Practical viability is further demonstrated through flexible pouch cells powering 66-LED “XMU” displays (Fig. S31). The cells maintain operational integrity under mechanical stress (cutting, bending, wrinkling), showcasing good electrolyte flexibility and interfacial stability. These findings collectively validate PVC-24h as a promising electrolyte candidate for robust lithium metal batteries, addressing critical challenges in low-temperature operation while enabling flexible battery designs through its phase-separated architecture.

The interface compatibility between SPE and positive electrode/negative electrode critically governs the cycling stability of PLMBs. The density functional theory calculations (Fig. S32) reveal PVC (−8.80 eV) molecule possesses higher HOMO energy level than VC (−7.28 eV), indicating the better anti-oxidation capability of PVC^19^.

**Fig. 6:**
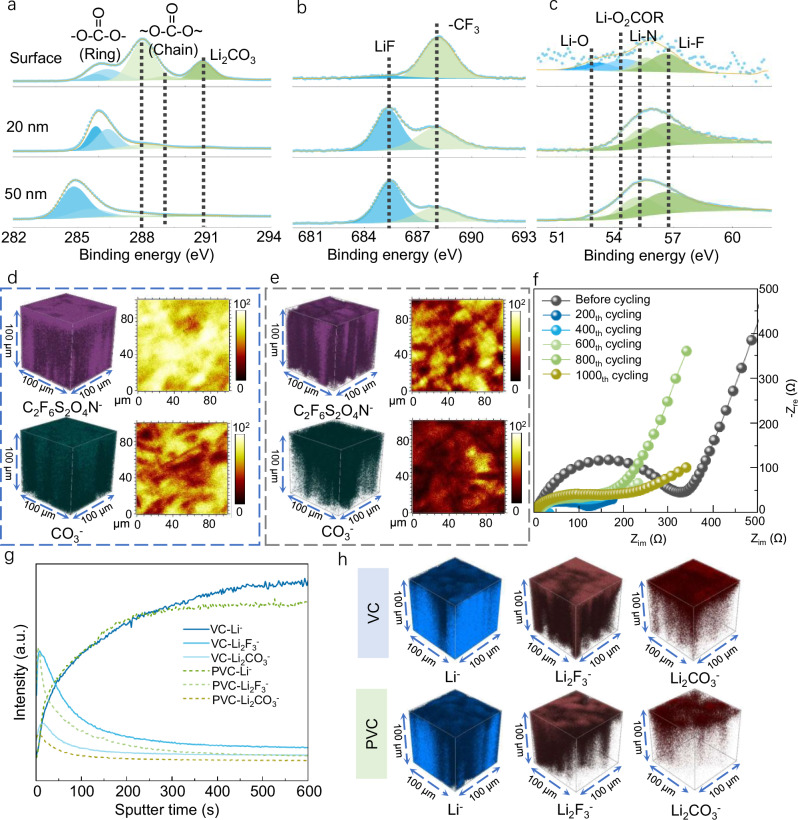
Analysis of the components of electrode-electrolyte interface. The **a** C 1*s*, **b** F 1*s*, and **c** Li 1*s* XPS spectra of LFP at varying etching depths after 200 cycles at 1 C in Li|PVC-24h|LiFePO_4_ cells. 2D and 3D distribution of C_2_F_6_S_2_O_4_N^⁻^ and CO_3_^⁻^ ionic fragments from PVC-24h electrolyte **d** before and **e** after 200 cycles at 1 C in Li|PVC-24h|LiFePO_4_ cells. **f** EIS curves of Li|PVC-24h|LiFePO_4_ cells at different cycles. **g** The intensity evolution and **h** 3D renders of Li^-^, Li_2_F_3_^-^ and Li_2_CO_3_^-^ in the TOF-SIMS sputtered volumes of VC-LiTFSI and PVC-LiTFSI induced SEI on the cycled lithium metal negative electrodes.

Simultaneously, the preferential localization of anions within the outer solvation sphere enables their targeted decomposition at the positive electrode interface, forming an inorganic-rich CEI that mitigates further oxidative degradation of PVC electrolyte. To validate this mechanism, XPS depth analysis was also performed on LFP positive electrode after 200 cycles (Fig. 6a–c). The C 1 *s* spectrum exhibits characteristic peaks at 287.8 eV (cyclic carbonate species), 289.0 eV (linear carbonate residues), and 291.1 eV (Li₂CO_3_). Concurrently, F 1*s* and Li 1*s* XPS spectra reveal signals from organic components (Li-O₂COR at 54.4 eV, -CF₃ at 688.2 eV) in CEI and residual LiTFSI^17,38–40^. After 20 nm and 50 nm sputtering, the signal of organic components (C-O/C=O) decreases, while the inorganic-dominated signals enhance. This conclusively demonstrates that the CEI consists primarily of inorganic species derived from LiTFSI decomposition. Such a CEI layer rich in inorganic components significantly enhances electrochemical stability by effectively suppressing continuous electrolyte oxidation during extended cycling. To investigate the electrochemical stability between PVC electrolyte and the lithium metal negative electrode, the surface components of PVC-24h electrolyte before and after cycling were measured by XPS. Before cycling, the peaks located at 284.8, 286.0, 287.8, 291.1 and 293.0 eV in the C 1 *s* spectrum are assigned to C-C/C-H, C-O, -CO_3_ bonds in cyclic carbonate species of PVC/VC, and -CF_3_ in LiTFSI, respectively (Fig. S33a). Meanwhile, the peak at 688.5 eV in the F 1*s* XPS spectrum in Fig. S33b and that at 401.7 eV in the N 1*s* XPS spectrum in Fig. S33c correspond to TFSI^−^ from LiTFSI^17,38–40^. After 200 cycles, the signal intensity from LiTFSI (peaks at 293.0 eV in C 1*s* spectrum and at 401.7 eV in N 1 *s* spectrum) decreases obviously, demonstrating the decomposition of LiTFSI. Furthermore, the peaks at 685.9 eV in F 1*s* spectrum and 399.7 eV in N 1*s* spectrum correspond to LiF and Li_3_N, indicating that the decomposed of LiTFSI. Meanwhile, the new stronger signal peak at 289.0 eV for -CO_3_ bond in linear carbonate can be observed, which is attributed to the full decomposition PVC/VC, leading to the formation of organic-rich SEI that benefits uniform lithium deposition^41^.

Time-of-flight secondary ion mass spectrometry (ToF-SIMS) analysis reveals dynamic evolution of the SEI layer in PVC-24h electrolyte. Pre-cycling 3D mapping shows homogeneous CO₃⁻ (PVC/VC derivatives) distribution, while C₂F₆S₂O₄N⁻ (TFSI⁻ fragments) exhibits surface aggregation (Fig. 6d and S34a). After cycling, surface C₂F₆S₂O₄N⁻ concentration decreases significantly, confirming TFSI⁻ preferentially decomposes into LiF/Li₃N (Fig. 6e and S34b). EIS of Li|PVC-24h|LiFePO_4_ cells (5 C, 30 °C) further corroborate good interfacial stability, with slight impedance evolution over 1000 cycles (Fig. 6f). Meanwhile TOF-SIMS analysis on the lithium metal surfaces of the assembled Li|VC-LiTFSI|Li and Li|PVC-24h|Li cells after identical cycling periods is carried out (Fig. 6g, h). The lithium metal surface from Li|PVC-24h|Li exhibits a thinner LiF layer than that from Li|VC-LiTFSI|Li, which can be attributed to the confinement of a significant portion of TFSI⁻ within the polymer phase, limiting its decomposition at the lithium metal surface. Concurrently, the reduced intensity and thickness of the Li₂CO₃ signal indicate that TFSI⁻ in the PVC electrolyte preferentially decomposes to form an inorganic-rich SEI, thereby suppressing further decomposition of solvent molecules. This demonstrates LiTFSI’s dual functionality: regulating phase separation during in-situ polymerization and promoting stable SEI formation through anion-modulated inorganic components. The lithium salt-induced oligomer/polymer phase separation strategy synergistically enhances both electrolyte performance and Li metal interface compatibility, providing a holistic approach for durable lithium metal batteries.

## Discussion

In summary, this study introduces a LiTFSI-mediated in-situ polymerization strategy that induces controllable phase separation to construct high-performance dual-phase SPEs. The designed architecture integrates a robust polymer matrix that enhances mechanical, thermal, and electrochemical stability with an interconnected oligomeric phase that enables efficient Li^+^ transport and uniform lithium deposition. Benefiting from the synergistic regulation of ionic conductivity (0.60 mS cm^−1^ at 25 °C, tunable up to 0.92 mS cm^−1^) and a high ${t}_{{{Li}}^{+}}$ of 0.78, the SPE achieves accelerated Li^+^ flux and suppressed concentration polarization, leading to good overall electrochemical performance. The Li|PVC-24h | LiFePO_4_ cells exhibit stable cyclability, retaining 93% capacity after 1000 cycles at 1 C and 90% after 4000 cycles at 5 C, as well as higher low-temperature capacity of 118.6 mAh g^−1^ at −10 °C/0.1 C. This work demonstrates a rational structural design concept for polymer electrolytes through controllable phase separation, achieving simultaneous optimization of ion transport and interfacial stability, and providing a practical pathway toward durable, high-performance lithium metal batteries operable under diverse conditions.

## Methods

### Preparation of electrolytes

LiTFSI, LiDFOB, glass fibers (Whatman GF/A) were purchased from DodoChem, all materials were used directly without any processing. LiPF_6_ was purchased from Acmec. LiClO_4_ was purchased from Sinopharm Chemical Reagent Co. VC (99%) and 2,2’-Azobis-(2,4-dimethylvaleronitrile) (ABVN) were purchased from aladdin. VC (1000 μL) and lithium salt (1 mmol) were mixed under magnetic stirring inside a glovebox to obtain a homogeneous mixture. Subsequently, ABVN (1 wt%) was added as an initiator to the solution and stirred for 1 h to form electrolyte precursor. Finally, the electrolyte precursor was dropped into borosilicate glass fibers and maintained at 60 °C for polymerization to obtain PVC electrolyte in an argon-filled glovebox. To maintain consistency, the electrolyte was obtained by disassembling the battery for various characterizations and transported under an inert atmosphere. LITFSI was selected as the lithium salt for the subsequent research on controlling the phase separation behavior. the related electrochemical tests were conducted based on the PVC electrolyte synthesized with LiTFSI.

### Preparation of LMBs

Li metal negative electrode with a diameter of 16 mm (450 μm), was purchased from Guangdong Canrd New Energy Technology Co., Ltd, stored in a in an Ar-filled glovebox (<1 ppm O_2_ and <1 ppm H_2_O) at room temperature. All materials were used directly without any processing.The LFP positive electrode was prepared using a slurry casting technique. The conductive carbon, PVDF, anhydrous N-methyl-2-pyrrolidone (NMP, 99.9% purity), carbon-coated aluminum (Al, 15 μm-thick) foil were purchased from the Guangdong Canrd New Energy Technology Co., Ltd, and used directly without any additional processing. The active material, acetylene black, and PVDF were dissolved in NMP with a weight ratio of 8:1:1 to form a slurry. The slurry was stirred for 12 h at room temperature using an automatic mixer, and then coated onto an aluminum foil current collector by doctor blade. The coated aluminum foil was dried in a vacuum oven at 90 °C for 24 h, the positive electrode was cut into discs with a diameter of 12 mm, and the mass loading of active material was 2.5–3 mg/cm^2^. The 2025-type Li|SPE|Li symmetry coin cells and Li|PVC-24h|LiFePO_4_ coin full cells were assembled via in-situ polymerization at 60 °C for 24 h in an Ar-filled glovebox (<1 ppm O_2_ and <1 ppm H_2_O). The assembled batteries were tested under a temperature of 30 °C without any additional pressure applied.

### Calculation

The structural optimization, vibration frequency, and electronic structure calculations of VC, LiTFSI and PVC were performed using the hybrid density functional theory B3LYP exchange−correlation functional with the def2-TZVP basis set as implemented in the Gaussian 16 program^42–44^. The electrolyte structure of VC and PVC was visualized, made by VMD^45^. Analyses of the molecular orbitals were based on the Multiwfn program.

### Materials characterization and instruments

The 3D schematic diagrams were created using Autodesk 3ds Max (version 2025, Autodesk Inc.) under an educational license. The crystal structures of the produced samples were characterized by X-ray diffractometer (XRD) with Cu-Kα radiation at 40 kV and 40 mA and Small Angle X-ray Scattering (SAXS) in HRESOL mode with Cu-Kα in 0.5 mm. The morphology and cross-section SEM images of the as-prepared electrolytes were analyzed by SU-70 at 5 kV and at 20 kV. The atomic and chemical composition of interface were studied by the X-ray photoelectron spectroscopy (XPS) using the ESCALAB 250 Xi spectrometer. The time of flight secondary ion mass spectrometry (TOF-SlMS) was carried out on IONTOF M6 equipped with a bismuth primary ion source and a Ar^+^ sputter source for probing the fragment ions. The 3D reconstruction graphs of various components were obtained on an area of 300 ×300 μm by using a 30 keV Bi^3+^ primary ion beam, followed by a 1.63 s/cycle sputter of a 500 × 500 μm area using 1 keV Ar^+^ ion beams in an interlaced mode. The 3D reconstruction graphs of various components were obtained on the area of 300 × 300 μm by using a 30 keV Bi3^+^ primary ion beam, followed by a 1.63 s/cycle sputter of a 500 × 500 μm area using 1 keV Ar^+^ ion beams in an interlaced mode. Liquid nuclear magnetic resonance (NMR) spectroscopy was employed to analyze the molecular structure of PVC electrolytes. VC and the polymerized electrolyte were dissolved in DMSO-d_6_ for liquid NMR testing. FTIR spectrometer (Perkin-Elmer) ARTICLE in the transmission mode from 500 to 4000 cm^−1^ was collected with 32 scans and a resolution of 4 cm^−1^. The atomic force microscope (AFM) images were acquired using a Bruker Dimension® Icon^TM^ system with a scanning range of 5 μm. SCANASYST-AIR probes were employed for topography measurements. All images are presented in height mode without post-processing except flattening. Raman spectra were measured on a HORIBA Lab RAM HR Evolution with a 532 nm argon ion laser. The solid-state NMR was performed using a Bruker Avance NEO 600 MHz spectrometer with a 3.2 mm probe head at a spinning rate of 20 KHz. Spectra were accumulated for 1024 scans with a cycle delay of 1 s, using a pulse width of π/2. Battery Test System (S4008, Newware Electronics) was used to measure galvanostatic cycling and rate performances of the PLMBs. The electrochemical tests covered in this article all feature two sets of parallel tests for each test. Cyclic voltammetry (CV) was tested at a scanning rate of 0.1 mV s^-1^ and the LSV curves of SPEs were tested at a scanning rate of 1 mV s^−1^ from 2.5 to 5.0 V (vs Li^+^/Li) by Autolab electrochemical workstation (NOVA 1.9). The electrochemical impedance spectroscopy (EIS) were tested by means of potentiostatic method within the frequency range of 10 MHz–0.01 Hz with an amplitude of 5 mV and 10 points per decade using stainless steel/SPEs/stainless steel sandwiching structure from 30 °C to 70 °C by Autolab electrochemical workstation (NOVA 1.9). The ionic conductivity (*σ*) of SPEs was calculated according to the following Eq. 1:

1 $${{{\rm{\sigma }}}}=\frac{{{{\rm{L}}}}}{{R}_{b}S}$$

*R*_b_ is obtained from AC impedance spectroscopy, L and S are the thickness and area of the SPEs. The activation energy is calculated by Arrhenius equation as shown in Eq. 2:

2 $${{{\rm{\sigma }}}}({{{\rm{T}}}})={\sigma }_{0}exp [-\frac{{E}_{a}}{{RT}}]$$

wherein ${\sigma }_{0}$ is the ionic conductivity, *T* is absolute temperature, *E*_*a*_ is activation energy, and *R* is the universal gas constant. And the Li-ion transference number is calculated by the Bruce-Vincent-Evans Equation as follows:

3 $${t}_{{{Li}}^{+}}=\frac{{I}_{s}(\Delta V-{I}_{0}{R}_{0})}{{I}_{0}(\Delta V-{I}_{s}{R}_{s})}$$

Where *I*_0_, *I*_s_, *R*_0_, and *R*_s_ are the initial and steady-state DC current and steady-state interface resistances, respectively. ∆V is the applied pulse potential of 10 mV.

## Supplementary information

Supplementary Information

Description of Additional Supplementary Files

Supplementary Data

Transparent Peer Review file

## Source data

Source Data

## Data Availability

All data generated or analyzed during this study are included in the published article and its Supplementary Information. Source data for all plots are included in the accompanying Source Data Excel file. Source data are provided with this paper.
