# Supplementary material for: Tailoring electrolyte phase separation for high-rate solid-state lithium metal batteries
Source: Nat Commun. 2026 Jun 8;17:7310. doi: 10.1038/s41467-026-74094-w (PMC13402737; doi:10.1038/s41467-026-74094-w)
Supplement: Supplementary file 1 — Supplementary Information [file 41467_2026_74094_MOESM1_ESM.pdf]

Supporting Information for

Tailoring electrolyte phase separation for high-rate solid-state lithium metal batteries

Shiyu Zhang[1], Jiantao Li[2,*], Benli Jiang[3], Guanyi Wang[4], Chengkun Zhang[1], Chengyu Wang[5], Xinchao Hu[1], Jie Shen[5], Ziyi Fang[1], Liang Lin[1], Guiyang Gao[1], Yuming Jin[1], Baisheng Sa[5], Laisen Wang[1,*], Jie Lin[1], Qingshui Xie[1,*] and Dong-Liang Peng[1]

[1]State Key Laboratory of Physical Chemistry of Solid Surface, College of Materials, Xiamen University, Xiamen 361005, China

[2]Department of Chemical and Biological Engineering, Northwestern University, Evanston, IL 60208, USA

[3]Division of Materials Science and Engineering, Boston University, Boston, MA 02215, US

[4]Department of Chemical and Paper Engineering, Western Michigan University, Kalamazoo, MI 49008, USA

[5]College of Materials Science and Engineering, Fuzhou University, Fuzhou 350100, China

[*] Corresponding author. E-mail: jiantao.li@northwestern.edu; wangls@xmu.edu.cn; xieqsh@xmu.edu.cn

Supplementary Figures

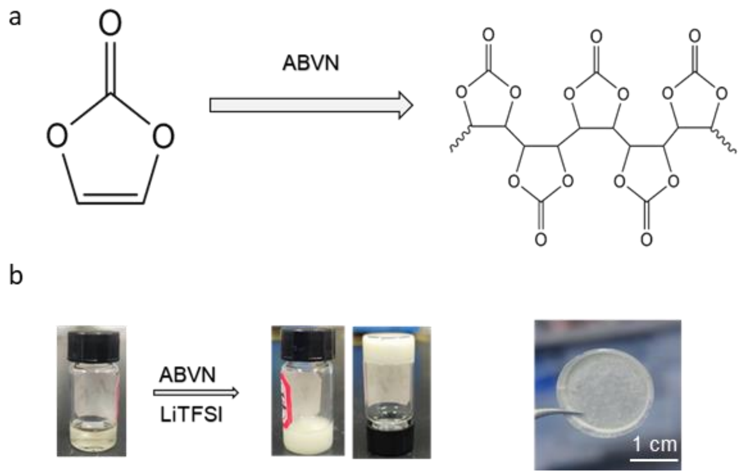

Figure S1. (a) Synthetic reaction mechanism of PVC polymer and (b) the corresponding optical images of PVC electrolyte.

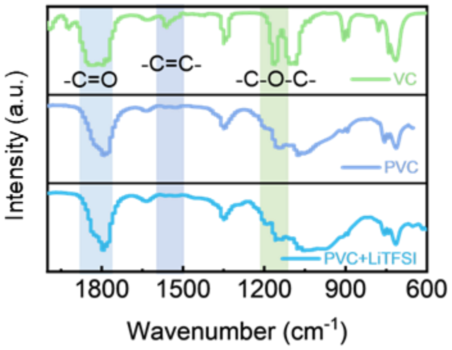

Figure S2. FTIR spectra of VC, PVC polymer and PVC-24h electrolytes.

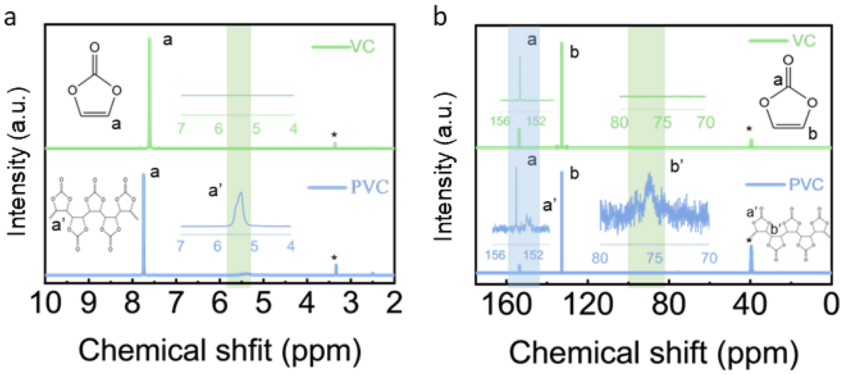

Figure S3. Liquid phase (a) ^{1}H and (b) ^{13}C NMR spectra of VC and PVC electrolyte.

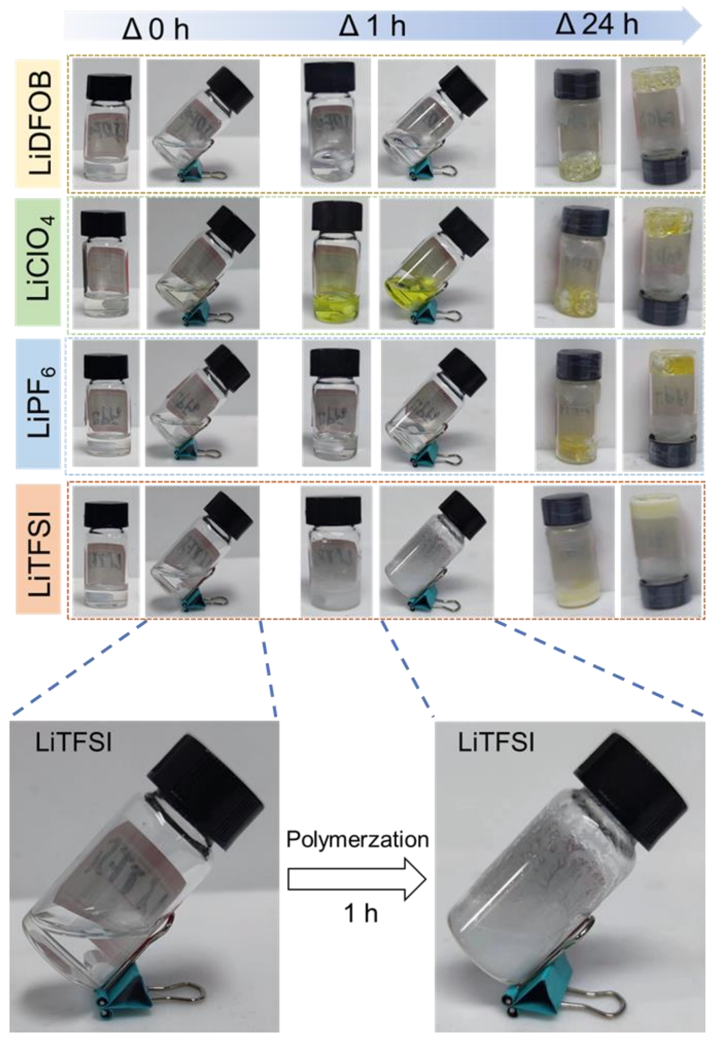

Figure S4. Optical images of PVC electrolytes regulated by different lithium salts at different polymerization time and the enlarged optical images of the precipitation of solid particles in LiTFSI-regulated PVC electrolyte after 1 h of polymerization.

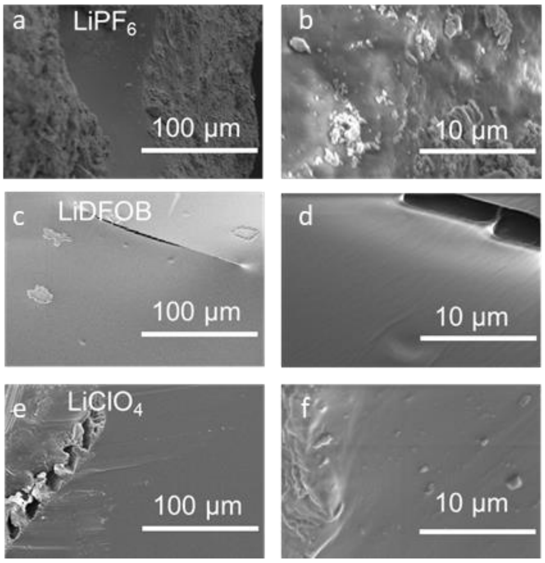

Figure S5. SEM images of the produced various PVC electrolytes with (a-b) $LiPF_6$, (c-d) LiDFOB and (e-f) $LiClO_4$ after in-situ polymerization.

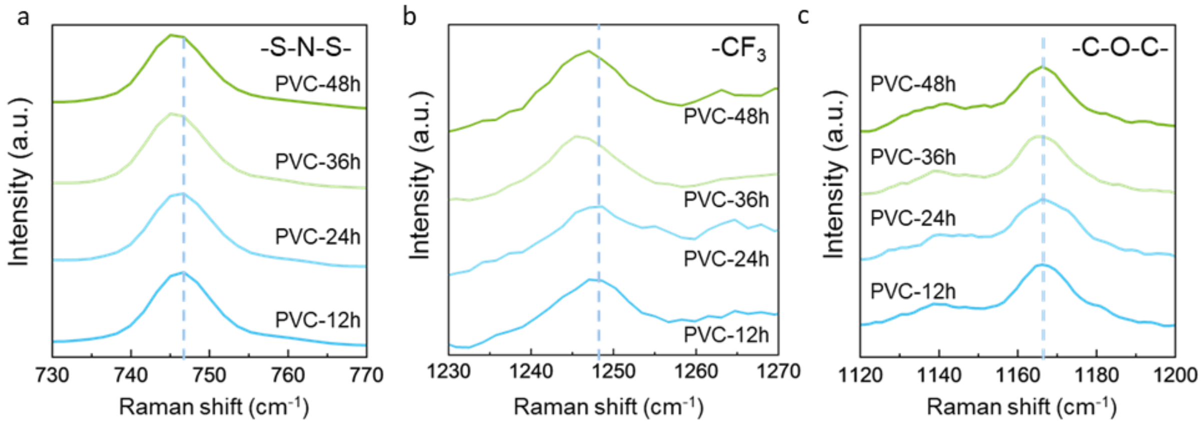

Figure S6. Raman spectra of (a) -S-N-S-, (b) -CF_3 and (c)-C-O-C- groups in various PVC electrolytes with different polymerization time.

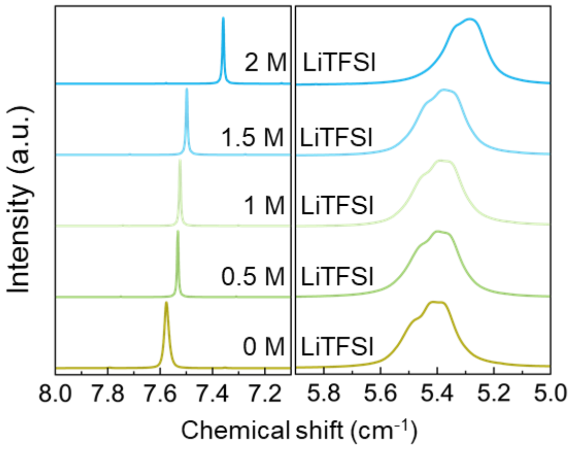

Figure S7. ^{1}H NMR spectra of PVC electrolytes with varying concentrations of LiTFSI.

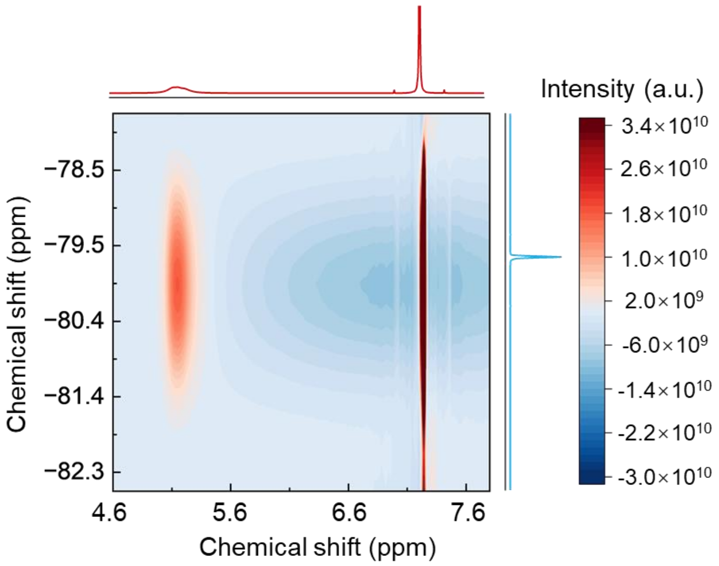

Figure S8. 2D ^{1}H–^{19}F HETCOR of PVC-24h electrolyte.

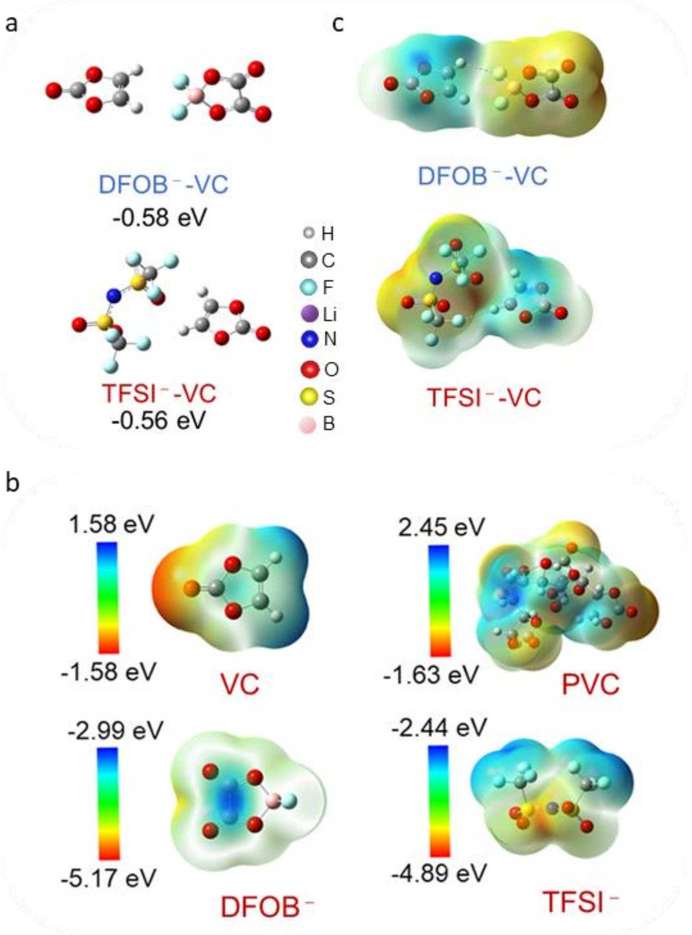

Figure S9. (a) The binding energy of the coordination of $DFOB^-$ with VC and PVC. Electron cloud density distribution probability for (b) VC, $DFOB^-$, PVC and $TFSI^-$. (c) Electron cloud density distribution probability for the coordination of $TFSI^-$ and $DFOB^-$ with VC, the optimized geometry of MLi_2O is provided in Supplementary Data 1.

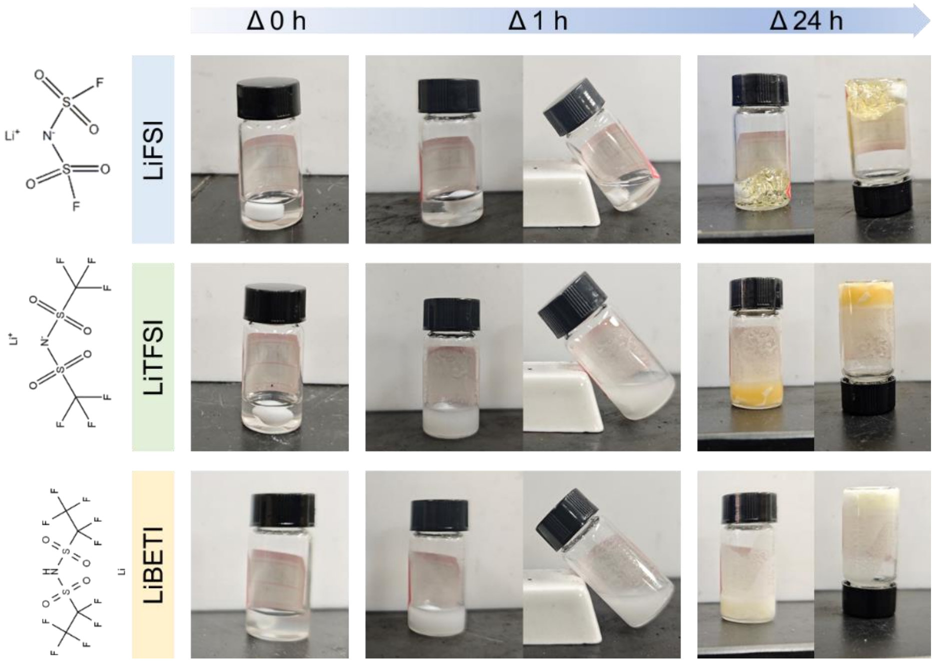

Figure S10. Optical images of PVC electrolytes regulated by different lithium salts at different polymerization time.

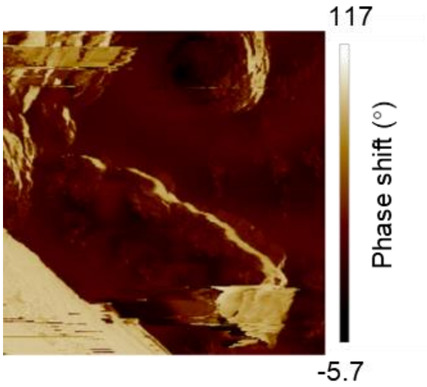

Figure S11. AFM image of PVC-24h electrolyte (scale bar is 5 μm)

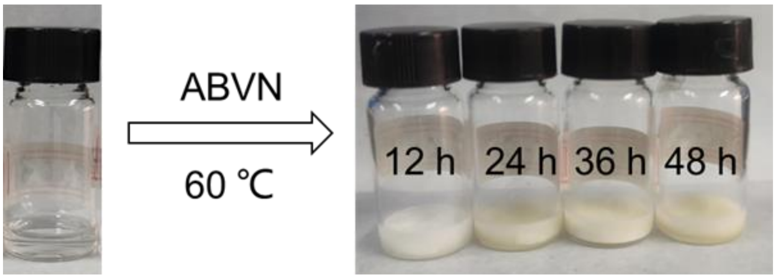

Figure S12. Optical images of PVC electrolytes with different polymerization time.

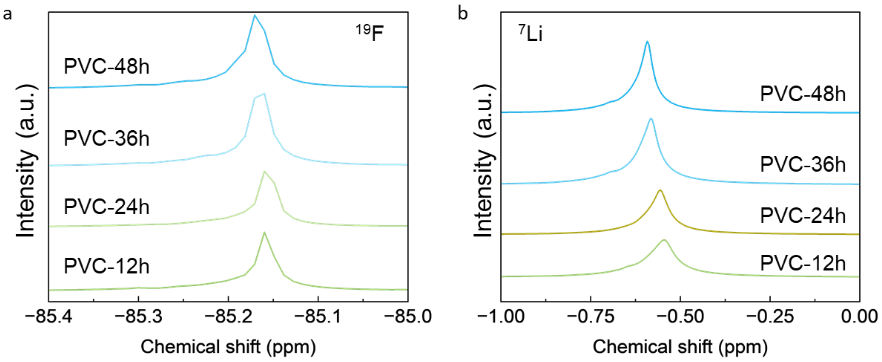

Figure S13. (a) ^{19}F and (b) ^{7}Li NMR spectra of PVC electrolytes with different polymerization time.

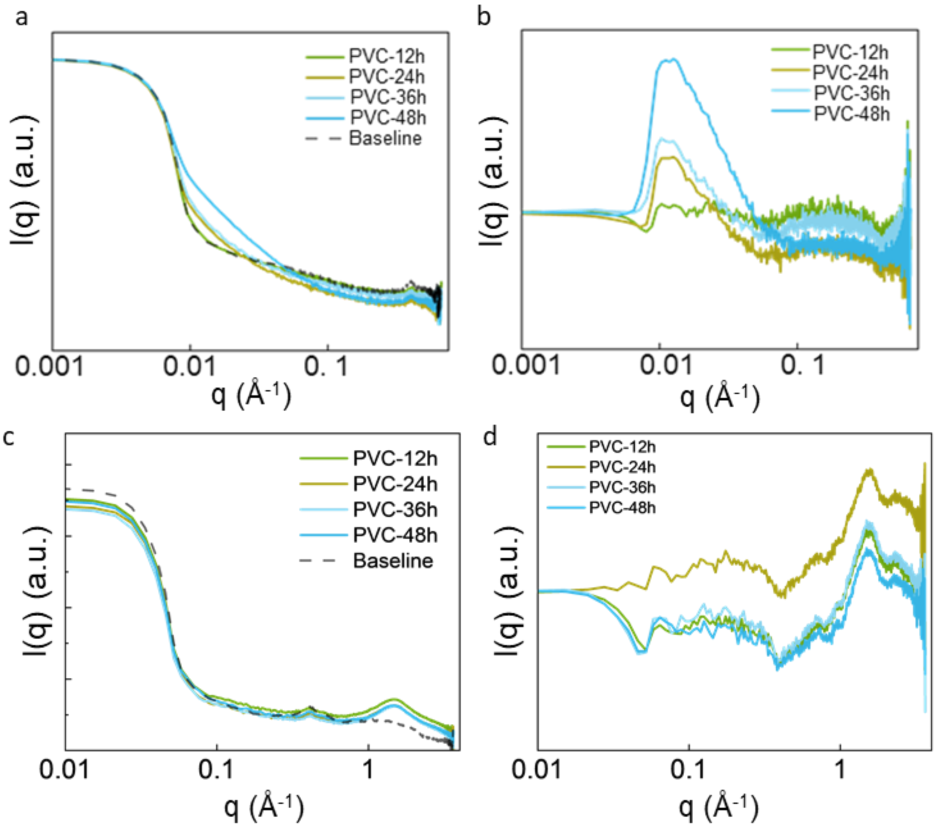

Figure S14. SAXS of various PVC electrolytes with different polymerization time (a) before and (b) after the baseline subtraction. SAXS at large angles of various PVC electrolytes with different polymerization time (a) before and (b) after the baseline subtraction.

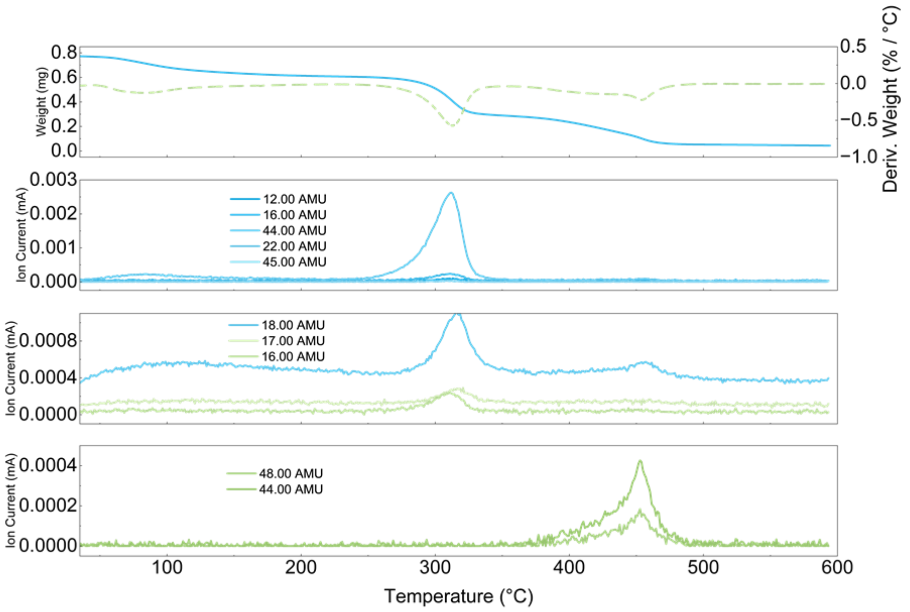

Figure S15. TG and DTG curves and corresponding in-situ DEMS results of PVC-24h electrolyte.

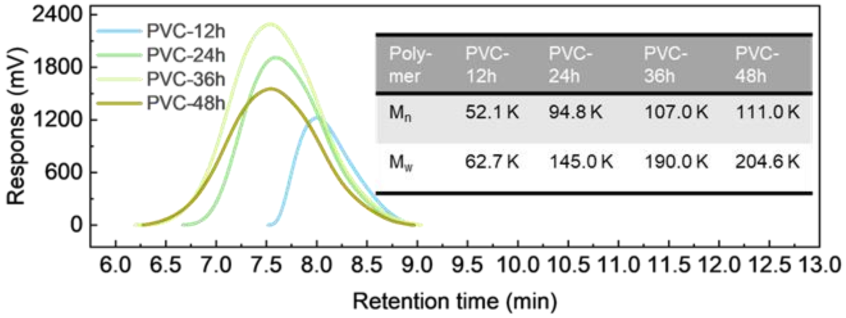

Figure S16. Molecular weight of PVC electrolytes with different polymerization time.

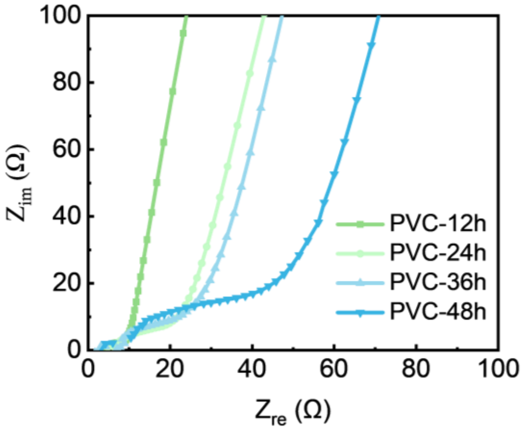

Figure S17. EIS curves of stainless steel (SS)|PVC|SS cells after different polymerization time at 25 ℃.

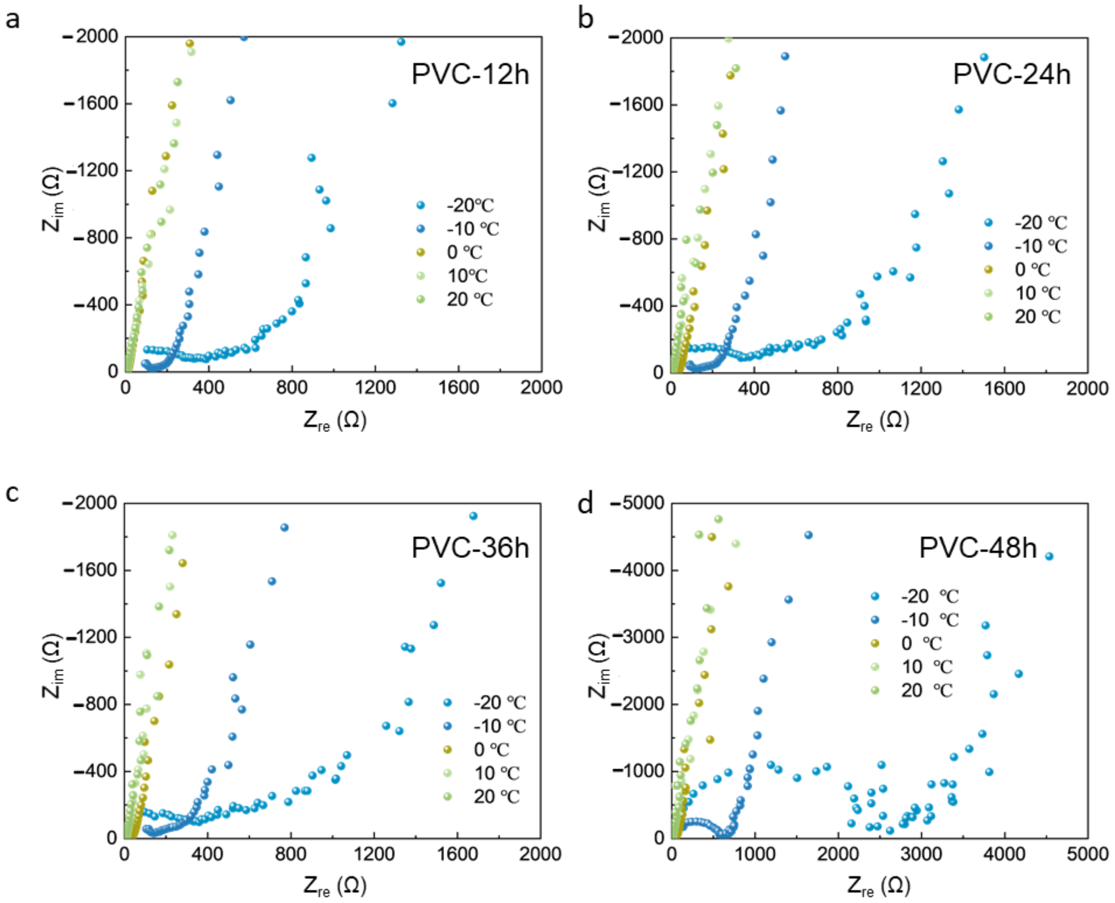

Figure S18. EIS curves of SS||SS cells with (a) PVC-12h, (b) PVC-24h, (c) PVC-36h and (d) PVC-48h from -20 to 20 ℃.

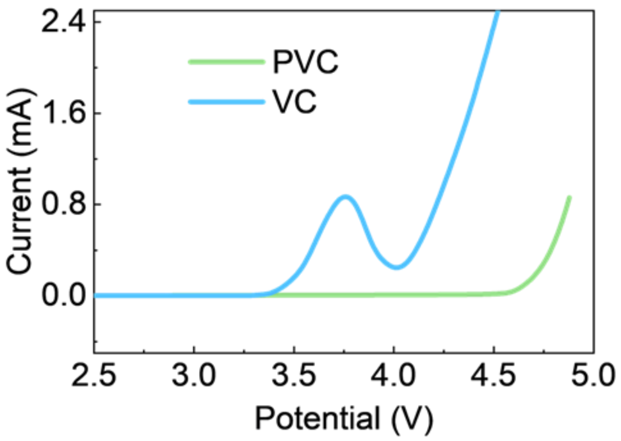

Figure S19. LSV profiles of VC and PVC electrolytes.

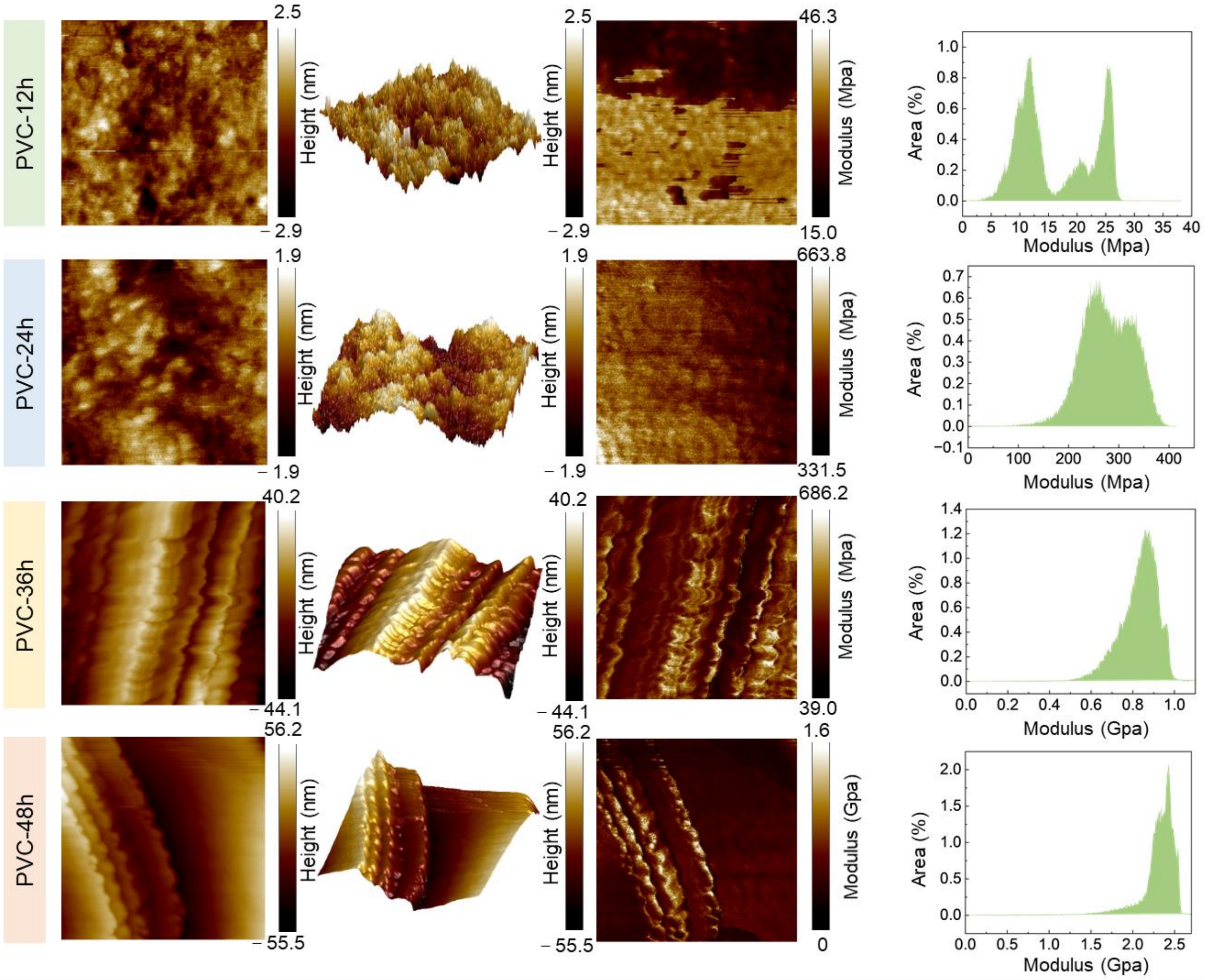

Figure S20. 2D, 3D AFM images and 2D Young's modulus distribution range of the produced PVC electrolytes with different polymerization time.

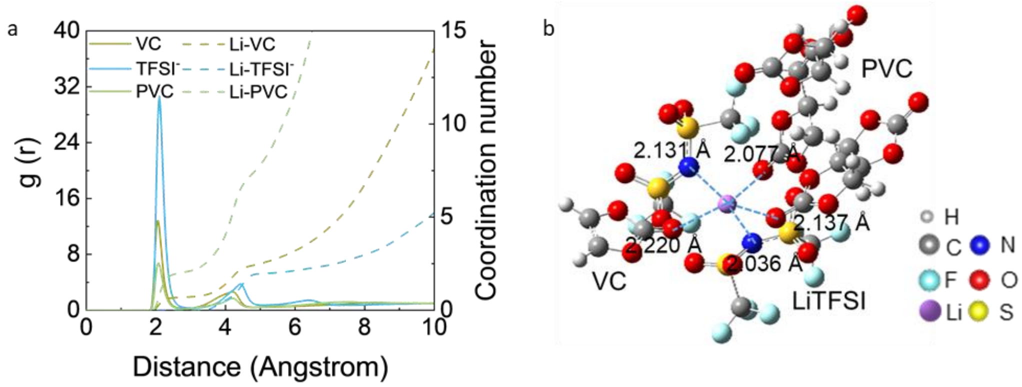

Figure S21. (a) RDF (solid lines) and coordination number (dashed lines) of Li-PVC, Li-VC and Li-TFSI- ion pairs and (b) molecular configuration in PVC electrolytes based on theoretical calculations, the PVC electrolyte configurations of the MD simulation are available in Supplementary Data 4.

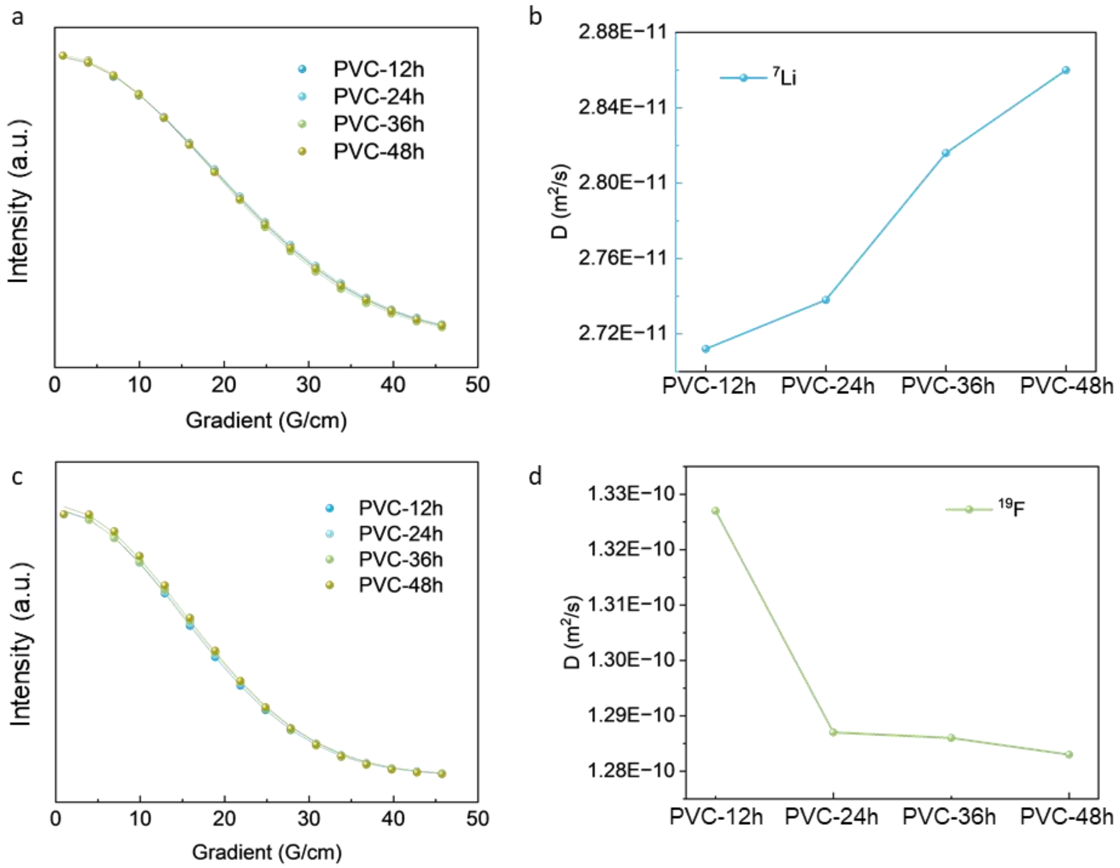

Figure S22. The PFG NMR echo decay profiles and diffusion coefficients for (a-b) ^{7}Li and (c-d) ^{19}F in PVC electrolytes with different polymerization time.

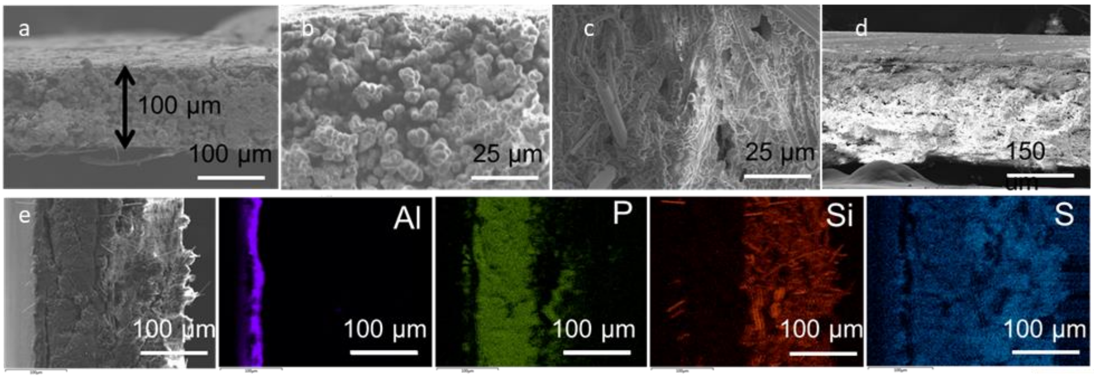

Figure S23. (a-c) Surface and cross section SEM images of PVC-24h electrolyte, (c) Surface, (d) cross-sectional SEM images and (e) the corresponding EDS mappings of the interface between the LFP cathode material and PVC-24h electrolyte after in-situ polymerization.

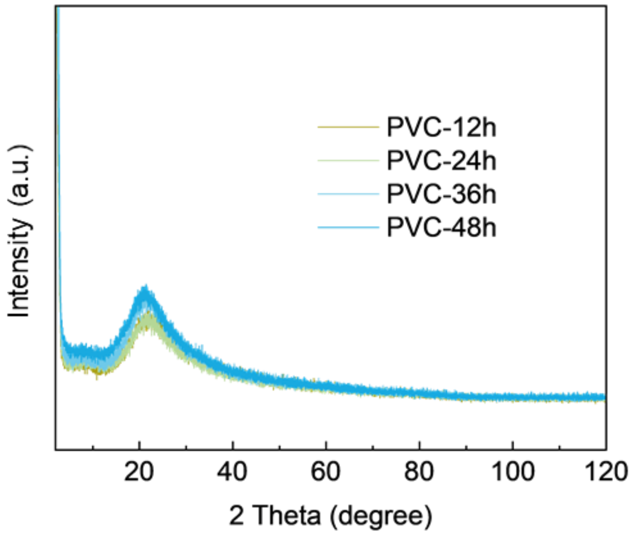

Figure S24. XRD patterns of various PVC electrolytes with different polymerization time.

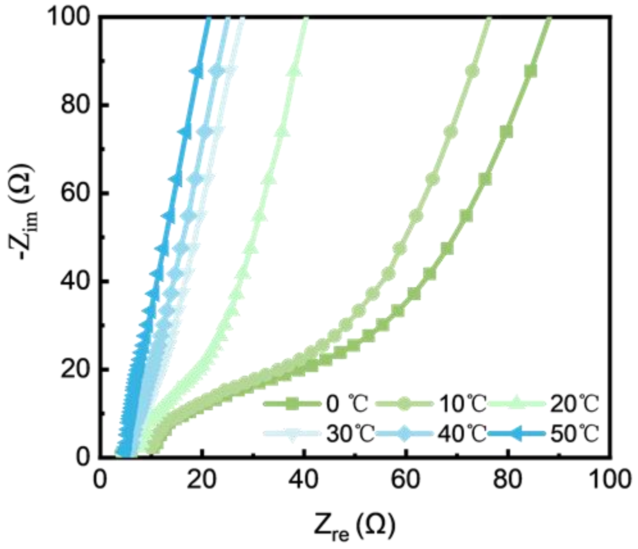

Figure S25. EIS curves of SS|PVC-24h|SS cell at different temperature.

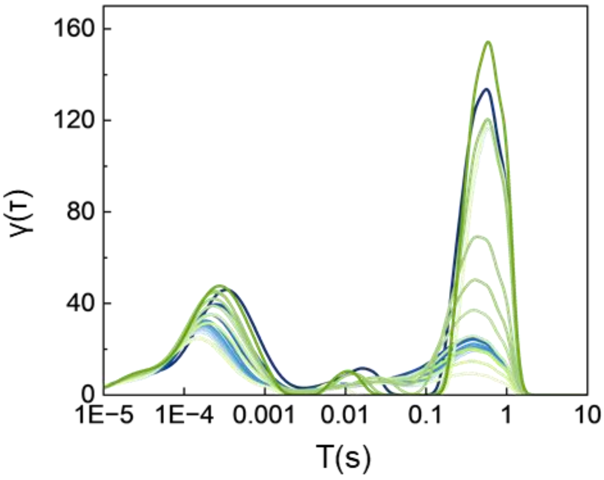

Figure S26. DRT profiles obtained from GEIS of Li|PVC-24h|$LiFePO_4$ cells.

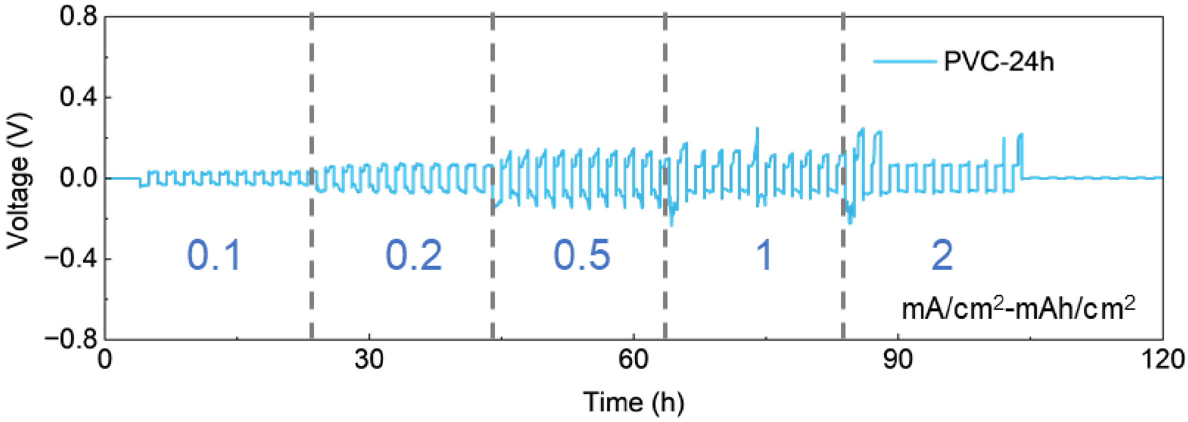

Figure S27. Rate performance of Li||Li symmetric batteries using PVC-24h electrolyte.

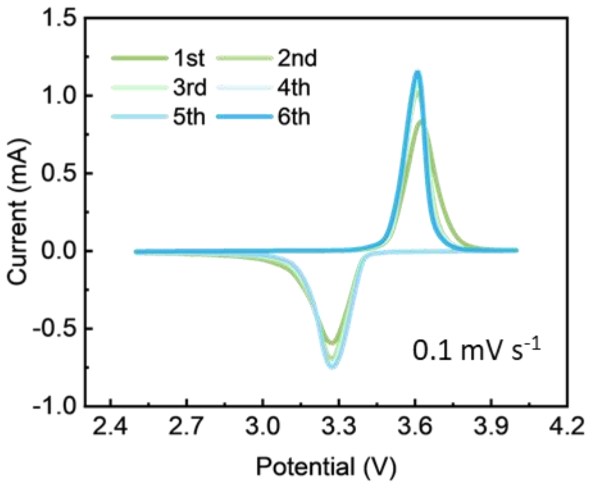

Figure S28. CV curves of Li|PVC-24h|$LiFePO_4$ cell at different cycles.

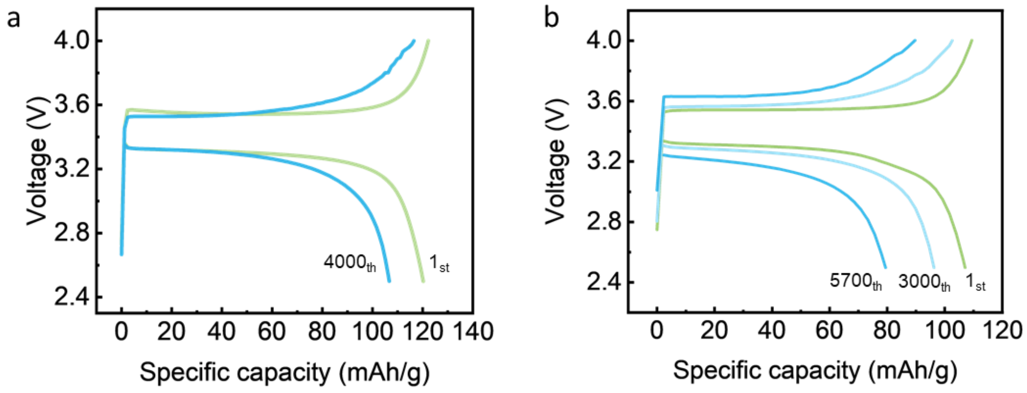

Figure S29. Charge-discharge curves of Li|PVC-24h|$LiFePO_4$ batteries at (a) 5 C and (b) 10 C at 30 ℃.

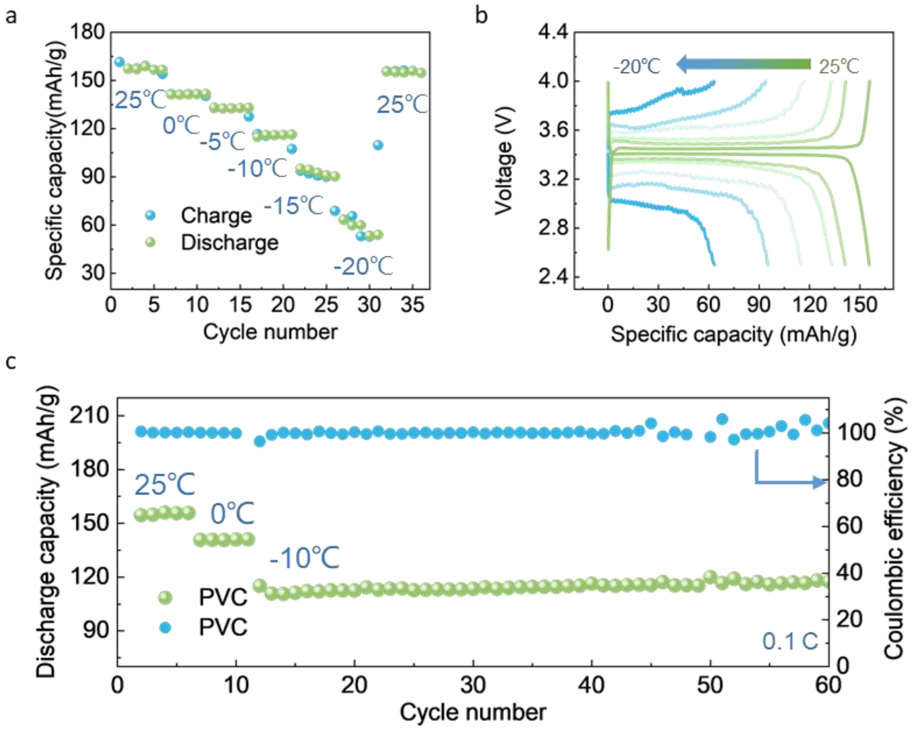

Figure S30. (a) Rate performance, (b) corresponding charge-discharge curves and (c) cycling performance of Li|PVC-24h|LiFePO$_4$ full cells at 0.1 C under different temperatures.

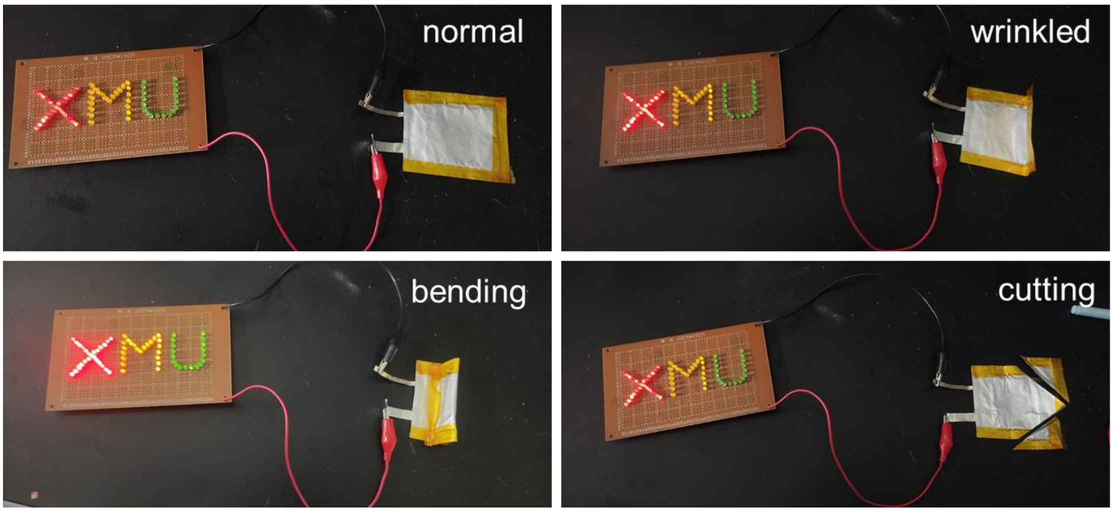

Figure S31. The optical photographs of the assembled Li|PVC-24h|LiFePO$_4$ pouch cells that light up the logo at the normal, wrinkled, cutting, and bending states.

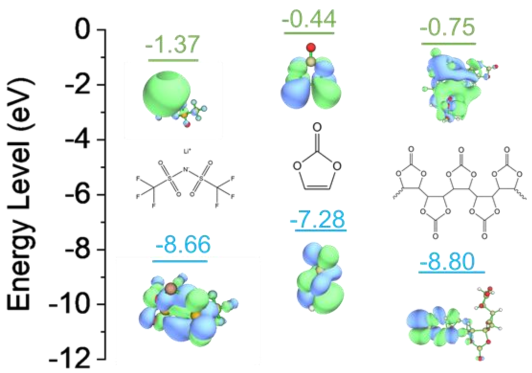

Figure S32. HOMO and LUMO energy levels of LiTFSI, VC and PVC, the optimized geometry are provided in Supplementary Data 5.

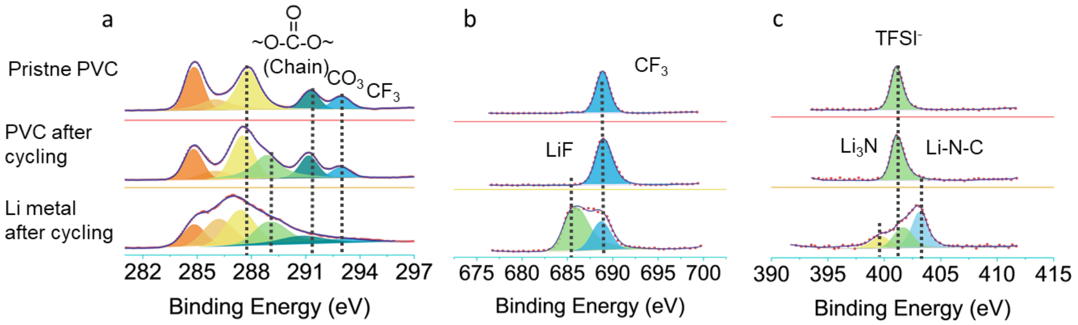

Figure S33. The (a) C 1s, (b) F 1s, and (c) N 1s XPS spectra of PVC-24h electrolyte and lithium metal surface before and after 200 cycles at 1 C in Li|PVC-24h|$LiFePO_4$ cells.

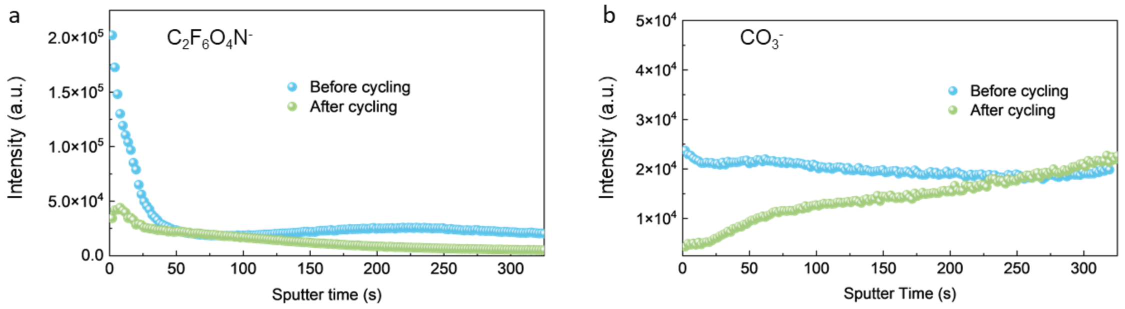

Figure S34. The intensity evolution of (a) $C_2F_6S_2O_4N^-$ and (b) CO_3^- ion fragments in PVC electrolyte with sputtering time before and after cycling.

Supplementary Tables

Table S1. The volumes of different anions[1, 2]

**Salt**	LiTFSI	LiDFOB	$LiClO_4$	$LiPF_6$
V_a **(Å^3)**	147	99	55	69

Table S2. Comparison of the electrochemical performance

	Specific capacity at 1 C (mAh g^{-1})	Cycle number	C-Rate performance	Temperature (℃)	Major component	Electrode materials	Loading density (mg cm^{-2})
SPE1[3]	147	600	1	25	PVC-SN-LiTFSI	LFP||Li	2.5
SPE2[4]	112	900	1	40	P(VEC-EEA)-LiTFSI	LFP||Li	1.5
SPE3[5]	116.5	500	0.5	60	PDOL-LiTFSI	LFP||Li	0.8~1.8
SPE4[6]	147	600	2	25	PDOL-$LiPF_6$	LFP||Li	1.2
SPE5[7]	135	1000	1	30	PEG-TMDI-LiTFSI	LFP||Li	1.5
SPE6[8]	147.6	1000	1	25	P(FN-MMA)-LiDFOB	LFP||Li	1.8
SPE7[9]	148	200	2	25	P(VC-PFS)-LiTFSI	LFP||Li	2~4
SPE8[10]	140	100	1	25	PVEC-LiTFSI	LFP||Li	3~5
This work	151.3	1000	1	30	PVC-LiTFSI	LFP||Li	3
This work	122	4000	5	30	PVC-LiTFSI	LFP||Li	3
This work	109.4	5000	10	30	PVC-LiTFSI	LFP||Li	3

Supplementary References

1. Han H-B, *et al.* Lithium bis(fluorosulfonyl)imide (LiFSI) as conducting salt for nonaqueous liquid electrolytes for lithium-ion batteries: Physicochemical and electrochemical properties. *J Power Sources* **196**, 3623-3632 (2011).

2. Han S-D, *et al.* Electrolyte Solvation and Ionic Association. *J Electrochem Soc* **160**, A2100-A2110 (2013).

3. Peng H, *et al.* Molecular Design for In-Situ Polymerized Solid Polymer Electrolytes Enabling Stable Cycling of Lithium Metal Batteries. *Adv Energy Mater* **14**, 2400428 (2024).

4. Qin S, *et al.* Separator-Free In Situ Dual-Curing Solid Polymer Electrolytes with Enhanced Interfacial Contact for Achieving Ultrastable Lithium-Metal Batteries. *Adv Energy Mater* **13**, 2301470 (2023).

5. Xu H, Zhang J, Zhang H, Long J, Xu L, Mai L. In Situ Topological Interphases Boosting Stable Solid-State Lithium Metal Batteries. *Adv Energy Mater* **13**, 2204411 (2023).

6. Mu K, *et al.* Hybrid Crosslinked Solid Polymer Electrolyte via In-Situ Solidification Enables High-Performance Solid-State Lithium Metal Batteries. *Adv Mater* **35**, 2304686 (2023).

7. Jiang Y, Song Y, Chen X, Wang H, Deng L, Yang G. In situ formed self-healable quasi-solid hybrid electrolyte network coupled with eutectic mixture towards ultra-long cycle life lithium metal batteries. *Energy Storage Mater* **52**, 514-523 (2022).

8. Sun Q, *et al.* Fumaronitrile-fixed in-situ gel polymer electrolyte balancing high safety and superior electrochemical performance for Li metal batteries. *Energy Storage Mater* **44**, 537-546 (2022).

9. Li J, Zhang H, Cui Y, Da H, Cai Y, Zhang S. Constructing interfacial gradient layers and enhancing lithium salt dissolution kinetics for high-rate solid-state batteries. *Nano Energy* **102**, 107716 (2022).

10. Lin Z, *et al.* A wide-temperature superior ionic conductive polymer electrolyte for lithium metal battery. *Nano Energy* **73**, 104786 (2020).
