# Supplementary material for: Tailoring electrolyte phase separation for high-rate solid-state lithium metal batteries
Source: Nat Commun. 2026 Jun 8;17:7310. doi: 10.1038/s41467-026-74094-w (PMC13402737; doi:10.1038/s41467-026-74094-w)
Supplement: Supplementary file 2 — Description of Additional Supplementary Files [file 41467_2026_74094_MOESM2_ESM.pdf]

Description of Additional Supplementary Files

File Name: Supplementary Data 1
Description:

The binding energies of different lithium salts with VC and PVC molecules were calculated in Figure 1c and Figure S9a.

File Name: Supplementary Data 2
Description:

The probability of electron cloud density distribution after the complexation of different ions with VC and PVC molecules were calculated in Figure 1d, Figure 4b and Figure S9b, c. The atomic valence states of PVC and VC in their original state and after complexing with lithium ions were calculated in Figure 4a.

File Name: Supplementary Data 3

Description:

The migration barriers at different positions during the migration process of lithium ions were simulated in Figure 3h.

File Name: Supplementary Data 4

Description:

Molecular dynamics simulations were conducted on the molecular structures of different phases in the PVC electrolyte, and the related mean displacements and coordination numbers were calculated in Figures 3d, e, f, g and Figure S21a, b.

File Name: Supplementary Data 5

Description:

The energy levels of VC, PVC molecules and LiTFSI were calculated in Figure S32.
