# Supplementary figures and images for: Tailoring electrolyte phase separation for high-rate solid-state lithium metal batteries
Source: Nat Commun. 2026 Jun 8;17:7310. doi: 10.1038/s41467-026-74094-w (PMC13402737; doi:10.1038/s41467-026-74094-w)

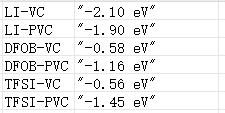

Supplement: Supplementary file 3 — Supplementary Data [file 41467_2026_74094_MOESM3_ESM.zip › Supplementary Data/Supplementary Data 1/Binding energy/119ccd37815a863166a420bb407dd89.png]

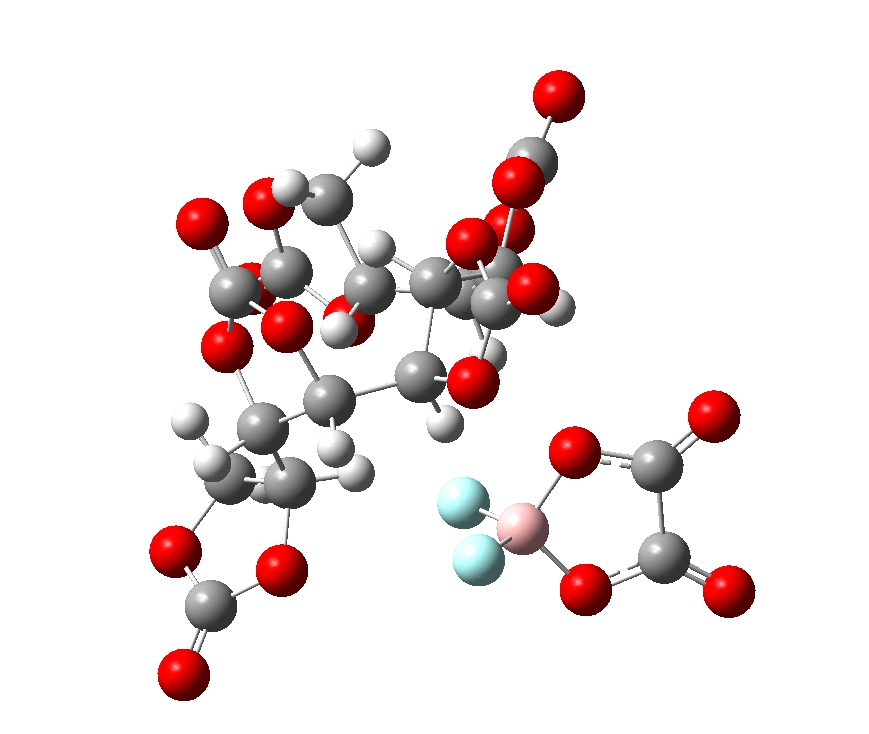

Supplement: Supplementary file 3 — Supplementary Data [file 41467_2026_74094_MOESM3_ESM.zip › Supplementary Data/Supplementary Data 1/Binding energy/DFOB-PVC.png]

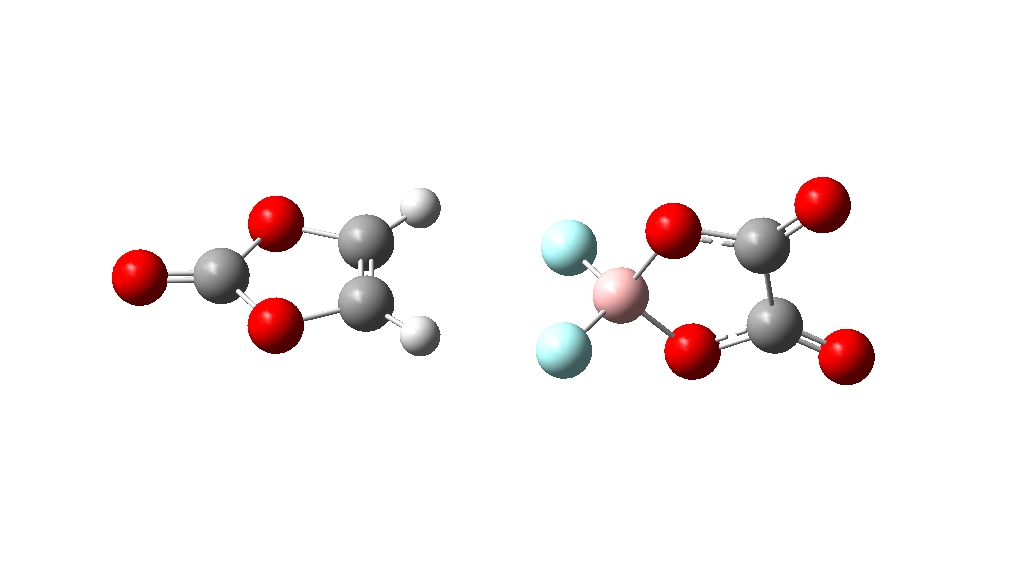

Supplement: Supplementary file 3 — Supplementary Data [file 41467_2026_74094_MOESM3_ESM.zip › Supplementary Data/Supplementary Data 1/Binding energy/DFOB-VC.png]

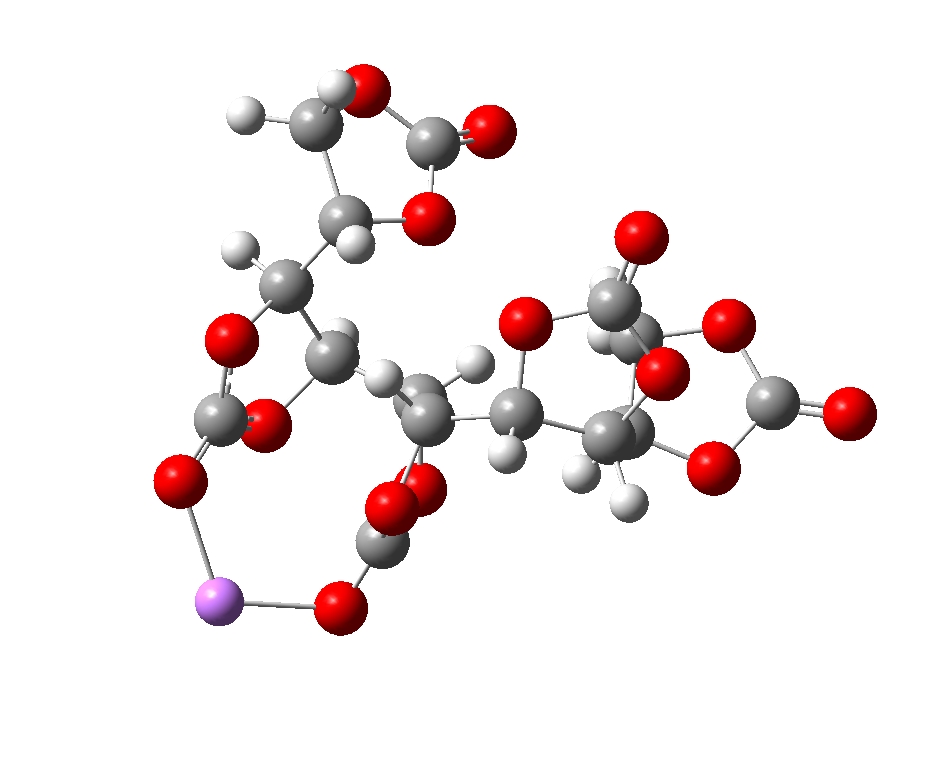

Supplement: Supplementary file 3 — Supplementary Data [file 41467_2026_74094_MOESM3_ESM.zip › Supplementary Data/Supplementary Data 1/Binding energy/LI-PVC.png]

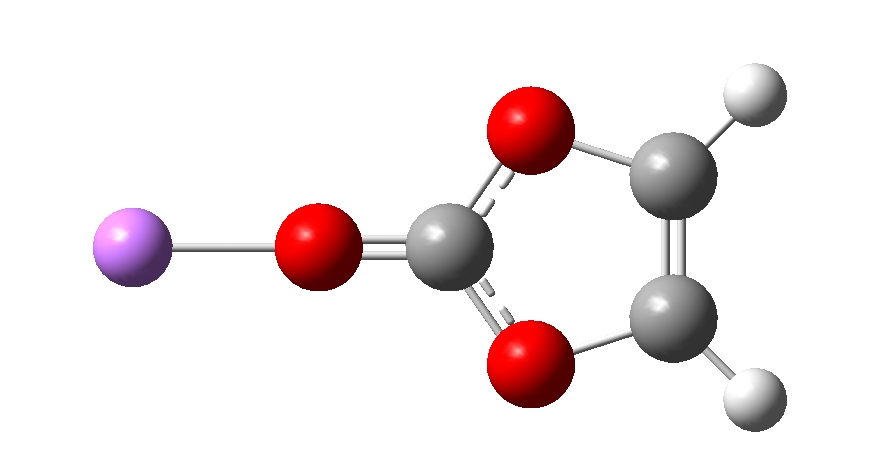

Supplement: Supplementary file 3 — Supplementary Data [file 41467_2026_74094_MOESM3_ESM.zip › Supplementary Data/Supplementary Data 1/Binding energy/LI-VC.png]

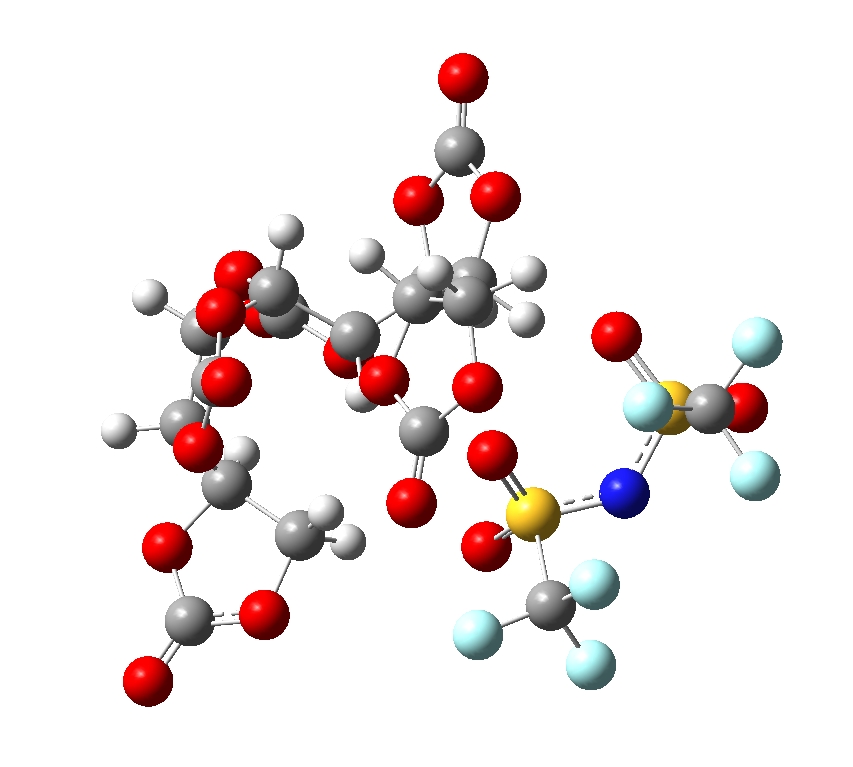

Supplement: Supplementary file 3 — Supplementary Data [file 41467_2026_74094_MOESM3_ESM.zip › Supplementary Data/Supplementary Data 1/Binding energy/TFSI-PVC.png]

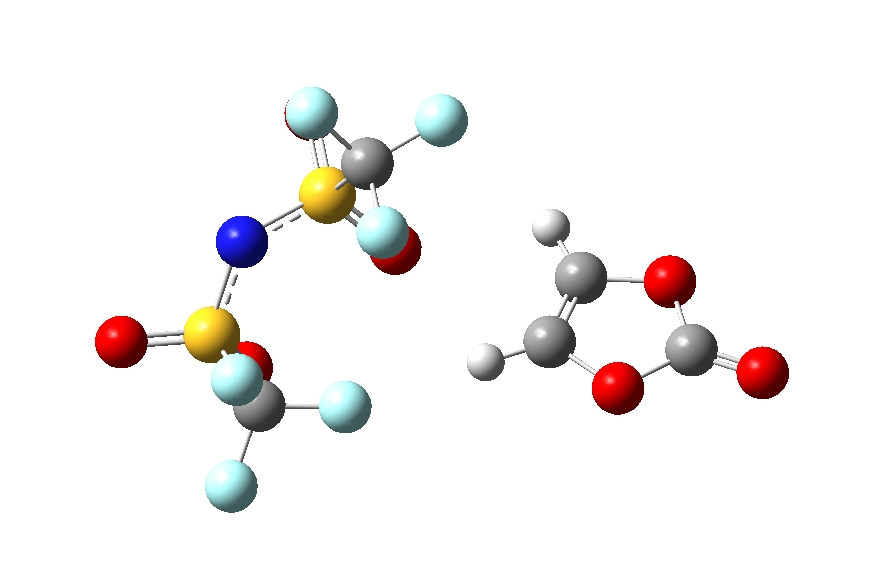

Supplement: Supplementary file 3 — Supplementary Data [file 41467_2026_74094_MOESM3_ESM.zip › Supplementary Data/Supplementary Data 1/Binding energy/TFSI-VC.png]

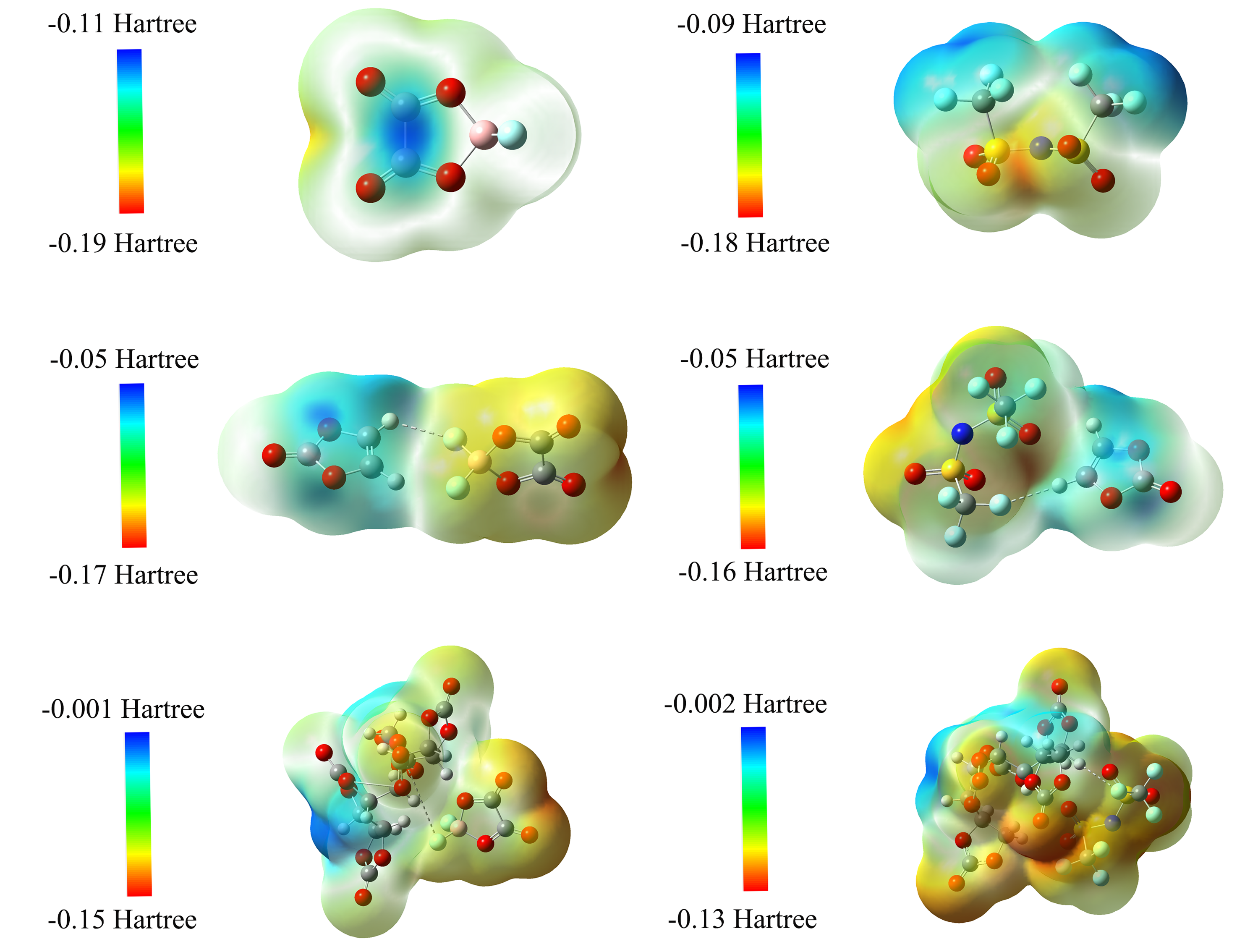

Supplement: Supplementary file 3 — Supplementary Data [file 41467_2026_74094_MOESM3_ESM.zip › Supplementary Data/Supplementary Data 2/ESP1/静电势.png]

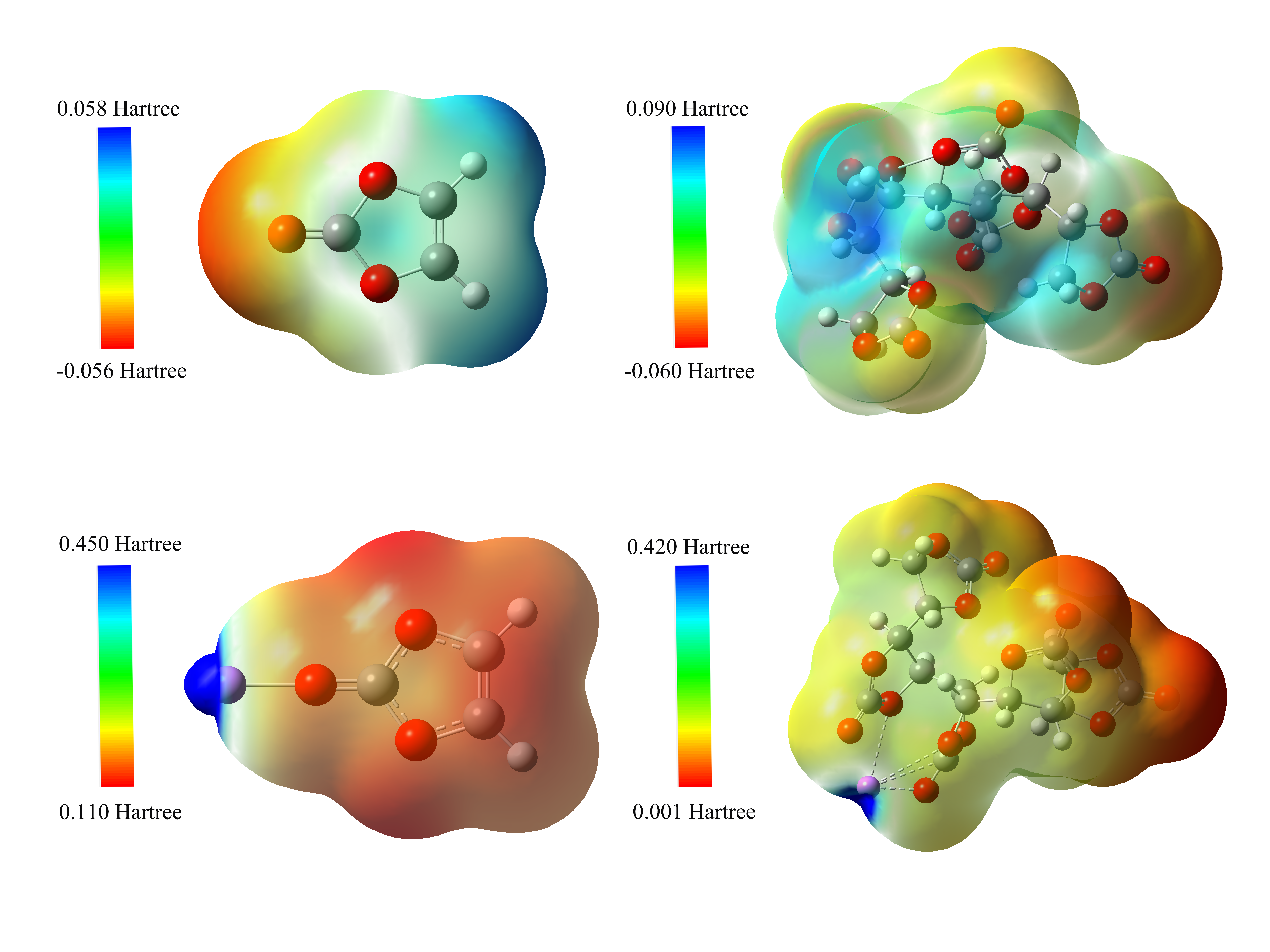

Supplement: Supplementary file 3 — Supplementary Data [file 41467_2026_74094_MOESM3_ESM.zip › Supplementary Data/Supplementary Data 2/ESP2/ESP.png]

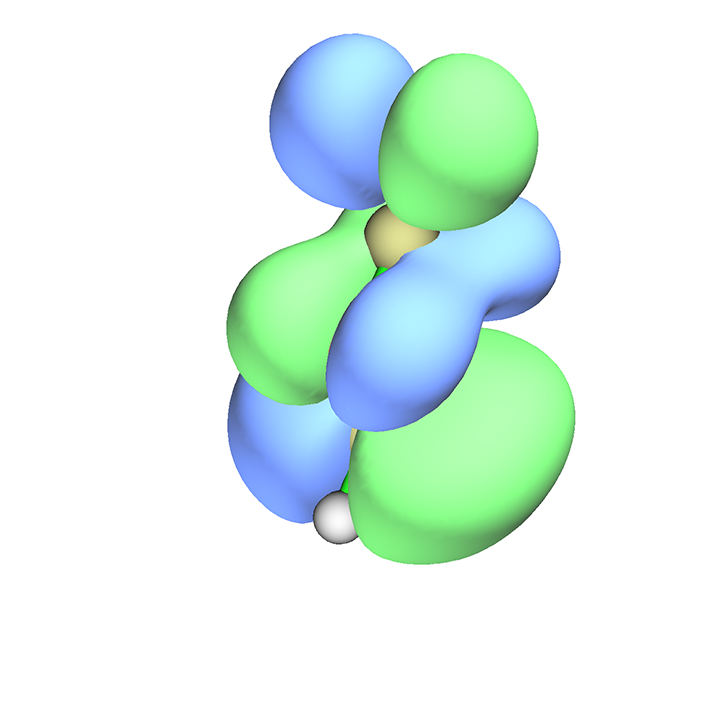

Supplement: Supplementary file 3 — Supplementary Data [file 41467_2026_74094_MOESM3_ESM.zip › Supplementary Data/Supplementary Data 5/Energy Level/danti-h(-7.28).png]

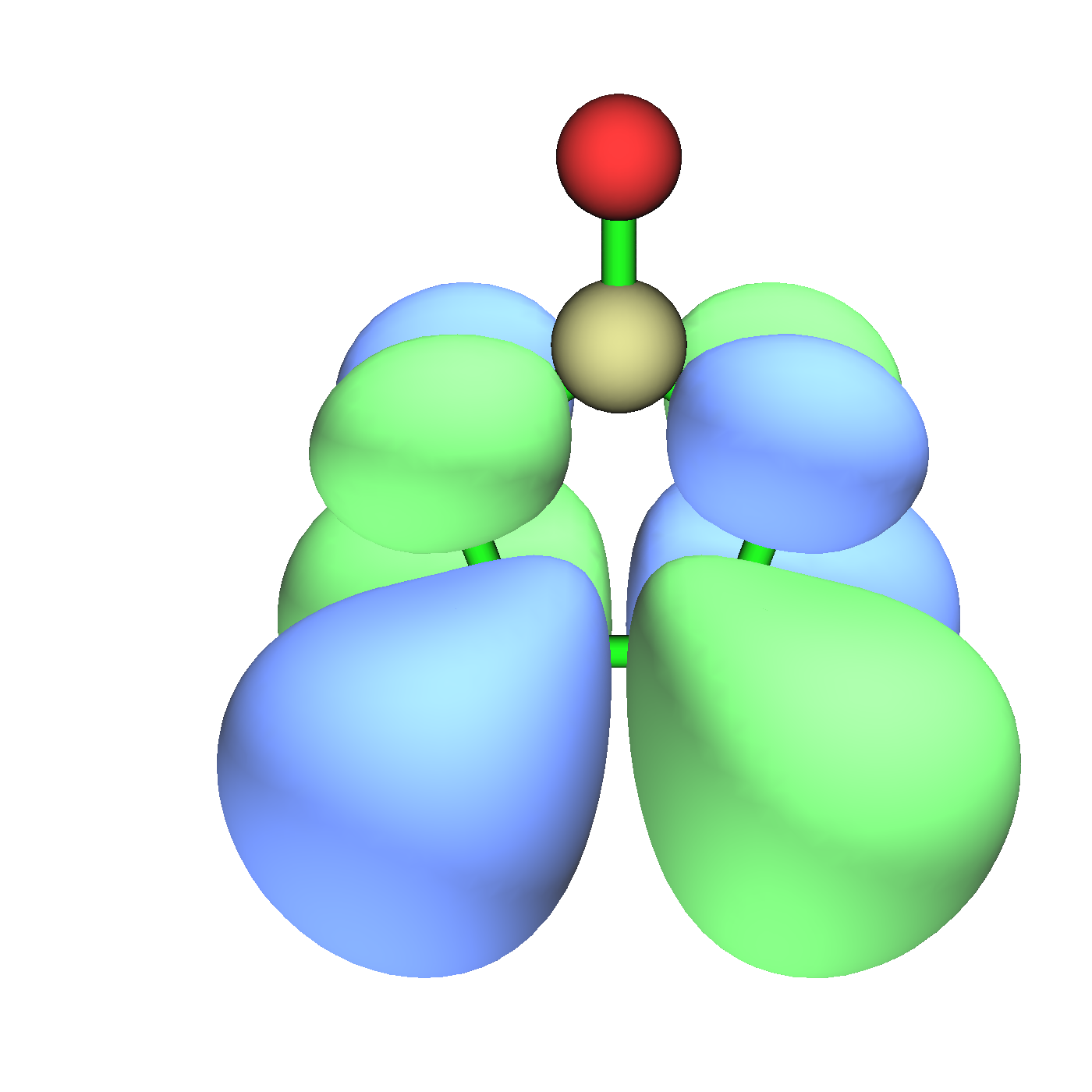

Supplement: Supplementary file 3 — Supplementary Data [file 41467_2026_74094_MOESM3_ESM.zip › Supplementary Data/Supplementary Data 5/Energy Level/danti-l(-0.44).png]

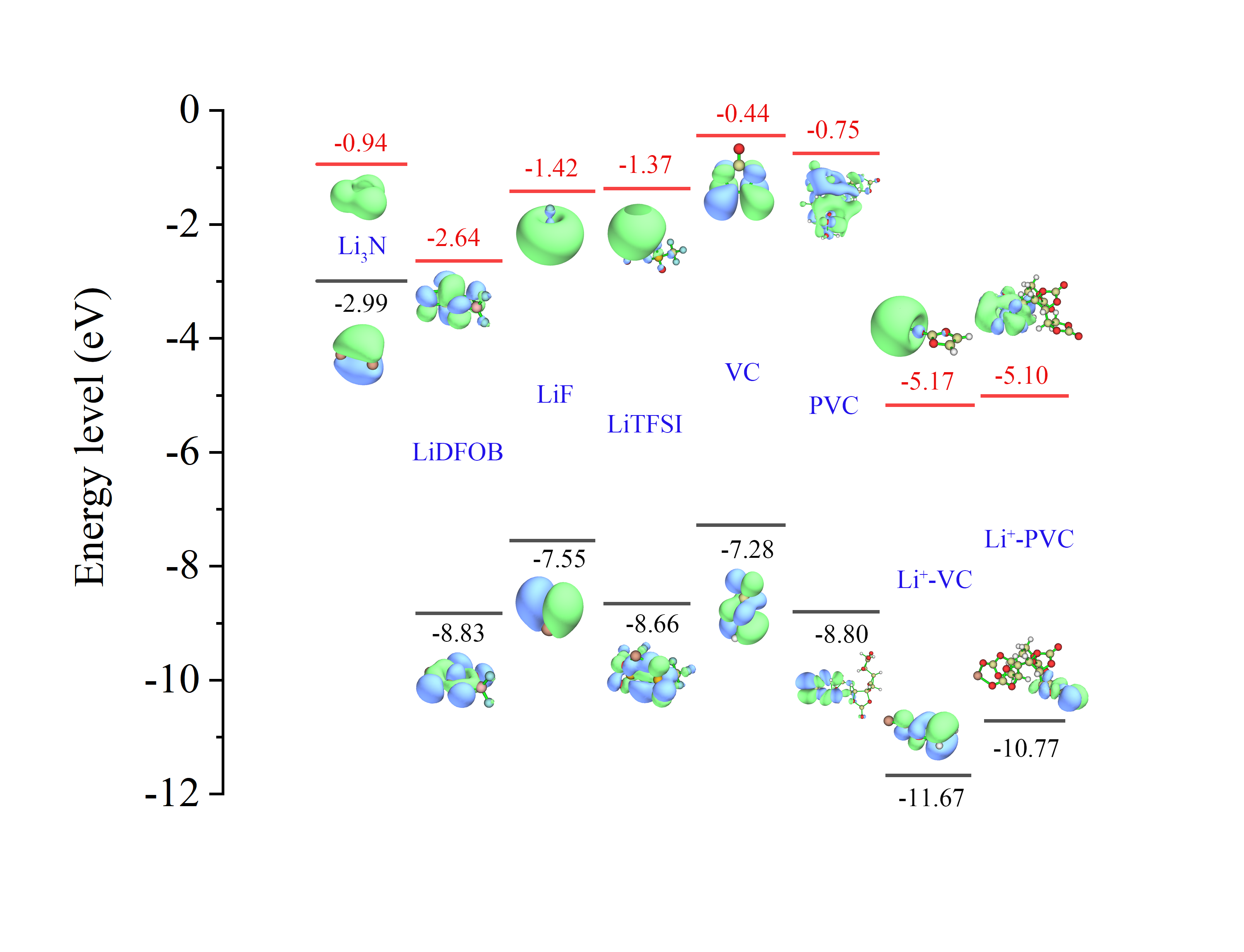

Supplement: Supplementary file 3 — Supplementary Data [file 41467_2026_74094_MOESM3_ESM.zip › Supplementary Data/Supplementary Data 5/Energy Level/Graph1.tif]

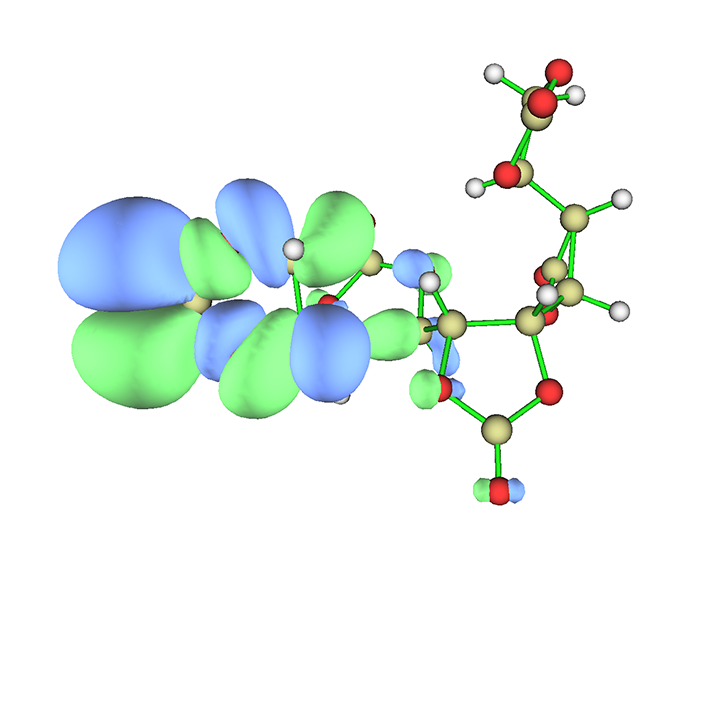

Supplement: Supplementary file 3 — Supplementary Data [file 41467_2026_74094_MOESM3_ESM.zip › Supplementary Data/Supplementary Data 5/Energy Level/juhewu-h(-8.80).png]

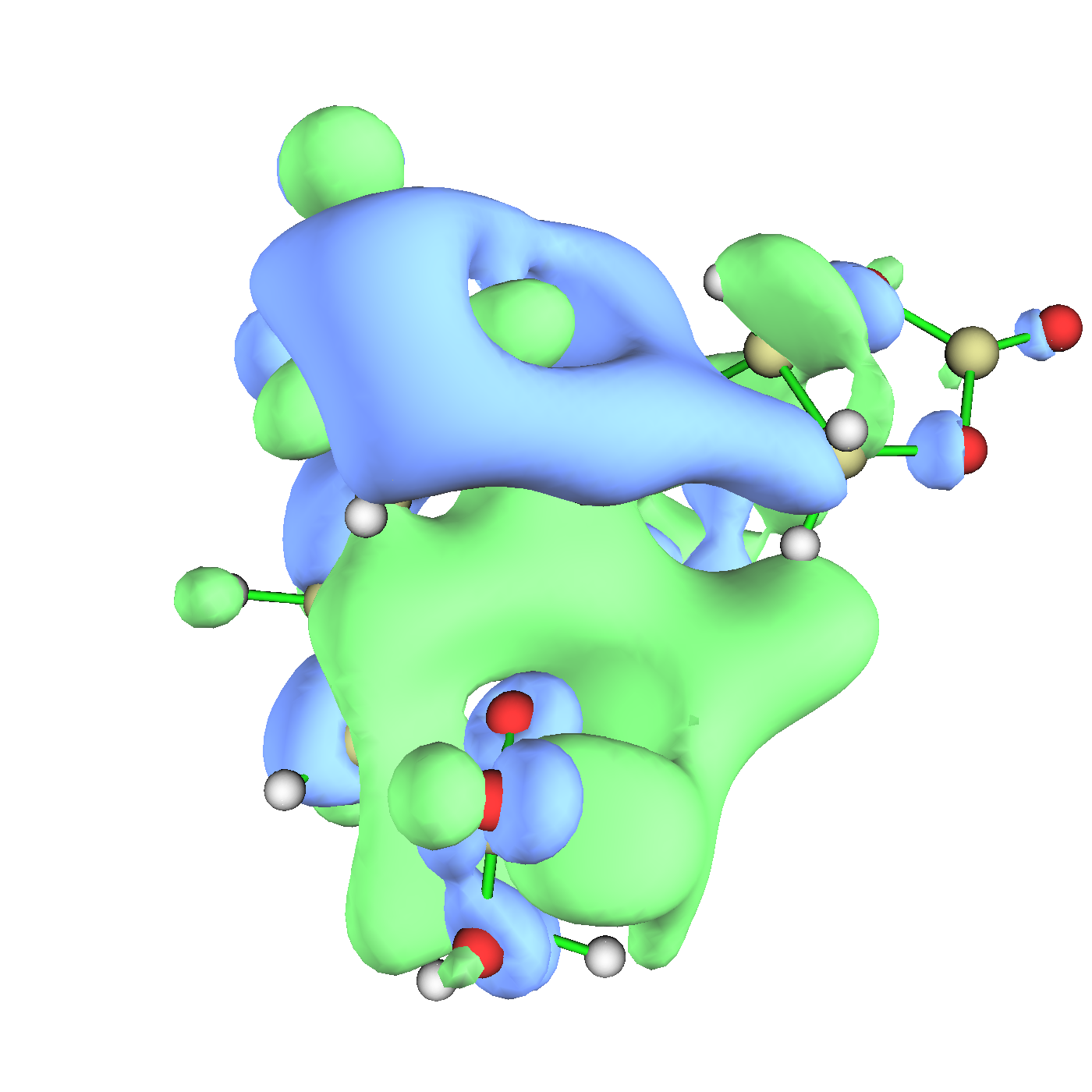

Supplement: Supplementary file 3 — Supplementary Data [file 41467_2026_74094_MOESM3_ESM.zip › Supplementary Data/Supplementary Data 5/Energy Level/juhewu-l (-0.75).png]

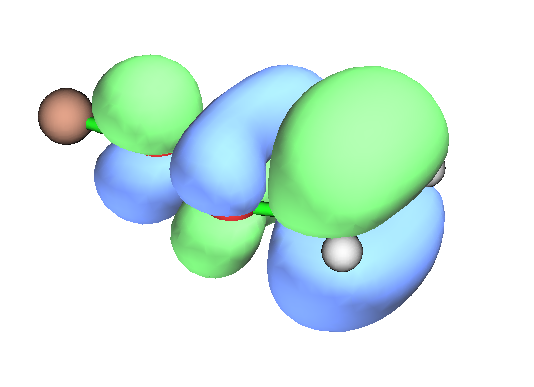

Supplement: Supplementary file 3 — Supplementary Data [file 41467_2026_74094_MOESM3_ESM.zip › Supplementary Data/Supplementary Data 5/Energy Level/li-danti-h(-11.67).png]

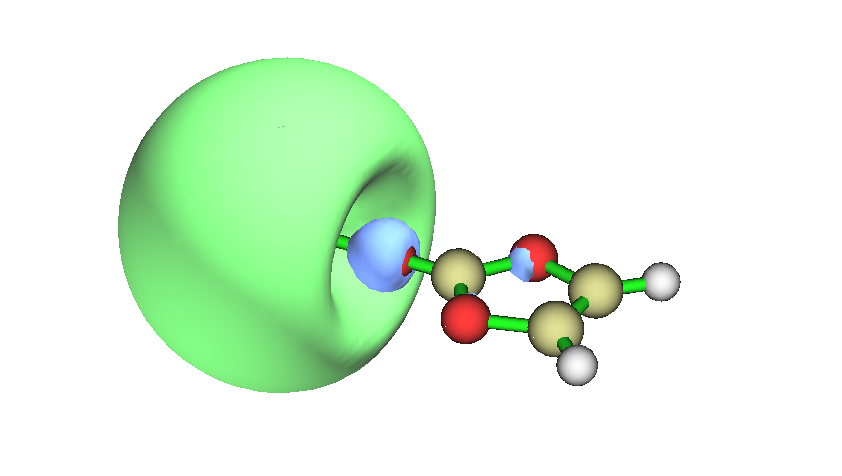

Supplement: Supplementary file 3 — Supplementary Data [file 41467_2026_74094_MOESM3_ESM.zip › Supplementary Data/Supplementary Data 5/Energy Level/li-danti-l(-5.17).png]

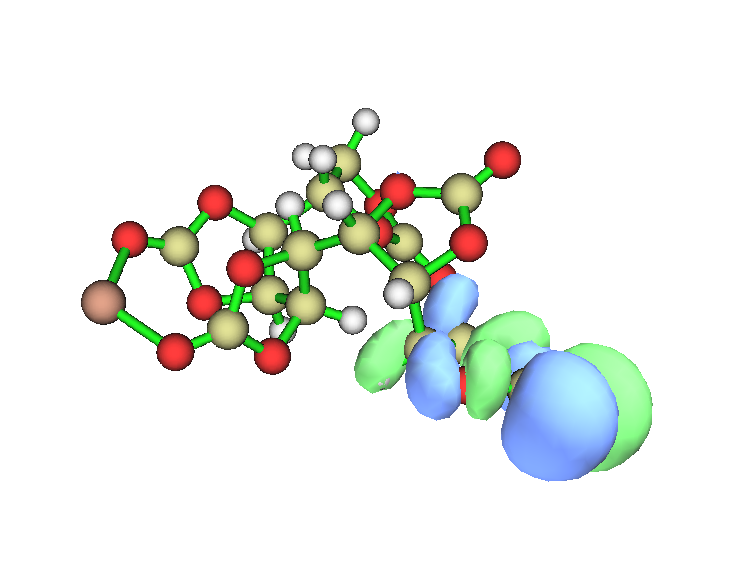

Supplement: Supplementary file 3 — Supplementary Data [file 41467_2026_74094_MOESM3_ESM.zip › Supplementary Data/Supplementary Data 5/Energy Level/li-juhewu-h(-10.77).png]

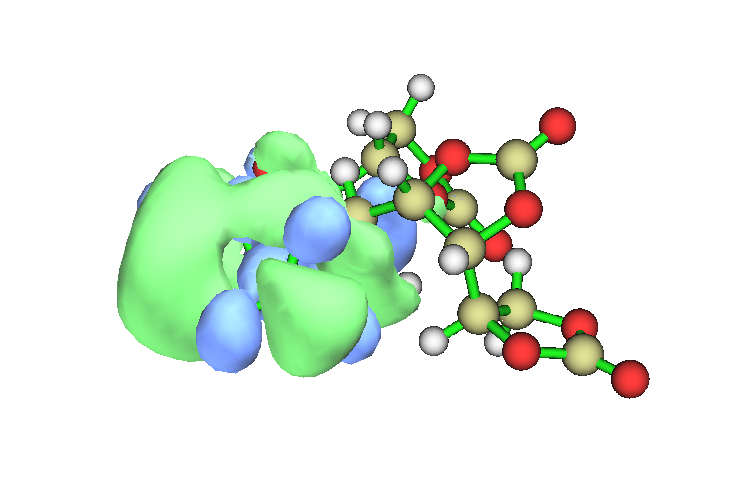

Supplement: Supplementary file 3 — Supplementary Data [file 41467_2026_74094_MOESM3_ESM.zip › Supplementary Data/Supplementary Data 5/Energy Level/li-juhewu-l(-5.10).png]

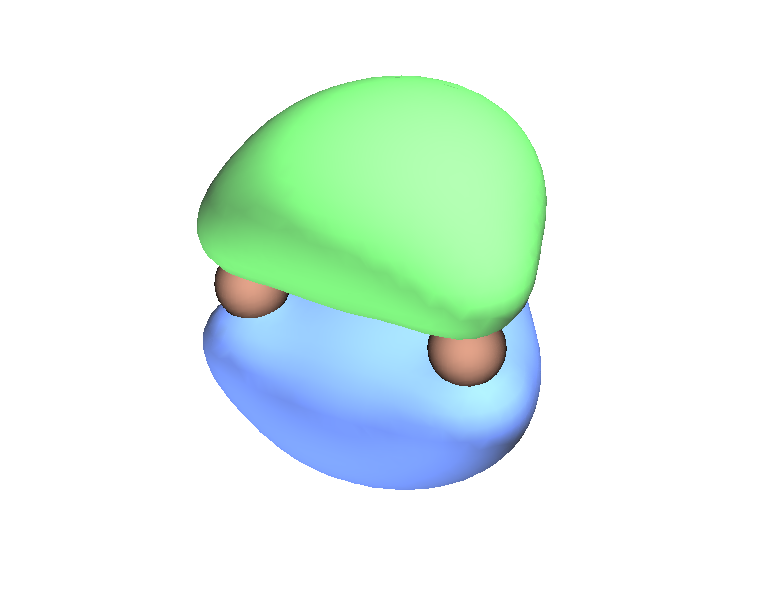

Supplement: Supplementary file 3 — Supplementary Data [file 41467_2026_74094_MOESM3_ESM.zip › Supplementary Data/Supplementary Data 5/Energy Level/li3n-h(-2.99).png]

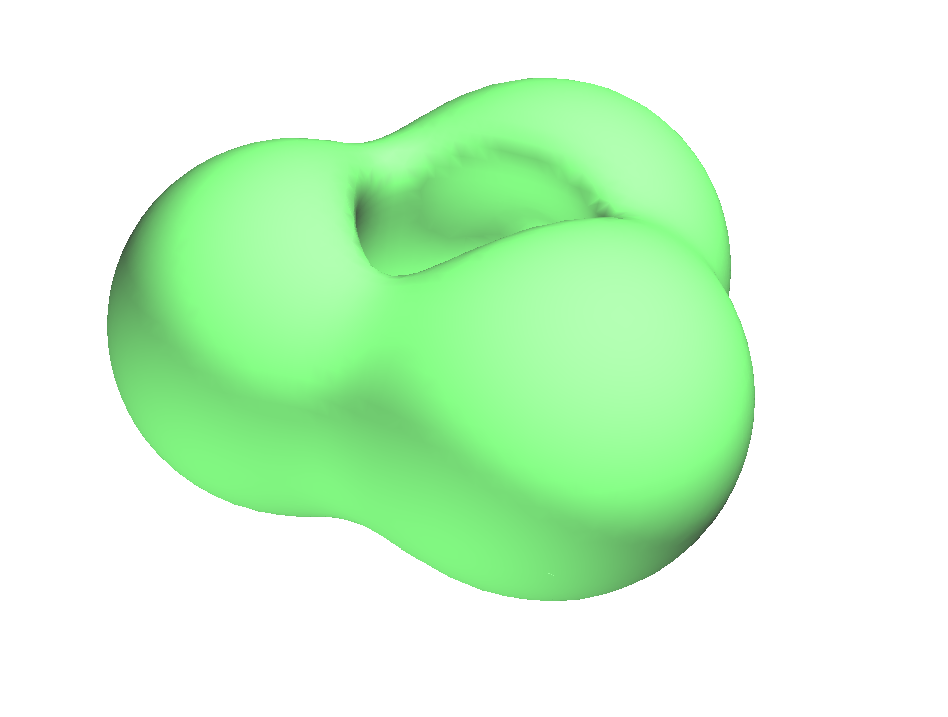

Supplement: Supplementary file 3 — Supplementary Data [file 41467_2026_74094_MOESM3_ESM.zip › Supplementary Data/Supplementary Data 5/Energy Level/li3n-l(-0.94).png]

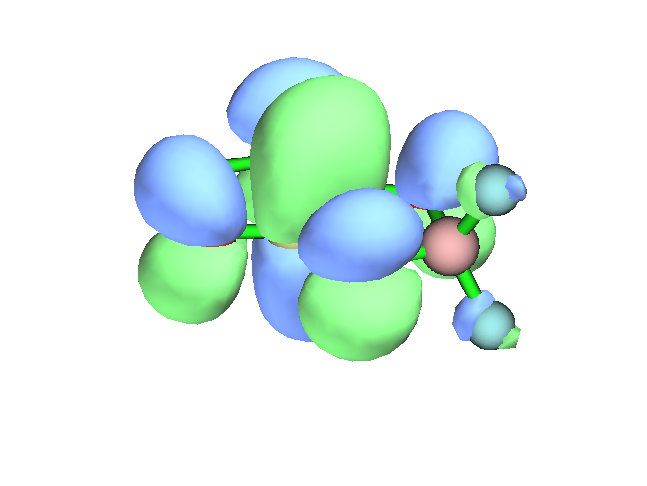

Supplement: Supplementary file 3 — Supplementary Data [file 41467_2026_74094_MOESM3_ESM.zip › Supplementary Data/Supplementary Data 5/Energy Level/LIDFOB-h(-2.64).png]

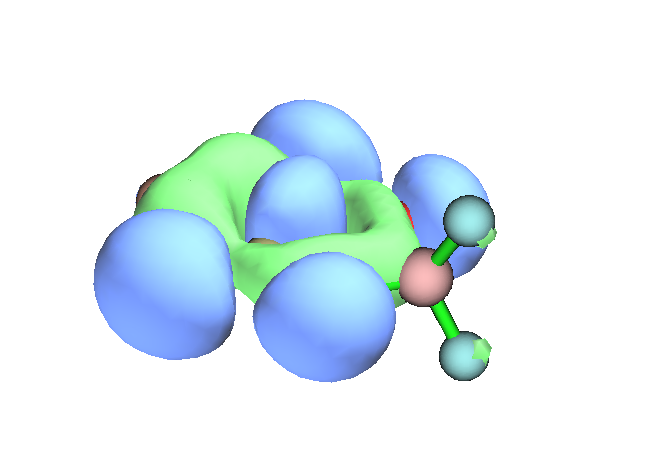

Supplement: Supplementary file 3 — Supplementary Data [file 41467_2026_74094_MOESM3_ESM.zip › Supplementary Data/Supplementary Data 5/Energy Level/LIDFOB-h(-8.83).png]

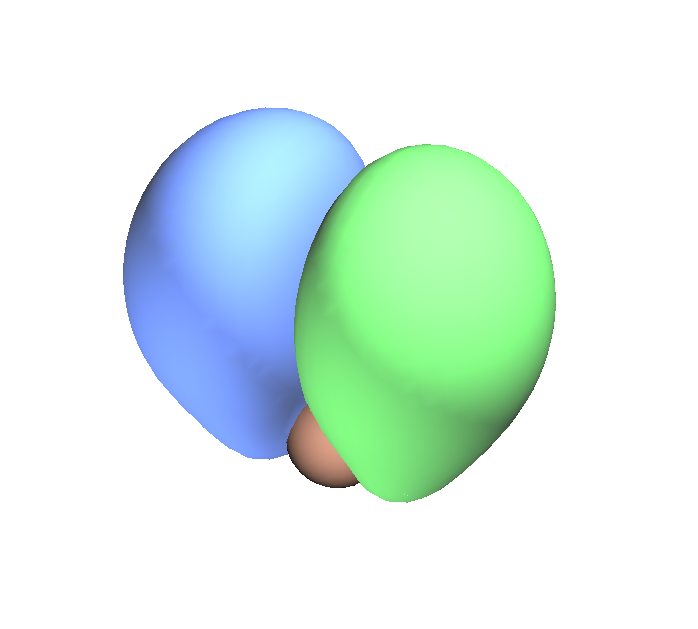

Supplement: Supplementary file 3 — Supplementary Data [file 41467_2026_74094_MOESM3_ESM.zip › Supplementary Data/Supplementary Data 5/Energy Level/LIF-h(-7.55).png]

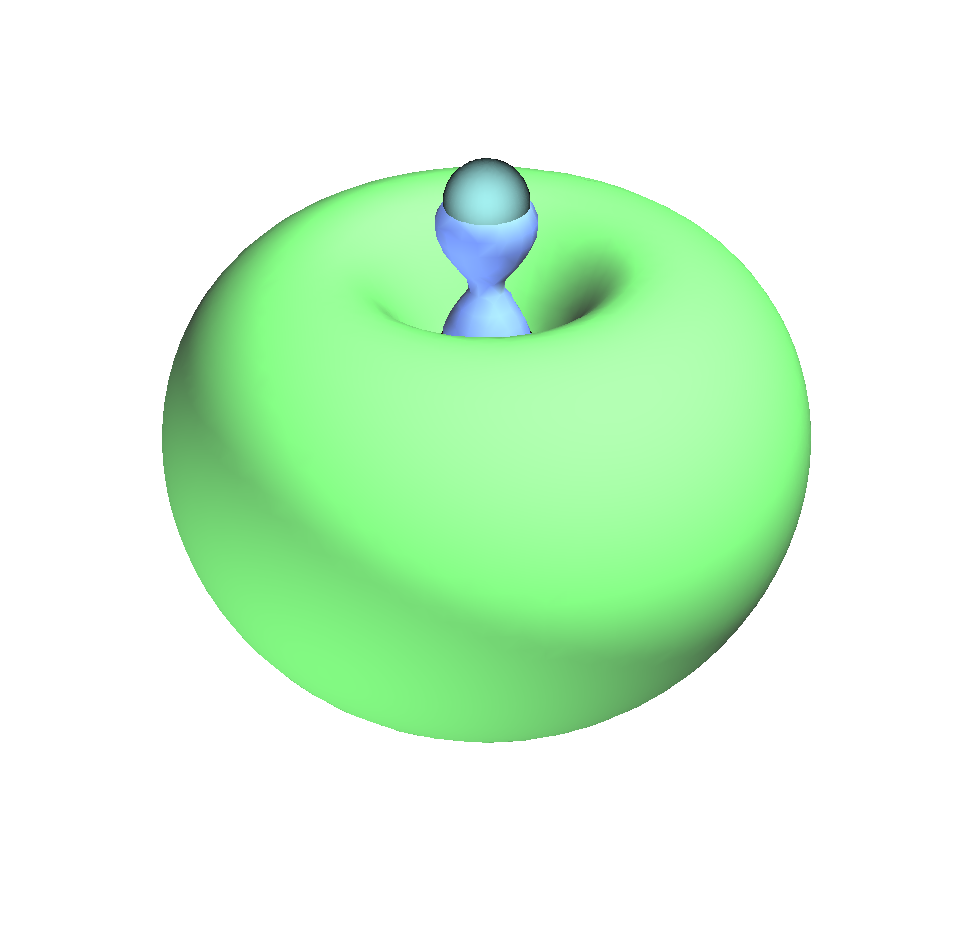

Supplement: Supplementary file 3 — Supplementary Data [file 41467_2026_74094_MOESM3_ESM.zip › Supplementary Data/Supplementary Data 5/Energy Level/LIF-l(-1.42).png]

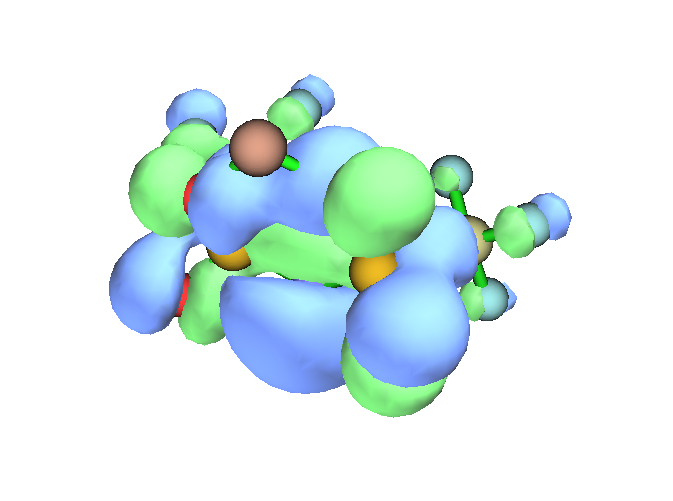

Supplement: Supplementary file 3 — Supplementary Data [file 41467_2026_74094_MOESM3_ESM.zip › Supplementary Data/Supplementary Data 5/Energy Level/LITFSI-h(-8.66).png]

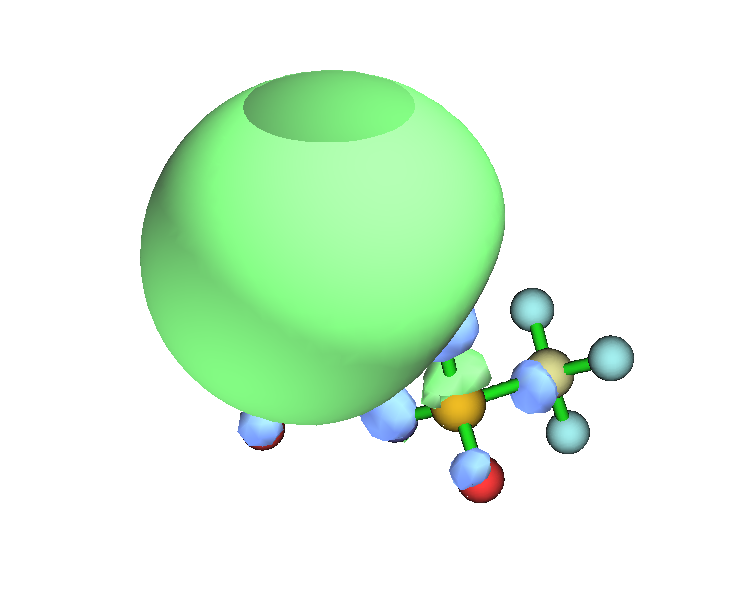

Supplement: Supplementary file 3 — Supplementary Data [file 41467_2026_74094_MOESM3_ESM.zip › Supplementary Data/Supplementary Data 5/Energy Level/LITFSI-l(-1.37).png]
