# Supplementary material for: Tailoring electrolyte phase separation for high-rate solid-state lithium metal batteries
Source: Nat Commun. 2026 Jun 8;17:7310. doi: 10.1038/s41467-026-74094-w (PMC13402737; doi:10.1038/s41467-026-74094-w)
Supplement: Supplementary file 4 — Transparent Peer Review file [file 41467_2026_74094_MOESM4_ESM.pdf]

nature portfolio

Peer Review File

Tailoring electrolyte phase separation for high-rate solid-state lithium metal batteries

Corresponding Author: Dr Jiantao Li

This file contains all reviewer reports in order by version, followed by all author rebuttals in order by version.

Version 0:

Reviewer comments:

Reviewer #1

(Remarks to the Author)
This manuscript reports a LiTFSI-mediated in-situ polymerization strategy to induce phase separation in a poly(vinylene carbonate) (PVC)-based solid polymer electrolyte using a single-solvent system. A broad range of experimental and theoretical techniques, including NMR, FTIR, SAXS, AFM, MD simulations, and electrochemical characterization, are employed, and the resulting electrolyte exhibits attractive electrochemical performance, such as relatively high ionic conductivity, a high lithium-ion transference number, and excellent cycling stability in LiFePO4//Li cells even at high C-rates. These results indicate that the system may have potential relevance for solid-state lithium metal batteries. However, despite the extensive experimental effort and the promising electrochemical performance, the manuscript suffers from fundamental weaknesses in mechanistic interpretation and internal consistency. Most critically, the central claims regarding LiTFSI-driven phase separation, dual-phase (or two-mode) Li^+ transport, and the role of polymer–anion interactions are not sufficiently supported by the presented data. Several key observations, such as strong PVC–$TFSI^-$ binding, the emergence of two "phases," enhanced ionic conductivity, increased Li^+ diffusion coefficients, and improved Li^+ transference numbers, are interpreted in ways that are internally contradictory or not uniquely justified by the experiments. Therefore, although the manuscript demonstrates significant experimental effort and reports impressive electrochemical performance, the current version does not provide a sufficiently convincing or self-consistent mechanistic understanding to support its conclusions.

Major Comments
1. In Fig. 1a and Fig. S1a, the authors state that the largest binding energy between $TFSI^-$ and PVC implies the strongest electrostatic interaction and therefore poor salt dissociation. However, if PVC binds strongly to $TFSI^-$, PVC could also compete with Li^+ for $TFSI^-$ and thereby promote LiTFSI dissociation. Please clarify the logic linking strong PVC–$TFSI^-$ binding to reduced dissociation. In addition, the manuscript argues that strong PVC–$TFSI^-$ interaction induces PVC precipitation from the VC solvent and triggers phase separation. If so, does $TFSI^-$ predominantly located in the precipitated PVC-rich phase? If $TFSI^-$ preferentially associates with PVC rather than VC, what is the mechanistic origin of this preference?

2. In the 7Li NMR spectra, the authors report the appearance of an additional peak shifting downfield from –0.3 ppm to –0.1 ppm. This observation is interpreted as evidence for two distinct Li^+ solvation environments: one dominated by the precursor and the other by PVC. The downfield shift associated with the polymer-coordinated Li^+ is reasonably explained by the reduced C=O–Li^+ interactions, which would weaken Li^+ coordination and consequently shift the resonance downfield. While this explanation is plausible for Li^+, it remains less convincing for $TFSI^-$. Unlike Li^+, $TFSI^-$ carries an overall negative charge, and its interactions with C=O groups are not straightforward. It is therefore unclear how $TFSI^-$ is proposed to interact specifically with PVC. Moreover, the rationale for why this interaction, with PVC rather than with the solvent VC, would dominate is not sufficiently addressed in the manuscript. Although MD simulations presented in the study suggest the existence of such interactions, a more detailed mechanistic explanation of the driving force behind the $TFSI^-$–PVC association would strengthen the authors' interpretation.

3. The phase evolution described in Fig. 1e,f is interesting and appears different from conventional PIPS. However, it remains unclear whether the observed "two phases" represent genuine phase separation or simply incomplete polymerization/low conversion with a substantial fraction of residual monomer/oligomer. In particular, the text (lines 131–136) states that "in the later stage of polymerization, the polymer phase adsorbs the remaining precursor internally," and the schematic shows an oligomer-rich phase surrounding polymer-rich domains. This may also be interpreted as a natural

consequence of partial conversion, where unreacted/oligomeric species remain around polymerized regions, rather than thermodynamically driven phase separation. Please more clearly distinguish the proposed phase separation mechanism from a low-conversion state. Relatedly, the high ionic conductivity in a nominal single-solvent system may arise from a significant fraction of mobile, liquid-like residual oligomers due to incomplete polymerization, rather than from a beneficial phase separation mechanism, this possibility should be discussed.

4. Fig. S6 presents FTIR spectra before and after polymerization, and the changes in the –C=O and –C–O–C regions are used to support the successful synthesis of PVC. However, VC polymerization is not a ring-opening polymerization. Therefore, the cyclic carbonate carbonyl and alkoxy oxygen functional groups remain present and their FTIR bands are not expected to disappear. Please clarify what specific FTIR changes are diagnostic of polymerization here. Similarly, in Fig. S7b, the reason for the change in the VC, –C=O signal requires explanation. Along the same line, the conversion in Fig. 2g is calculated by integrating 13C NMR peaks at 153.8 ppm and 152.3 ppm, which appear to correspond to carbonyl carbons and may not change dramatically upon polymerization. Why was conversion not quantified using peaks directly associated with the C=C group, which should provide a clearer before/after distinction?

5. In Fig. S7a, the vinyl C=C-related peak is labeled as “a” in the VC, 1H NMR spectrum, yet a similarly strong “a” peak remains in the PVC, 1H NMR spectrum. Likewise, in the 13C NMR spectrum, the C=C-related peak (“b”) also appears to remain at high intensity after polymerization. This suggests that a substantial fraction of VC may remain unpolymerized. And the polymerization conversion determined by NMR is only 63%, with this degree of conversion, can the material really be regarded as a solid electrolyte? If so, the improved electrochemical performance may be strongly influenced by the presence of liquid-like unreacted/oligomeric components.

6. In Fig. S10b, the manuscript interprets the upfield shift in 7Li NMR with increasing polymerization time as a decrease in Li^+–anion coordination number. However, an upfield shift corresponds to increased shielding. Please provide a more rigorous justification for why increased shielding should be interpreted as reduced Li^+–$TFSI^-$ coordination in this system.

7. In Fig.2h,i the authors claim that longer polymerization time shifts SAXS features toward higher q-values, indicating a more ordered/compact structure and a deeper degree of phase separation. If the phase-separated morphology becomes more ordered, one might expect more uniform Li^+ transport pathways. However, Fig. 3a shows ionic conductivity decreasing with longer polymerization time, while Fig. S19c shows the Li diffusion coefficient increasing with increasing phase separation degree, which appears inconsistent with the conductivity trend. Since the manuscript (lines 228–230) states that ionic conductivity is directly related to phase separation degree and oligomer-phase content, please clarify the relationship among SAXS-derived structural evolution, oligomer content, Li diffusion, and bulk ionic conductivity, and please provide a consistent mechanistic explanation that accounts for these trends. Additionally, the authors attribute improved low-temperature conductivity to increased crystallinity of high-molecular-weight polymer phases (PVC-48h vs PVC-12h), whereas higher crystallinity is generally expected to hinder ion transport and amorphous domains are typically more favorable. Please clarify why crystallinity would enhance conductivity in this system and provide supporting evidence.

8. The Arrhenius plot in Figure 3b shows that the data points do not strictly adhere to the linear fit, indicating that the chosen model may not accurately represent the temperature dependence of the ionic conductivity. Please re-examine the data fitting to ensure the determined activation energy is accurately calculated based on the data points. And, The X-axis label in Figure 3b contains a spelling error ("Temproture").

9. In lines 280–282, the authors state that weakened Li^+–$TFSI^-$ interaction suppresses anion mobility and consequently improves the Li^+ transference number. Please clarify why weakening Li^+–$TFSI^-$ interaction would directly lead to suppressed anion mobility. Even if Li^+–$TFSI^-$ association is weakened, $TFSI^-$ could still dissociate and migrate independently, potentially contributing to concentration polarization. A more detailed mechanistic explanation and supporting evidence are needed to justify this statement.

10. Conventionally, anion-derived inorganic SEI components are understood to originate from $TFSI^-$ species that reside within the primary solvation structure of Li^+—that is, from aggregated Li^+–anion configurations in which the anion is positioned in close proximity to the cation. In contrast, the present study argues that anions located in the outer shell, spatially separated from Li^+, also contribute to the formation of inorganic SEI. Supporting this claim, Raman spectroscopy indeed reveals an increased population of “free” anions upon polymerization. While this interpretation offers a potentially valuable new perspective on anion-derived SEI formation, it also raises a conceptual inconsistency. According to the authors’ own mechanism, VC polymerization drives $TFSI^-$ to interact with PVC, leading to precipitation of polymer-rich granular domains. The manuscript further attributes the enhanced tLi+ to the immobilization of anions within these polymeric particles. If these anions are effectively confined within the PVC-rich phase, their ability to freely diffuse and participate in inorganic SEI formation at the electrode interface becomes questionable. In other words, the proposed immobilization mechanism appears difficult to reconcile with the simultaneous claim that these same anions actively form an inorganic SEI layer. A clearer mechanistic rationale or additional supporting evidence would be necessary to resolve this discrepancy.

Minor Comments
1. The ionic conductivity reported in the abstract (0.92 mS cm^{-1}) corresponds to PVC-12h, whereas PVC-24h is the main focus. This should be corrected for consistency.
2. In Fig. 1f, “oligmer” should be corrected to “oligomer.”
3. Line 294: Please verify the correct figure reference (likely Fig. S20e instead of S18e).
4. The diffusion coefficient is shown in Fig. S19b, not S19c.
5. Line 471: “Preparation of electrodes” should be corrected to “Preparation of electrolytes.”

Reviewer #2

(Remarks to the Author)
The authors presents a new phase separation strategy to tailor the structure of Poly (Vinylene Carbonate) (PVC)-based electrolyte by lithium salts in a single-solvent, which is quite different with traditional polymerization induced phase separation process that inevitably requires the use of additional phases. More importantly, the precisely regulated separation degree by adjusting the polymerization time ensures tight interface contact between SPEs and electrode materials. Therefore, the assembled batteries exhibits high discharge capacity and superior cycling stability when cycled at a large current density of 5 C and 10 C under room temperature. The results benefit to promote the development of polymer lithium batteries. Overall, the experimental design is novel, and the findings are robust and interesting. I recommend the publication of the work after minor revisions.
1. Regarding the single-solvent phase separation behavior triggered by anions, during the process of regulating the degree of different phase separations in the electrolyte by lithium salts, does the state of the lithium salt anions change?
2. Whether the crystallinity of polymer electrolytes with different degrees of phase separation also has a significant impact on the carrier transport performance of the electrolyte should be discussed in the manuscript.
3. Details of the experiment is essential for the readers, such as the details of preparation for the test samples as well as the testing environment.
4. The assembled batteries exhibit excellent rate performance and cycle stability, but the intrinsic relationship between the performance and the separation regulation of the electrolyte needs to be discussed in detail.
5. As mentioned above, the phase separation strategy reported in this work is quite different with the traditional polymerization induced phase separation process. The advantages of such a single-solvent strategy should be discussed more elaborately as this is the core innovation of this work.
6. Some language polishing and formatting adjustments are required for consistency (e.g., There is a formatting error in the vertical axis of Figure 2i in the manuscript).

Reviewer #3

(Remarks to the Author)
The manuscript "Tailoring electrolyte phase separation for high-rate solid-state lithium metal batteries" presents an innovative in-situ polymerization strategy using a single lithium salt to induce controllable phase separation in a single-solvent system, constructing a high-performance dual-phase solid polymer electrolyte (SPE) for high-rate lithium metal batteries. The study combines theoretical calculations, multiple characterization techniques (XPS, NMR, Raman), microstructural characterizations (SEM, TEM, AFM), and electrochemical tests to elucidate the mechanism of salt-induced phase separation and the excellent electrochemical properties of electrolyte. The battery demonstrates remarkable cycling stability, retaining 90% capacity after 4000 cycles at 5 C. Therefore, the manuscript is considered suitable for publication in Nature Communications. However, a major revision is required before acceptance.

1. The dual-phase structure of the PVC polymer electrolyte includes polymer and oligomer phases, with a proposed Li^+ transport mechanism across both phases. However, the presence of residual VC monomer is not addressed. Please provide a detailed explanation regarding whether residual VC exists, and its contribution to ionic conductivity and transport mechanisms.
2. This study controls the dual-phase structure by varying polymerization time and identifies 24 h as the optimal polymerization time. However, the ionic conductivity reported in the Abstract (0.92 mS cm^{-1}) corresponds to PVC-12h, not PVC-24h. Furthermore, it is unclear whether polymerization is fully terminated after 24 h. If polymerization stops, please explain how the stop is controlled. If it does not stop, could further polymerization in a sealed cell lead to continued changes in electrolyte structure and a decrease in ionic conductivity over time?
3. The PVC-24h electrolyte shows excellent long-term cycling performance at high rates (1C, 5C, 10C for over 1000 cycles). However, the interfacial impedance evolution is only provided for up to 200 cycles (Fig. 6f). To better demonstrate interfacial stability during extended cycling, please supplement EIS data after long cycle numbers (e.g., after 500, or 1000 cycles).
4. The PVC-24h electrolyte exhibits high ionic conductivity and Li^+ transference number. However, the Li|PVC-24h|Li symmetric cell shows a relatively high interfacial impedance (Fig. 4e). Please provide a reasonable explanation. Additional characterization on the Li metal/electrolyte interface evolution and rate performance tests in Li|PVC-24h|Li symmetric cells is recommended.
5. In this work, the polymer phase is the critical factor in improving the intrinsic oxidation stability of the electrolyte. Given the high oxidation stability reported (~4.5 V for PVC-24h), whether the PVC-24h electrolyte could be applied in high-voltage systems such as NCM-based batteries? A preliminary data and brief discussion on compatibility with higher-voltage cathodes (e.g., NCM811 or NCM622) would significantly strengthen the practical relevance of this work.

Reviewer #4

(Remarks to the Author)
In this manuscript, the authors describe a process to prepare poly(vinyl carbonate) (PVC) solid polymer electrolytes through a polymerization induced phase separation technique in vinylene carbonate. The resulting PVC is reported to have excellent electrochemical properties for application in solid-state lithium metal batteries. However, because this manuscript is motivated by the development of polymeric materials for solid-state lithium metal batteries, I believe it should be rejected as the authors do not convincingly show that the polymeric and/or oligomeric phase in the phase-separated PVC is primarily

responsible for the transport and the energy storage performance. Rather, the NMR, Raman, and TGA data in Figures 2f, 2g, and 2j, respectively, suggest that substantial unreacted monomer, vinylene carbonate (VC), remains. The authors report a monomer conversion as high as only 65%, meaning 35% of the system is liquid. It is likely that residual unreacted VC is predominantly responsible for lithium transport and interfacial reactivity. It will be important to elucidate the relative roles of polymer, oligomer, and unreacted monomer in the system. Moreover, I don’t think the novelty of this study warrants publication in Nature Communications, as several papers have reported on the in situ polymerization of polymer-in-liquid systems or gel electrolytes with good energy storage performance (cf. 10.1016/j.xcrp.2020.100146 , 10.1002/advs.201600377, 10.1002/aenm.202300798,).

Version 1:

Reviewer comments:

Reviewer #1

(Remarks to the Author)
The authors have thoroughly addressed this reviewer’s concerns, and the manuscript has been significantly improved as a result. I now recommend its acceptance without further revision.

Reviewer #2

(Remarks to the Author)
The revised manuscript has responded the comments accordingly. I suggest to accept it at current edition.

Reviewer #3

(Remarks to the Author)
The manuscript entitled "Tailoring electrolyte phase separation for high-rate solid-state lithium metal batteries" presents an innovative phase separation strategy using a single lithium salt to induce controllable phase separation in a single-solvent system. The article provides comprehensive explanations for the mechanism of anion-induced phase separation. Although detailed responses and a careful revision had been conducted, a major revision is still required before acceptance.
The phase-separated PVC electrolyte was annealed at 200°C for 5 hours to evaporate residual monomer. The ionic conductivity of the annealed electrolyte decreases significantly. This result strongly indicates that the residual monomer still plays an important role in ion conduction for the PVC-LiTFSI electrolyte. PVC-LiDFOB electrolyte has more residual monomer than the PVC-LiTFSI, however, it exhibits a relatively low ionic conductivity. The authors state that the PVC-LiDFOB electrolyte shows a single phase that leads to a low conductivity. The reviewer considers that the reason for the low conductivity may lies in the limited dissociation degree of LiDFOB in the electrolyte. Therefore, the contribution of the residual monomer to high ion conduction should be clarified.

Reviewer #4

(Remarks to the Author)
The authors have addressed my initial concerns, and I believe the manuscript is suitable for publication.

Version 2:

Reviewer comments:

Reviewer #3

(Remarks to the Author)
The authors have replied the rewiewer's questions and revised their manuscript, the revised manuscript can be accepted for publishing.

Open Access This Peer Review File is licensed under a Creative Commons Attribution 4.0 International License, which permits use, sharing, adaptation, distribution and reproduction in any medium or format, as long as you give appropriate credit to the original author(s) and the source, provide a link to the Creative Commons license, and indicate if changes were made.
In cases where reviewers are anonymous, credit should be given to 'Anonymous Referee' and the source.
The images or other third party material in this Peer Review File are included in the article's Creative Commons license, unless indicated otherwise in a credit line to the material. If material is not included in the article's Creative Commons license and your intended use is not permitted by statutory regulation or exceeds the permitted use, you will need to obtain permission directly from the copyright holder.
To view a copy of this license, visit https://creativecommons.org/licenses/by/4.0/

Table of content

Response to comments from the Reviewer # 1: 2
- General comment: 2
- Reviewer #1-Comment 1: 3
- Reviewer #1-Comment 2: 6
- Reviewer #1-Comment 3: 8
- Reviewer #1-Comment 4: 12
- Reviewer #1-Comment 5: 15
- Reviewer #1-Comment 6: 19
- Reviewer #1-Comment 7: 21
- Reviewer #1-Comment 8: 24
- Reviewer #1-Comment 9: 25
- Reviewer #1-Comment 10: 27
- Reviewer #1-Comment 11: 30

Response to the Reviewer #2 31
- General comment: 31
- Reviewer #2-Comment 1: 31
- Reviewer #2-Comment 2:. 33
- Reviewer #2-Comment 3: 35
- Reviewer #2-Comment 4: 35
- Reviewer #2-Comment 5: 37
- Reviewer #2-Comment 6:. 37

Response to the Reviewer #3 39
- General comment: 39
- Reviewer #3-Comment 1: 39
- Reviewer #3-Comment 2: 46
- Reviewer #3-Comment 3: 49
- Reviewer #3-Comment 4: 50
- Reviewer #3-Comment 5: 52

Response to the Reviewer #4 54
- General comment: 54
- Reviewer #4-Comment 1:. 54
- Reviewer #4-Comment 2: 60
- Reviewer #4-Comment 3: 63
- Reviewer #4-Comment 4:. 67

Responses to the Reviewers' comments and the Revision details

Manuscript ID: NCOMMS-25-94149

Title: Tailoring electrolyte phase separation for high-rate solid-state lithium metal batteries

Journal: Nature Communications

Thanks very much for the reviewers' patient review and valuable comments with a clear goal to make the manuscript better. We have carefully revised our manuscript by taking full consideration of the reviewers' comments and made targeted revisions to this work. The point-by-point responses to the reviewers' comments and the revision details are as follows:

Response to comments from the Reviewer # 1:

General comment: This manuscript reports a LiTFSI-mediated in-situ polymerization strategy to induce phase separation in a poly(vinylene carbonate) (PVC)-based solid polymer electrolyte using a single-solvent system. A broad range of experimental and theoretical techniques, including NMR, FTIR, SAXS, AFM, MD simulations, and electrochemical characterization, are employed, and the resulting electrolyte exhibits attractive electrochemical performance, such as relatively high ionic conductivity, a high lithium-ion transference number, and excellent cycling stability in $LiFePO_4$//Li cells even at high C-rates. These results indicate that the system may have potential relevance for solid-state lithium metal batteries. However, despite the extensive experimental effort and the promising electrochemical performance, the manuscript suffers from fundamental weaknesses in mechanistic interpretation and internal consistency. Most critically, the central claims regarding LiTFSI-driven phase separation, dual-phase (or two-mode) Li^+ transport, and the role of polymer–anion interactions are not sufficiently supported by the presented data. Several key observations, such as strong PVC–$TFSI^-$ binding, the emergence of two "phases" enhanced ionic conductivity, increased Li^+ diffusion coefficients, and improved Li^+ transference numbers, are interpreted in ways that are internally contradictory or not uniquely justified by the experiments. Therefore, although the manuscript demonstrates significant experimental effort and reports impressive electrochemical performance, the current version does not provide a sufficiently

convincing or self-consistent mechanistic understanding to support its conclusions.

Reviewer #1-Comment 1: In Fig. 1a and Fig. S1a, the authors state that the largest binding energy between $TFSI^-$ and PVC implies the strongest electrostatic interaction and therefore poor salt dissociation. However, if PVC binds strongly to $TFSI^-$, PVC could also compete with Li^+ for $TFSI^-$ and thereby promote LiTFSI dissociation. Please clarify the logic linking strong PVC–$TFSI^-$ binding to reduced dissociation. In addition, the manuscript argues that strong PVC–$TFSI^-$ interaction induces PVC precipitation from the VC solvent and triggers phase separation. If so, does $TFSI^-$ predominantly located in the precipitated PVC-rich phase? If $TFSI^-$ preferentially associates with PVC rather than VC, what is the mechanistic origin of this preference?

Response: We appreciate your professional comments and apologize for any possible inaccuracies in our previous expressions. We wish to clarify that the strong binding energy between $TFSI^-$ and PVC results in the formation of PVC-$TFSI^-$ complex that is more difficult to dissociate. Moreover, this strong interaction between $TFSI^-$ and PVC facilitates the dissociation of the LiTFSI.

It should be pointed out that the in-situ polymerization of VC is a homogeneous process without phase separation occurring, which indicates that the PVC synthesized through VC polymerization does not precipitate from the precursor solution. However, when LiTFSI is introduced, the white particles, being ascribed to PVC-$TFSI^-$ complex, form and continuously precipitate from the precursor solution during polymerization process (Figure S4), resulting in a phase-separated structure. TEM images (Figure 2a,b) exhibit phase-separation characteristics, similar to those observed in SEM images (Figure 1a,b). Binding energy calculations indicate that $TFSI^-$ interacts with both VC (–0.56 eV) and PVC (–1.45 eV), while the VC coordinated with $TFSI^-$ does not precipitate from the precursor solution (Figure 1c). As the PVC complexed with $TFSI^-$ continuously precipitates from the precursor solution, the newly synthesized PVC will continue to complex with the remaining $TFSI^-$ and precipitate by means of the concentration differences., which causes $TFSI^-$ to accumulate mainly in the polymer-rich phase. F-element mapping in Cryo-TEM images (Figure 2c,d) confirms that the F atoms from $TFSI^-$ in the PVC-based electrolyte are predominantly located in the high-contrast regions, which corresponds to the polymer-rich phase from selected-area electron diffraction results. It is noteworthy that not all $TFSI^-$ anions are confined in the polymer-rich phase, the lithium-ion transference number of 0.78 implies that 22% of the $TFSI^-$ anions also participate in ion transport. The relevant contents have been added to lines 121-137 of the revised manuscript.

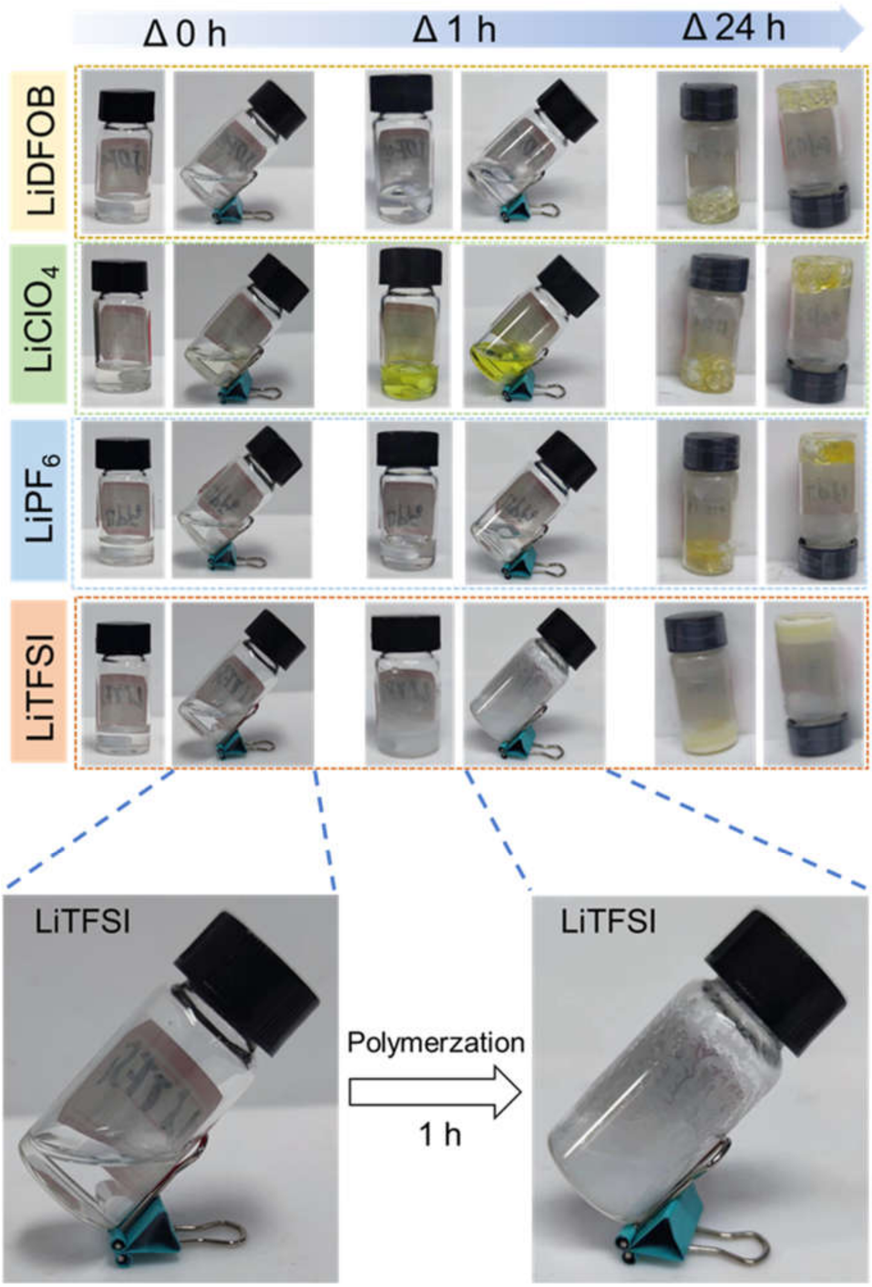

Figure S4. Optical images of PVC electrolytes regulated by different lithium salts at different polymerization time and the enlarged optical images of the precipitation of solid particles in LiTFSI-regulated PVC electrolyte after 1 h of polymerization.

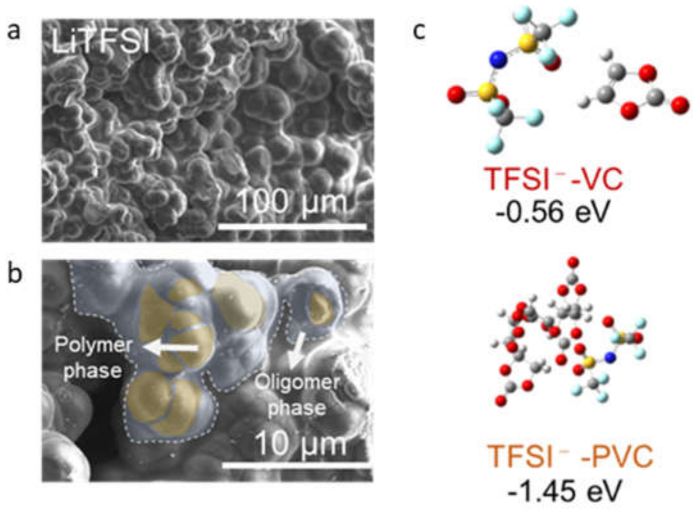

Figure 1. (a,b) SEM images at different magnification of LiTFSI-regulated PVC electrolyte after in-situ polymerization. (c) The binding energy of $TFSI^-$ coordinated VC and PVC, respectively.

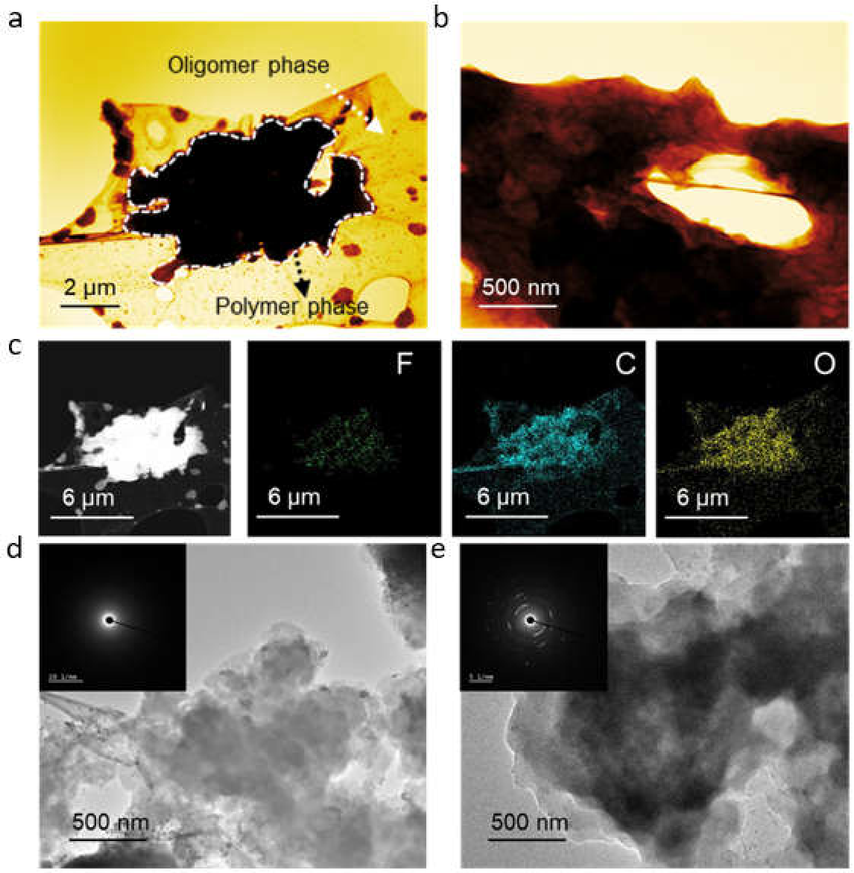

Figure 2. (a,b) High-resolution TEM images and (c) EDS mappings of PVC-24h electrolyte. (d,e) High-resolution Cryo-TEM images and corresponding FFT patterns of PVC-24h electrolyte in different areas.

Reviewer #1-Comment 2: In the ^{7}Li NMR spectra, the authors report the appearance of an additional peak shifting downfield from −0.3 ppm to −0.1 ppm. This observation is interpreted as evidence for two distinct Li^+ solvation environments: one dominated by the precursor and the other by PVC. The downfield shift associated with the polymer-coordinated Li^+ is reasonably explained by the reduced C=O–Li^+ interactions, which would weaken Li^+ coordination and consequently shift the resonance downfield. While this explanation is plausible for Li^+, it remains less convincing for $TFSI^-$. Unlike Li^+, $TFSI^-$ carries an overall negative charge, and its interactions with C=O groups are not straightforward. It is therefore unclear how $TFSI^-$ is proposed to interact specifically with PVC. Moreover, the rationale for why this interaction, with PVC rather than with the solvent VC, would dominate is not sufficiently addressed in the manuscript. Although MD simulations presented in the study suggest the existence of such interactions, a more detailed mechanistic explanation of the driving force behind the $TFSI^-$–PVC association would strengthen the authors' interpretation.

Response: Thanks very much for your insightful comments. Undoubtedly, elucidating the specific interaction mechanism between $TFSI^-$ and PVC/VC is crucial to our work, which has been further investigated elaborately in the revised manuscript. Raman spectroscopy results reveal that with prolonged polymerization time, the -CF_3 and -S-N-S- groups in $TFSI^-$ exhibit significant synchronous shifts, whereas the -C-O-C- groups in VC and PVC do not shift correspondingly (Figure S6a-c). This indicates that $TFSI^-$ is unlikely to interact directly with the carbonate groups in VC/PVC. Therefore, the Raman peak shifts of $TFSI^-$ are mainly attributed to electrostatic interactions with the =C-H/-C-H groups in VC and PVC, forming weak hydrogen bonds, which are further enhanced as the polymer phase content increases by increasing polymerization time.

To further elucidate the interaction mechanism between $TFSI^-$ and PVC/VC, PVC electrolytes with varying concentrations of LiTFSI have been prepared. As shown in Figure S7, the chemical shift of H1s in PVC/VC significantly moves upfield with increasing lithium salt concentration, which is associated with the coordination between $TFSI^-$ and hydrogen atoms. This finding is also corroborated by 2D NMR heteronuclear correlation spectroscopy (HETCOR) (Figure S8), which demonstrates a distinct interaction between H and F atoms. Notably, cross-peaks appear between F atoms (from $TFSI^-$) and the H atoms (from -CH=CH- in VC and the resulting -CH-CH- in PVC), providing direct evidence of the interaction between $TFSI^-$ and both PVC and VC. Furthermore, the strength for the formed hydrogen bond primarily depends on the electropositivity of the hydrogen atom, and stronger electropositivity enhances the electrostatic attraction with electronegative

atoms such as F, facilitating hydrogen bond formation. Compared to the sp^2-hybridized carbon (=C-H) in VC, the sp^3-hybridized carbon (-C-H) in PVC exhibits lower electronegativity, resulting in a weaker attraction for the electron cloud of the hydrogen atom. This allows the hydrogen atom to retain a greater share of the electron cloud. More importantly, the lower electronegativity of carbon leads to reduced polarity in the C-H bond, which consequently increases the partial positive charge on the hydrogen atom. This enhanced partial positive charge promotes the formation of weak hydrogen bonds with highly electronegative F atoms in $TFSI^-$.

In conclusion, $TFSI^-$ exhibits a stronger tendency to interact with PVC due to the facilitated formation of weak hydrogen bonds between them in comparison with VC, which is consistent with the higher binding energy of PVC than VC in DFT simulations (Figure 1c). As the PVC complexed with $TFSI^-$ continuously precipitates from the precursor solution, the newly synthesized PVC will continue to complex with the remaining $TFSI^-$ and precipitate by means of the concentration differences. The relevant contents have been added to lines 108-137 of the revised manuscript.

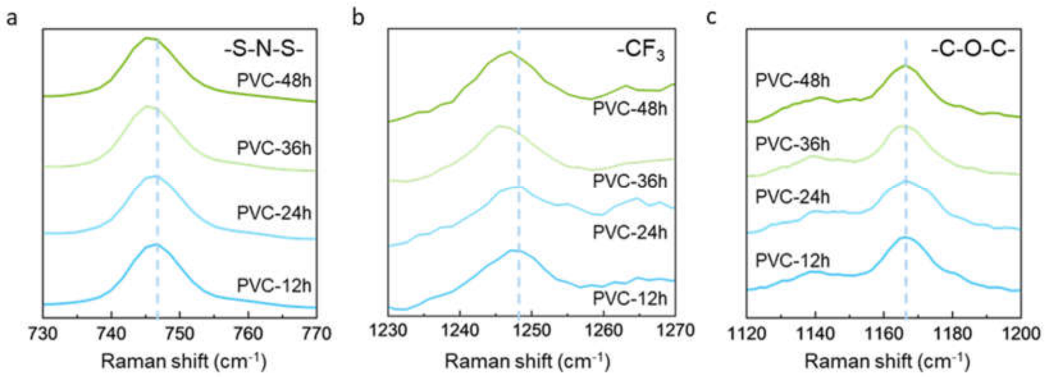

Figure S6. Raman spectra of (a) -S-N-S-, (b) -CF_3 and (c)-C-O-C- in various PVC electrolytes with different polymerization time.

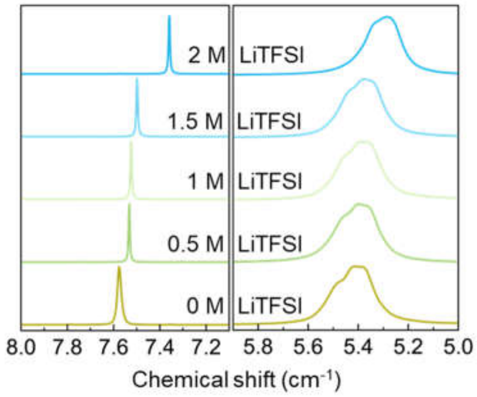

Figure S7. ^1H NMR spectra of PVC electrolytes with varying concentrations of LiTFSI.

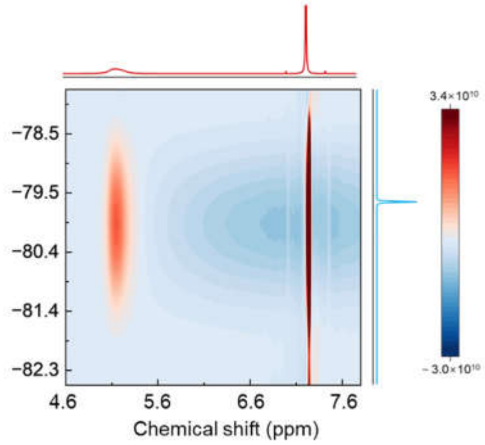

Figure S8. 2D ^{1}H–^{19}F HETCOR NMR spectra of PVC-24h electrolyte.

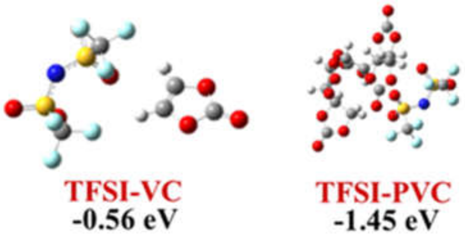

Figure 1. (c) The binding energy between $TFSI^-$ with VC and PVC.

Reviewer #1-Comment 3: The phase evolution described in Fig. 1e,f is interesting and appears different from conventional PIPS. However, it remains unclear whether the observed "two phases" represent genuine phase separation or simply incomplete polymerization/low conversion with a substantial fraction of residual monomer/oligomer. In particular, the text (lines 131–136) states that "in the later stage of polymerization, the polymer phase adsorbs the remaining precursor internally," and the schematic shows an oligomer-rich phase surrounding polymer-rich domains. This may also be interpreted as a natural consequence of partial conversion, where unreacted/oligomeric species remain around polymerized regions, rather than thermodynamically driven phase separation. Please more clearly distinguish the proposed phase separation mechanism from a low-conversion state. Relatedly, the high ionic conductivity in a nominal single-solvent system may arise from a significant fraction of mobile, liquid-like residual oligomers due to incomplete polymerization, rather than from a beneficial phase separation mechanism, this possibility should be discussed.

Response: Thanks very much for your valuable suggestions. It is worth mentioning that the low conversion rate during in-situ polymerization and the single-solvent induced

phase separation mechanism involve distinctly different phase evolution pathways. To clarify this distinction, we have employed different systems to differentiate the structural variations arising from low conversion rates versus the single-solvent induced phase separation. As illustrated in Figure S4, the in-situ polymerization of VC exhibits divergent polymeric architectures with different conditions. Macroscopically, the polymerization process regulated by LiTFSI shows evident precipitation of solid particles, with the solution turning from transparent to turbid. In contrast, the LiDFOB-mediated polymerization system consistently remains clear (Figure R2). Moreover, significant differences are observed in their microstructures: the LiTFSI-based electrolyte displays clear phase-separated morphology, featuring well-defined boundaries between different phases in both SEM and TEM images (Figure 1b and Figure 2a). Conversely, electrolytes incorporating LiDFOB exhibit a homogeneous bulk structure without phase separation (Figure S5).

Furthermore, NMR analysis has been conducted to determine the conversion rate of the LiDFOB-containing electrolyte. The calculated conversion rate is 44.5%, which is notably lower than that of the LiTFSI-regulated system (Figure R3). Despite its lower conversion rate, the PVC-LiDFOB electrolyte does not exhibit phase separation characteristics, and the electrochemical impedance spectroscopy (EIS) of assembled SS//PVC-LiDFOB//SS cell is significantly higher than that with the phase-separated PVC-LiTFSI electrolyte (Figure R4). This indicates that under partial conversion condition, the unreacted oligomers do not spontaneously segregate around the polymer zones to form a phase-separated structure in PVC-LiDFOB electrolyte. Instead, they disperse uniformly within the overall polymer phase, resulting in only a limited enhancement in ionic conductivity. In contrast, the LiTFSI-mediated electrolyte demonstrates clear phase-separation characteristics and then substantially improved ionic conductivity.

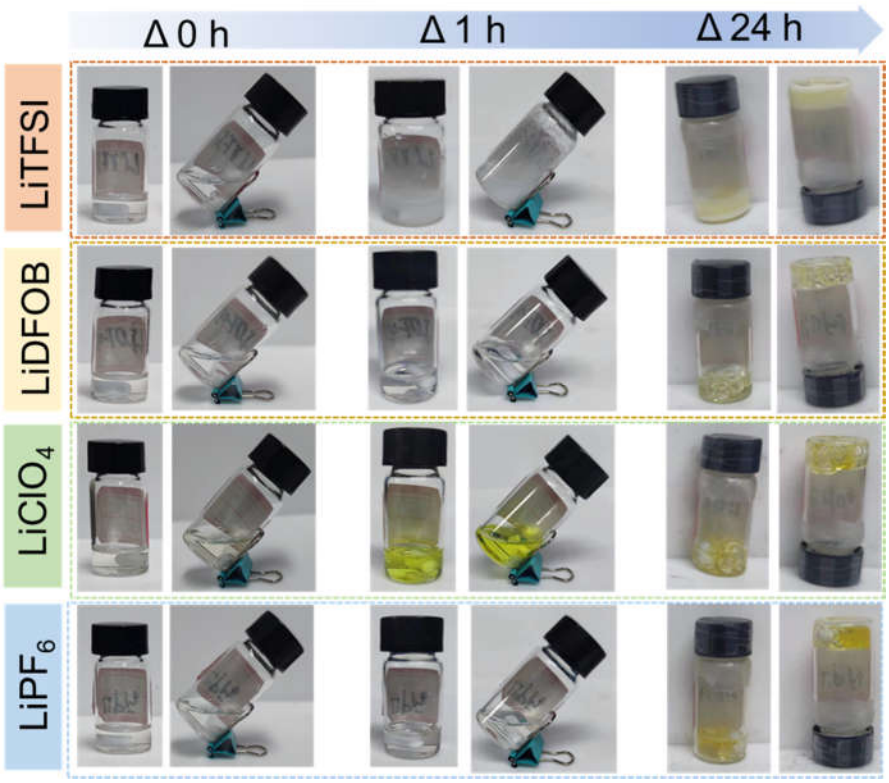

Figure S4. Optical images of PVC electrolytes regulated by different lithium salts at different polymerization time.

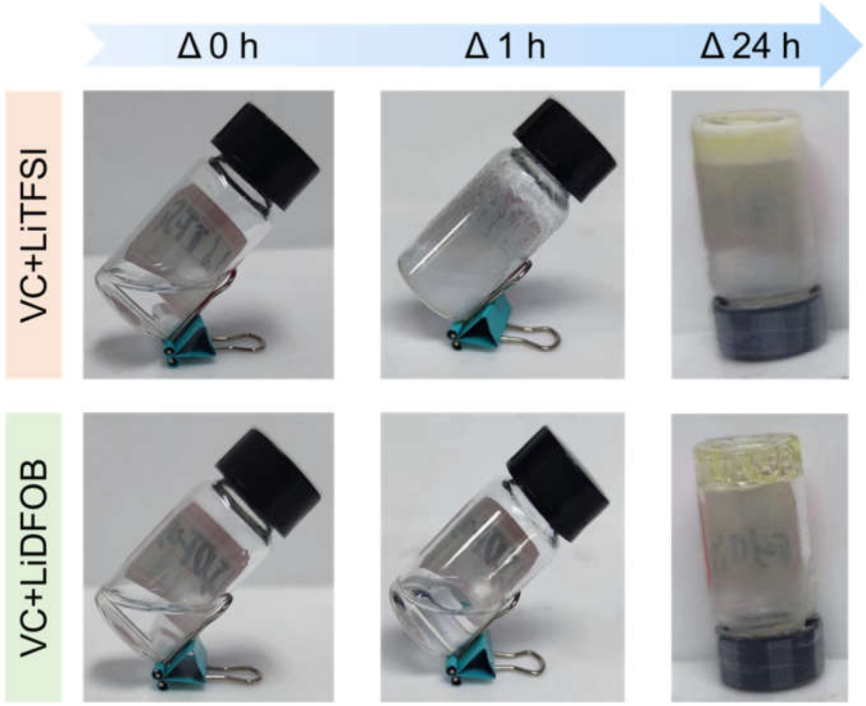

Figure R2. Optical images of LiDFOB-regulated and LiTFSI-regulated PVC electrolytes before and after polymerization at different time.

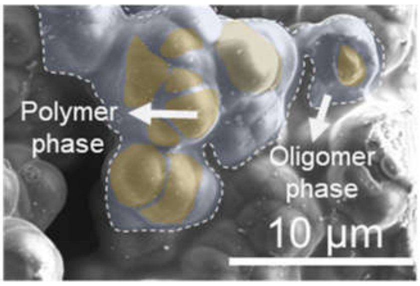

Figure 1. (b) SEM image of LiTFSI-regulated PVC electrolyte after in-situ polymerization.

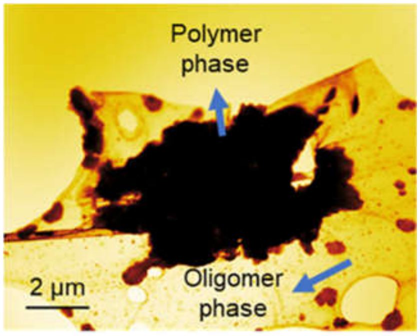

Figure 2. (a) High-resolution TEM image of PVC-24h electrolyte.

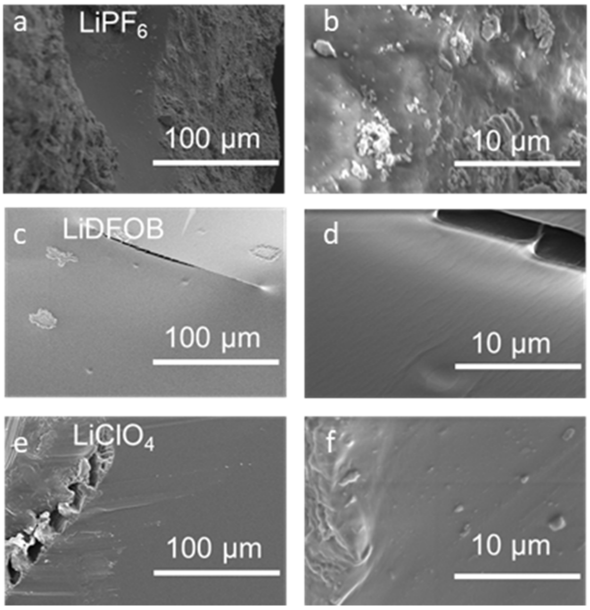

Figure S5. SEM images of the produced various PVC electrolytes with (a-b) $LiPF_6$, (c-d) LiDFOB and (e-f) $LiClO_4$ after in-situ polymerization.

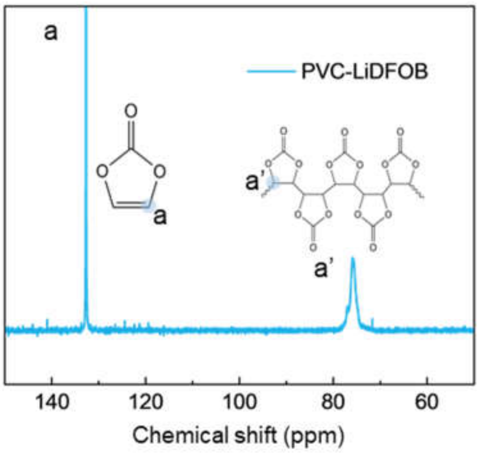

Figure R3. ^{13}C NMR spectrum of LiDFOB-regulated PVC electrolyte.

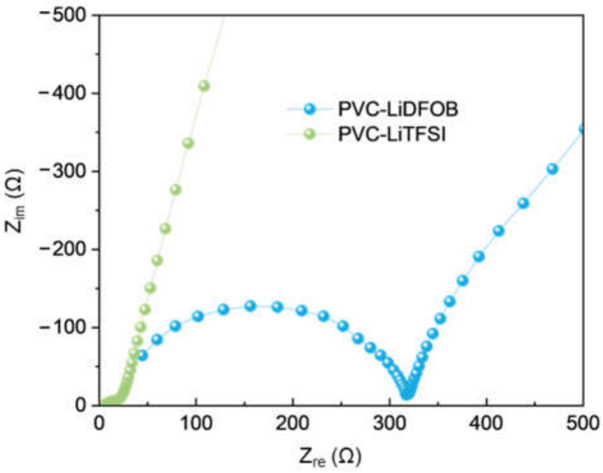

Figure R4. EIS curves of assembled SS//PVC-LiDFOB//SS and SS//PVC-LiTFSI//SS cells at 25 ℃.

Reviewer #1-Comment 4: Fig. S6 presents FTIR spectra before and after polymerization, and the changes in the –C=O and –C–O–C regions are used to support the successful synthesis of PVC. However, VC polymerization is not a ring-opening polymerization. Therefore, the cyclic carbonate carbonyl and alkoxy oxygen functional groups remain present and their FTIR bands are not expected to disappear. Please clarify what specific FTIR changes are diagnostic of polymerization here. Similarly, in Fig. S7b, the reason for the change in the VC, –C=O signal requires explanation. Along the same line, the conversion in Fig. 2g is calculated by integrating ^{13}C NMR peaks at 153.8 ppm and 152.3 ppm, which appear to correspond to carbonyl carbons and may not change dramatically upon polymerization. Why was conversion not quantified using peaks directly associated

with the C=C group, which should provide a clearer before/after distinction?

Response: Thanks very much for your valuable suggestions. We apologize for the confusion caused by our previous unclear descriptions. We wish to clarify that the variation in the intensity of the -C=C- peak in the FTIR spectra can be used to monitor the in-situ polymerization process of VC (Figure S2). After polymerization, the signal of the -C=C- peak (around 1650 cm^{-1}) almost disappears, while the characteristic peaks of -C=O and C-O-C remain. This indicates that the polymerization involves the consumption of -C=C-, without altering the fundamental molecular structure of VC.

Regarding the signal change of -C=O in the original Fig. S3b, since the polymerization of vinylene carbonate proceeds primarily via addition polymerization through the double bond, the reaction takes place at the vinyl C=C group. Although the C=O group does not participate directly in the polymerization reaction, certain changes occur in its chemical environment. This property can serve as an effective means to evaluate the polymerization degree of VC. Observing whether the carbonyl peak undergoes a noticeable shift can help confirm the successful progression of the polymerization reaction (*Adv. Sci.*, 2017, 4, 1600377).

To more accurately determine the conversion rate of VC after in-situ polymerization at different polymerization time, the solid-state NMR carbon spectra corresponding to the -C=C- group have been carried out and analyzed accordingly. As shown in Figure 2g, the conversion rate of VC increases from 81.1% to 86.4% with prolonged polymerization time from 12 h to 48 h, which also corresponds to the mass loss of the PVC electrolytes before 250°C in TG curves (Figure R5). This confirms that the in-situ polymerization of VC achieves a relatively high conversion rate and can be modulated by simply adjusting the polymerization time. The relevant contents have been added to lines 87-91 and 213-219 of the revised manuscript.

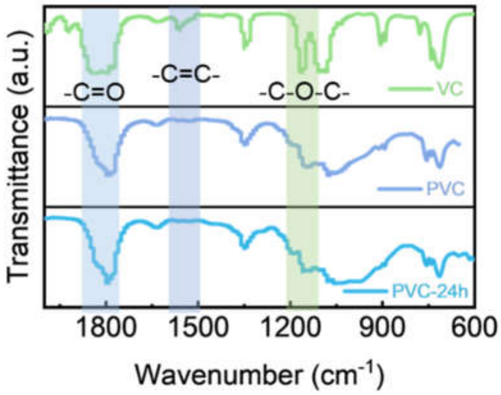

Figure S2. FTIR spectra of VC, PVC polymer and PVC-24h electrolytes.

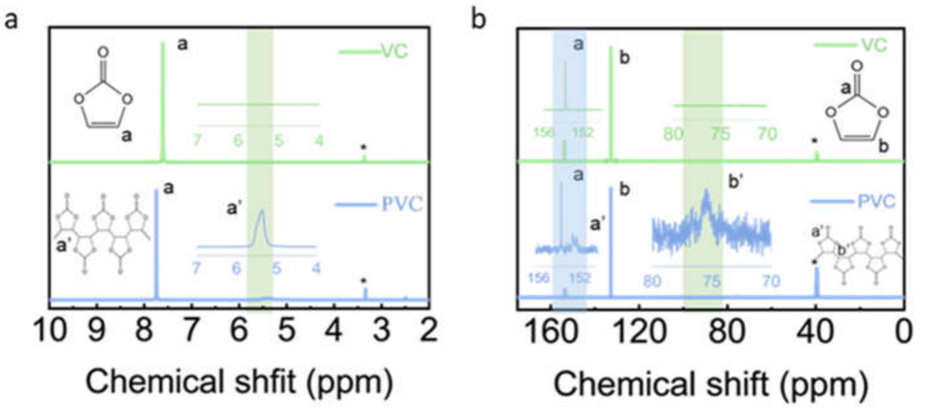

Figure S3. Liquid phase (a) ^{1}H and (b) ^{13}C NMR spectra of VC and PVC electrolyte.

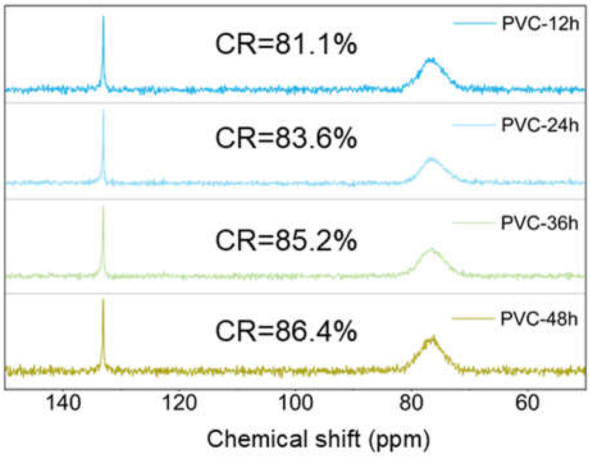

Figure 2. (g) ^{13}C NMR spectra of PVC electrolytes with different polymerization time.

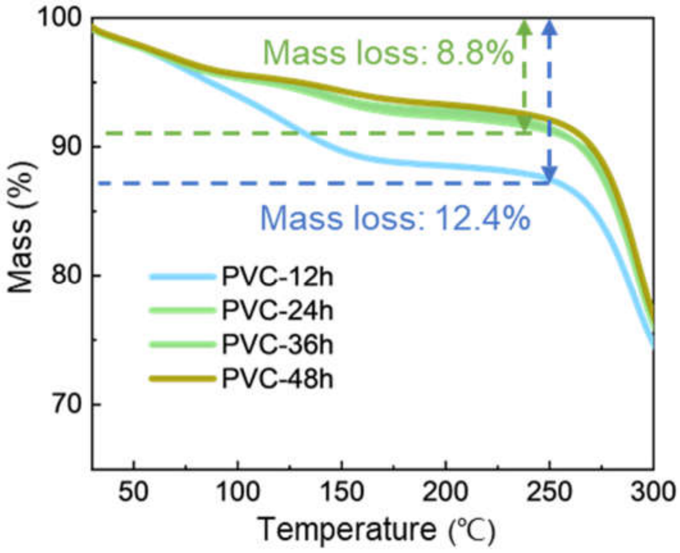

Figure R5. TG curves of various PVC electrolytes with different polymerization time.

Reviewer #1-Comment 5: In Fig. S7a, the vinyl C=C-related peak is labeled as "a" in the VC, ^{1}H NMR spectrum, yet a similarly strong "a" peak remains in the PVC, ^{1}H NMR spectrum. Likewise, in the ^{13}C NMR spectrum, the C=C-related peak ("b") also appears to remain at high intensity after polymerization. This suggests that a substantial fraction of VC may remain unpolymerized. And the polymerization conversion determined by NMR is only 63%, with this degree of conversion, can the material really be regarded as a solid electrolyte? If so, the improved electrochemical performance may be strongly influenced by the presence of liquid-like unreacted/oligomeric components.

Response: We appreciate your feedback and apologize for the previous calculation error that raised concerns about oligomer content in PVC electrolyte. The sharpness of NMR signal is governed by the homogeneity of the chemical environment surrounding the atomic nuclei and their rate of molecular motion. Consequently, even a small amount of monomer can produce intense, sharp peaks due to rapid molecular tumbling, which averages out anisotropic interactions. In contrast, after polymerization, the formed long-chain macromolecules exhibit significantly slower motion. This reduced mobility accelerates the transverse relaxation process, leading to substantial line broadening. For the same number of hydrogen atoms, the NMR signal is dispersed over a wider frequency range, resulting in a pronounced decrease in peak height. According to your previous constructive suggestion, we have recalculated the conversion rate using the more appropriate peak corresponding to the C=C bond in the solid-state ^{13}C NMR spectra (Figure 2g). The recalculated conversion rate for the PVC-24h electrolyte is about 83.6%. This indicates that the combined mass fraction of oligomers and unreacted monomer in the electrolyte is 16.4%. Furthermore, thermogravimetric analysis reveals a mass loss of only 8.8% before 250 °C for PVC-24h electrolyte (Figure R5). Notably, even the PVC-12h electrolyte, which possesses the highest oligomer content, exhibited a lower thermal mass loss of merely 12.4% up to 250 °C. These results confirm the relatively low content of oligomers and residual monomers within the produced PVC-electrolytes. In contrast, the reported PIPS (polymer-induced phase separation) electrolytes typically contain a liquid phase exceeding 40% (*Adv. Energy Mater.*, 2024, 14, 22, 2400428; *Nat. Commun.*, 2023, 14, 2301; *Nature*, 2022, 601, 217). Therefore, our single-solvent-induced phase-separation strategy demonstrates a lower oligomer content alongside better electrochemical performance.

To analyze the impact of monomer residue on electrochemical performance, the EIS of the cell with PVC-LiDFOB electrolyte with low conversion rate but without a phase separation structure has been tested. As shown in Figure R4, the impedance of the assembled cell

using such a non-phase-separated electrolyte is significantly higher than that using the phase-separated PVC electrolyte. In addition, the phase-separated PVC electrolyte was annealed at 200°C for 5 hours to allow full evaporation of residual monomer. The impedance of the SS//PVC-LiTFSI//SS cell using the annealed electrolyte is still lower than that using the PVC-LiDFOB electrolyte (Figure R6). This confirms that the phase-separated structure in our work substantially enhances the ionic conductivity of the electrolyte.

For comparison, the electrochemical performance of the un-polymerized precursor under identical conditions have also been evaluated. LSV shows the oxidation current of VC electrolyte increases significantly at 3.3 V (Figure S19). At a current density of 5C, the coulombic efficiency of the assembled LFP//VC-LiTFSI//Li cell decreases significantly (Figure R7), accompanied by evident electrolyte decomposition (Figure R8). These results indicate that although the unreacted monomer possesses a lower migration energy barrier (Figure 3h), it does not improve the electrolyte performance; conversely, its poor electrochemical stability always leads to a reduction in coulombic efficiency.

In conclusion, the proposed synergistic effect of the phase-separated structure architecture design in our work significantly enhances the overall electrochemical stability and ion conductivity of the PVC-electrolyte. The relevant contents have been added to lines 213-219 of the revised manuscript.

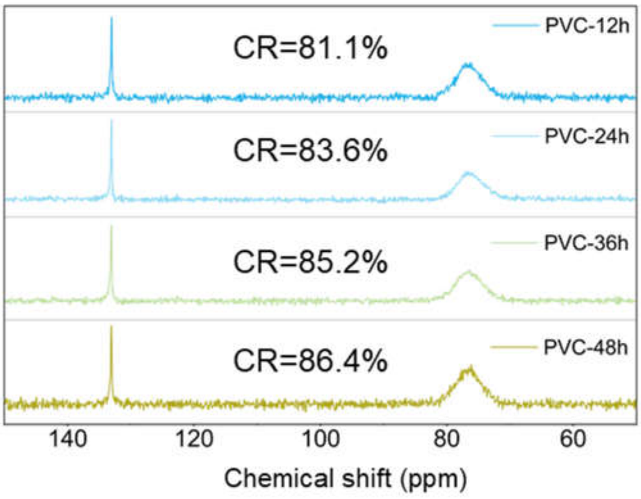

Figure 2. (g) ^{13}C NMR spectra of PVC electrolytes with different polymerization time.

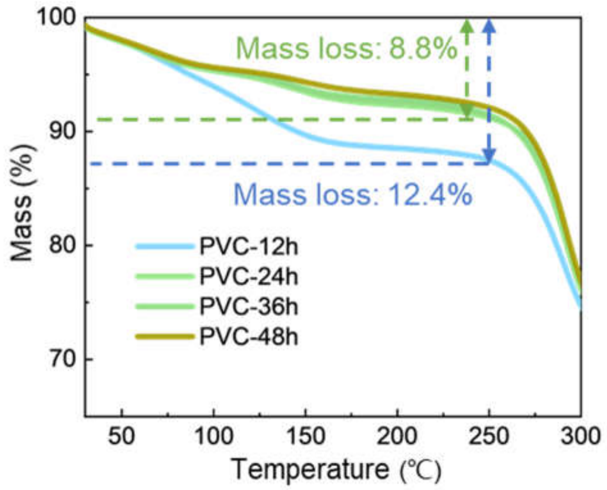

Figure R5. TG curves of various PVC electrolytes with different polymerization time.

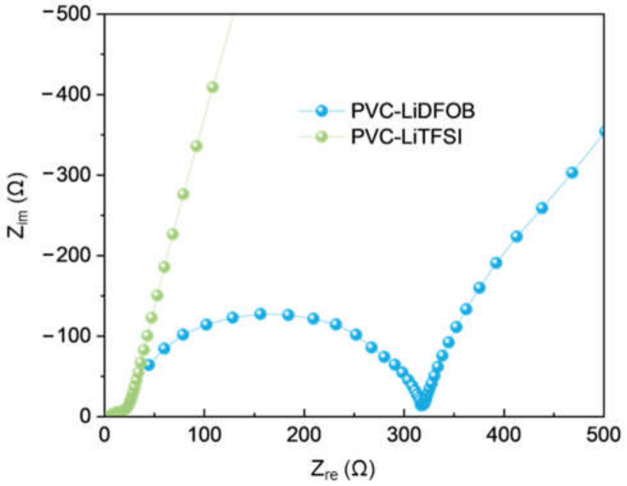

Figure R4. EIS curves of SS//PVC-LiDFOB//SS and SS//PVC-LiTFSI//SS cells at 25 ℃.

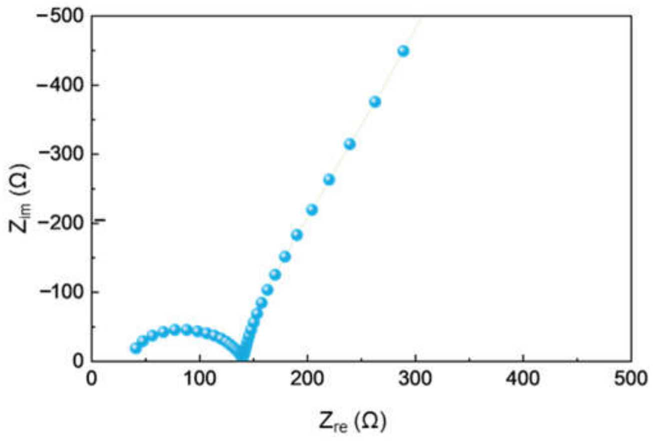

Figure R6. EIS curve of SS//PVC-LiTFSI//SS cell using the annealed PVC electrolyte at 25 ℃.

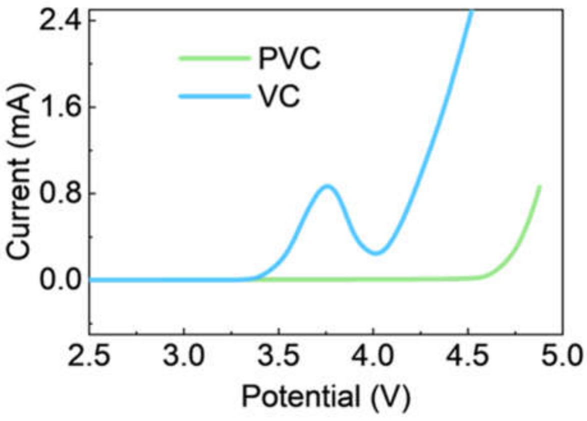

Figure S19. LSV profiles of VC and PVC electrolytes.

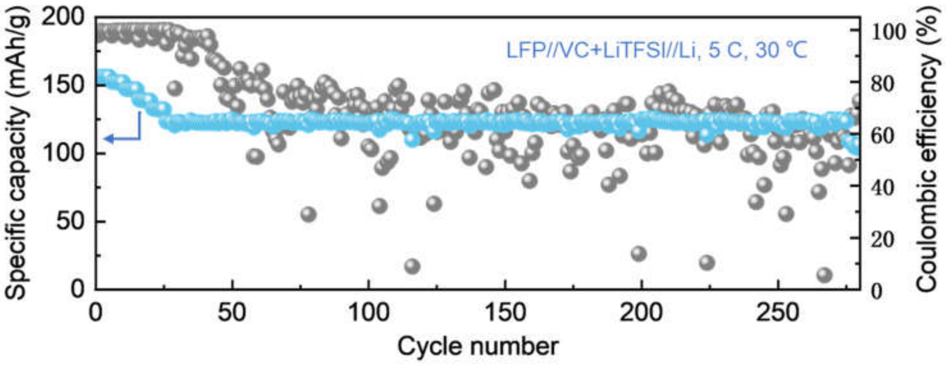

Figure R7. Cycle performance of the assembled LFP//VC-LiTFSI//Li cell at 5 C and 30 °C.

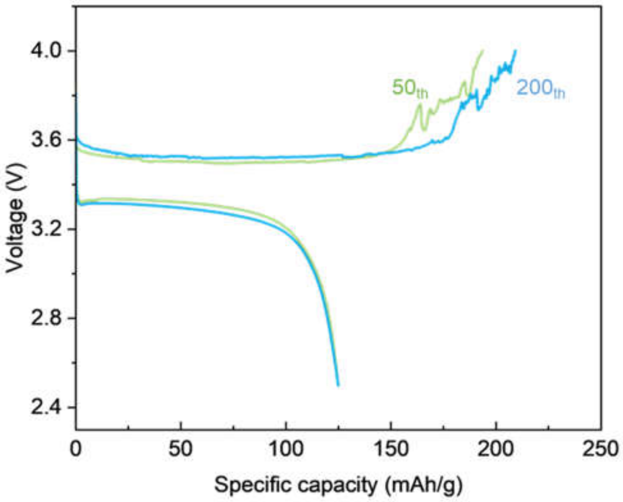

Figure R8. Charge-discharge curves of LFP//VC-LiTFSI//Li cells at 50_{th} and 200_{th} cycles.

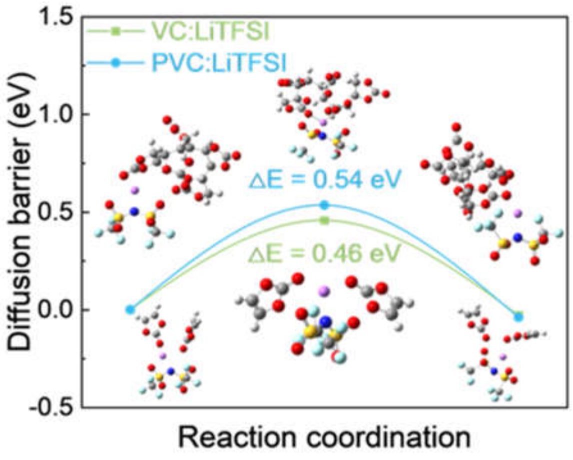

Figure 3. (h) The diffusion barriers of Li between the different coordination structures in polymer phase and oligomer phase.

Reviewer #1-Comment 6: In Fig. S10b, the manuscript interprets the upfield shift in ^{7}Li NMR with increasing polymerization time as a decrease in Li^+–anion coordination number. However, an upfield shift corresponds to increased shielding. Please provide a more rigorous justification for why increased shielding should be interpreted as reduced Li^+–$TFSI^-$ coordination in this system.

Response: Thanks very much for your insightful comments, and we sincerely apologize for any potential ambiguities in the earlier descriptions in the manuscript. In this section, we intend to convey that the strong interaction between PVC and $TFSI^-$ restricts the mobility of the anions while simultaneously weakening the coordination strength between $TFSI^-$ and Li^+ ions (Figure S8). MD calculations further confirm a reduction in the coordination number between $TFSI^-$ and Li^+ ions (Figure 3e and S21a). This implies that more PVC and VC molecules participate in the solvation structure of Li^+ as the decrease in coordination number between $TFSI^-$ and Li^+ ions. Consequently, the lone pair electrons from the carbonyl (C=O) groups in these solvent molecules are preferentially oriented toward the Li^+ ions, compensating for the diminished electron shielding resulting from the weakened $TFSI^-$ coordination. This leads to an increased electron cloud density around the Li^+ nuclei and enhances the electron shielding effect, thereby causing the corresponding signal to shift upfield in the NMR spectrum. The corresponding descriptions have been added in lines 224-230 of the revised manuscript.

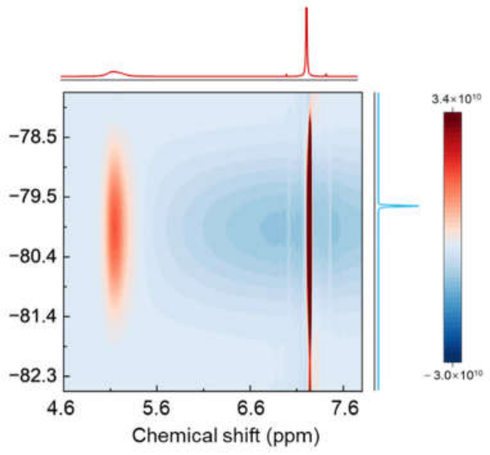

Figure S8. 2D ^{1}H–^{19}F HETCOR NMR spectrum of PVC-24h electrolyte.

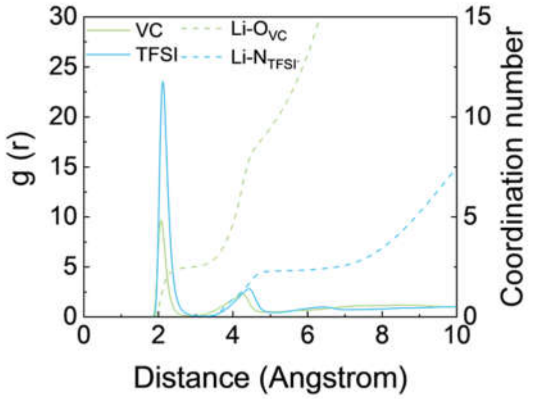

Figure 3. (e) RDF (solid lines) and coordination number (dashed lines) of Li-VC and Li-TFSI^{-} ion pairs of oligomer phase based on theoretical calculations in PVC electrolytes.

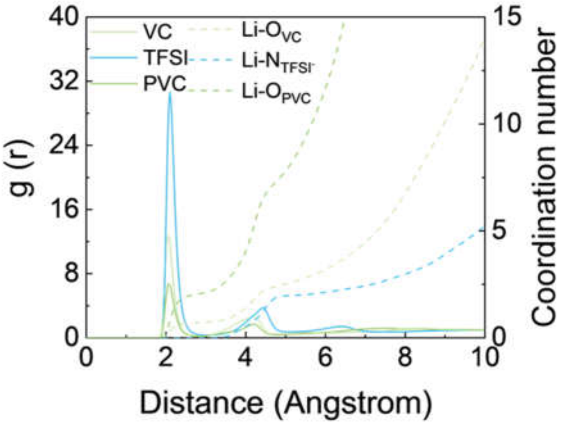

Figure S21. (a) RDF (solid lines) and coordination number (dashed lines) of Li-PVC, Li-VC and Li-TFSI- ion pairs in PVC electrolytes based on theoretical calculations.

Reviewer #1-Comment 7: In Fig. 2h,i the authors claim that longer polymerization time shifts SAXS features toward higher q-values, indicating a more ordered/compact structure and a deeper degree of phase separation. If the phase-separated morphology becomes more ordered, one might expect more uniform Li^+ transport pathways. However, Fig. 3a shows ionic conductivity decreasing with longer polymerization time, while Fig. S19c shows the Li diffusion coefficient increasing with increasing phase separation degree, which appears inconsistent with the conductivity trend. Since the manuscript (lines 228–230) states that ionic conductivity is directly related to phase separation degree and oligomer-phase content, please clarify the relationship among SAXS-derived structural evolution, oligomer content, Li diffusion, and bulk ionic conductivity, and please provide a consistent mechanistic explanation that accounts for these trends. Additionally, the authors attribute improved low-temperature conductivity to increased crystallinity of high-molecular-weight polymer phases (PVC-48h vs PVC-12h), whereas higher crystallinity is generally expected to hinder ion transport and amorphous domains are typically more favorable. Please clarify why crystallinity would enhance conductivity in this system and provide supporting evidence.

Response: Thanks very much for your insightful comments. In our work, the more ordered/compact structure primarily refers to the polymer phase, as the oligomer phase remains highly amorphous feature throughout the phase separation process. For polymers, a more ordered structure implies higher crystallinity, which can be validated by the molecular weight variations in PVC electrolytes with different degrees of phase separation (Figure S16). As polymerization time increases, the molecular weight of the PVC electrolyte gradually rises, leading to enhanced crystallinity. This increase in molecular weight and crystallinity restricts segmental motion of the polymer chains and hinders charge carrier transport, thereby resulting in a decline in ionic conductivity.

Similarly, enhanced crystallinity is unfavorable for the migration and dissociation of $TFSI^-$ interacting with the polymer, indicating a strengthened restrictive effect of the polymer phase on anions. In phase-separated PVC electrolytes, most $TFSI^-$ anions reside within the polymer phase, which impedes their diffusion and further reduces the number of solvated $TFSI^-$ species involved in ion transport. The diffusion coefficients of PFG-NMR are obtained after dissolving the PVC electrolyte in DMSO-d6, aiming to illustrate the interaction strength between the polymer phase and $TFSI^-$. Coordination between $TFSI^-$ and Li^+ impedes the diffusion of Li^+ under a magnetic field. The weakened Li^+-$TFSI^-$ coordination resulting from increased phase separation degree indirectly enhances the lithium ion diffusion coefficient (Figure S22).

In the phase-separated structure, the oligomer phase serves as a fast transport channel for Li^+ and plays a critical role in ion conductivity. Since the degree of phase separation is modulated by varying polymerization time, prolonged polymerization time leads to further conversion of the oligomer phase into the polymer phase (Figure 2g), and reduces the content of the oligomer phase. Given that the ionic conductivity of the PVC electrolyte is contributed by both the polymer and oligomer phases, the enhanced crystallinity of the polymer phase and the reduced oligomer phase content collectively lead to decreased ionic conductivity as phase separation progresses.

Regarding the ionic conductivity of PVC-48h and PVC-12h at low temperatures, we apologize for the previous misrepresentation, and clarify that PVC-48h electrolyte exhibits increased crystallinity and molecular weight , its impedance of the assembled SS //PVC// SS cell at −20 °C is an order of magnitude higher than that with PVC-12h (Figure S18). The relevant descriptions have been added to lines 273-275 of the revised manuscript.

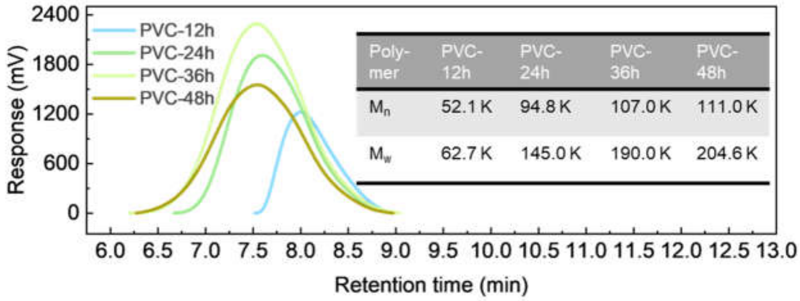

Figure S16. Molecular weight of PVC electrolytes with different polymerization time.

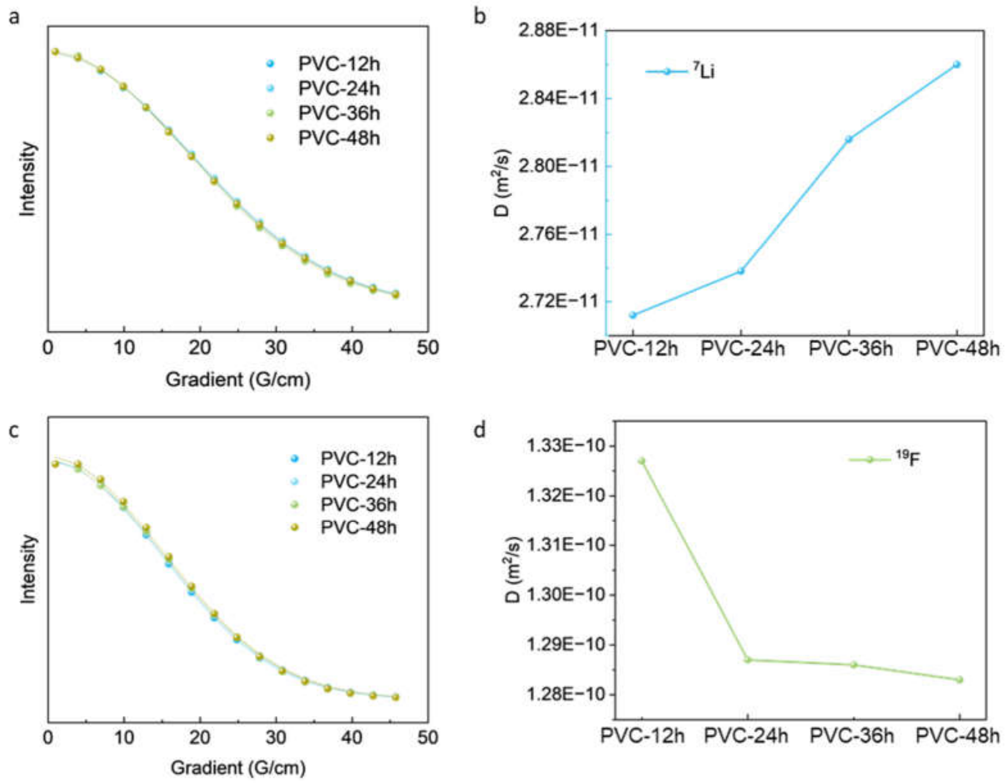

Figure S22. The PFG NMR echo decay profiles and diffusion coefficients for (a-b) ^{7}Li and (c-d) ^{19}F in PVC electrolytes with different polymerization time.

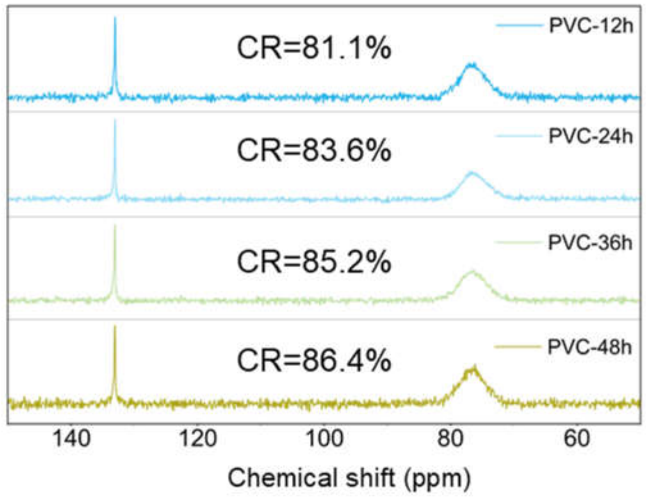

Figure 2. (g) ^{13}C NMR spectra of PVC electrolytes with different polymerization time.

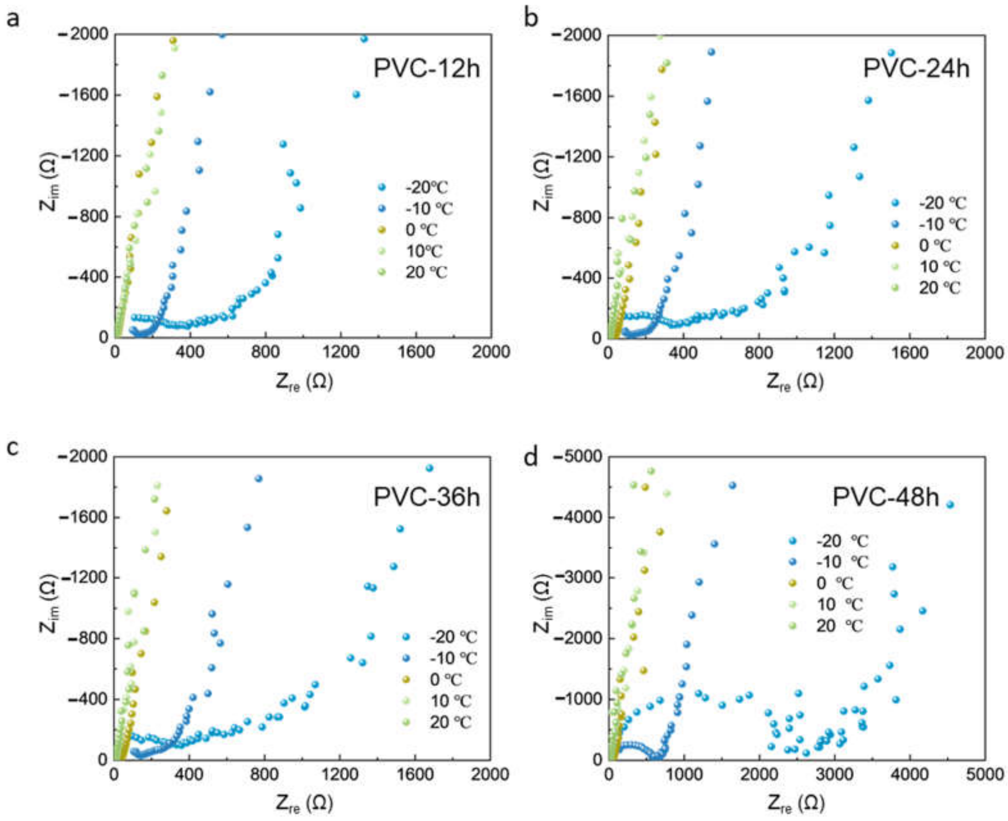

Figure S18. EIS curves of SS //PVC// SS cells with different polymerization time from -20 to 20 °C.

Reviewer #1-Comment 8: The Arrhenius plot in Figure 3b shows that the data points do not strictly adhere to the linear fit, indicating that the chosen model may not accurately represent the temperature dependence of the ionic conductivity. Please re-examine the data fitting to ensure the determined activation energy is accurately calculated based on the data points. And, the X-axis label in Figure 3b contains a spelling error ("Temproture").

Response: Thanks very much for your valuable suggestions. To more accurately characterize the temperature dependence of ionic conductivity and determine the activation energy, we have retested and calculated the activation energies for electrolytes with varying degrees of phase separation. The results demonstrate that as phase separation degree intensifies, the activation energy increases from 1.46 eV to 2.10 eV (Figure 3b). This trend is consistent with the previous result and further confirms the superior low-temperature ion transport performance of oligomer-rich phase. And the small spelling error in the X-axis label in Figure 3b has been corrected. The relevant contents have been added to lines 277-280 of the revised manuscript.

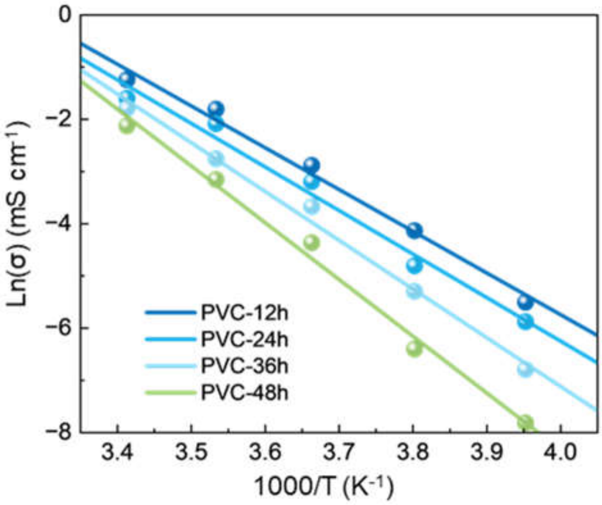

Figure 3. (b) Arrhenius plots at elevated temperatures of PVC electrolytes with different polymerization time.

Reviewer #1-Comment 9: In lines 280–282, the authors state that weakened Li^+–$TFSI^-$ interaction suppresses anion mobility and consequently improves the Li^+ transference number. Please clarify why weakening Li^+–$TFSI^-$ interaction would directly lead to suppressed anion mobility. Even if Li^+–$TFSI^-$ association is weakened, $TFSI^-$ could still dissociate and migrate independently, potentially contributing to concentration polarization. A more detailed mechanistic explanation and supporting evidence are needed to justify this statement.

Response: Thanks very much for your valuable suggestions. We sincerely apologize for any misunderstandings that may have resulted from the incomplete description provided previously. As mentioned earlier, the enhanced PVC-$TFSI^-$ interaction restricts the migration of $TFSI^-$ anions and reduces the Li^+-$TFSI^-$ coordination number (Figure 3e and S21a). 2D NMR HETCOR spectroscopy confirms that the presence of weak hydrogen-bonding interaction between PVC and $TFSI^-$ effectively restricts $TFSI^-$ migration (Figure S8). Additionally, Raman spectroscopy results indicate that as the degree of phase separation increases, the -S-N-S- and -CF_3 groups of the anion undergo more significant shifts (Figure S6). This suggests a strong interaction of $TFSI^-$ within the solvation structure. PFG-NMR results also reveal a gradual decrease in the diffusion coefficient of F (from $TFSI^-$) with increasing phase separation degree (Figure S22c,d). Consequently, the

weakened Li^+-$TFSI^-$ interaction in phase-separated PVC-based electrolyte suppresses anion mobility and resultantly improves the Li^+ transference efficiency.

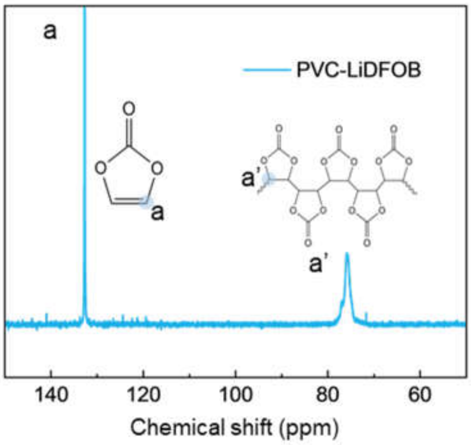

Figure R3. ^{13}C NMR spectrum of LiDFOB-regulated PVC electrolyte.

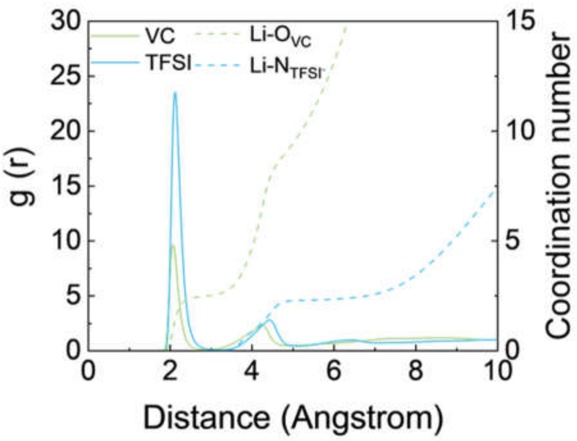

Figure 3. (e) RDF (solid lines) and coordination number (dashed lines) of Li-VC and Li-$TFSI^-$ ion pairs of oligomer phase based on theoretical calculations in PVC electrolytes.

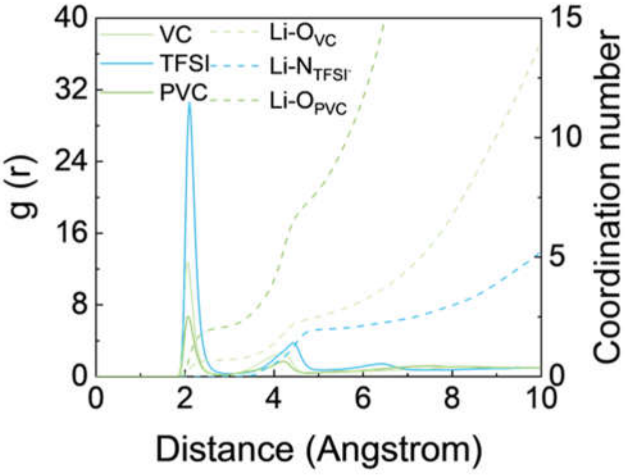

Figure S21. (a) RDF (solid lines) and coordination number (dashed lines) of Li-PVC, Li-VC and Li-TFSI- ion pairs in PVC electrolytes based on theoretical calculations.

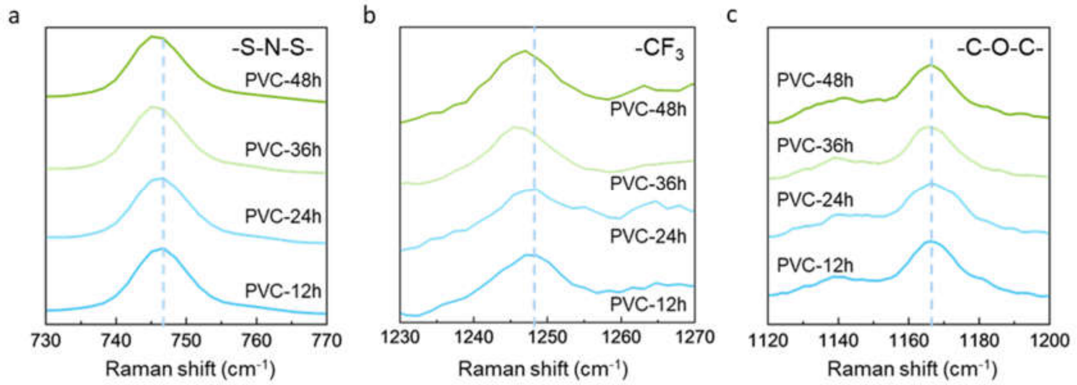

Figure S6. Raman spectra of (a) -S-N-S-, (b) -CF_3 and (c)-C-O-C- in PVC electrolytes with different polymerization time.

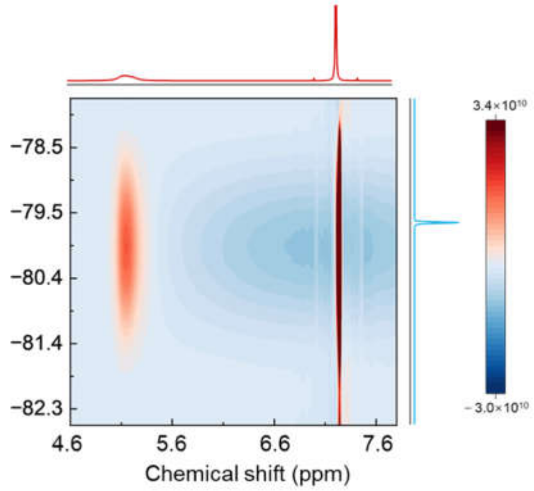

Figure S8. 2D ^{1}H–^{19}F HETCOR NMR spectra of PVC-24h electrolyte.

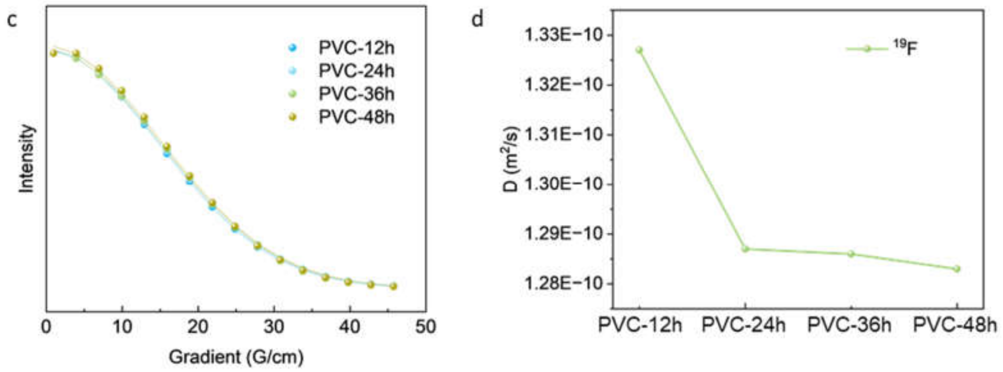

Figure S22. (c-d) The PFG NMR echo decay profiles and diffusion coefficients for ^{19}F in PVC electrolytes with different polymerization time.

Reviewer #1-Comment 10: Conventionally, anion-derived inorganic SEI components are understood to originate from $TFSI^-$ species that reside within the primary solvation structure of Li^+—that is, from aggregated Li^+–anion configurations in which the anion is positioned in close proximity to the cation. In contrast, the present study argues that

anions located in the outer shell, spatially separated from Li^+, also contribute to the formation of inorganic SEI. Supporting this claim, Raman spectroscopy indeed reveals an increased population of “free” anions upon polymerization. While this interpretation offers a potentially valuable new perspective on anion-derived SEI formation, it also raises a conceptual inconsistency. According to the authors’ own mechanism, VC polymerization drives $TFSI^-$ to interact with PVC, leading to precipitation of polymer-rich granular domains. The manuscript further attributes the enhanced t_{Li+} to the immobilization of anions within these polymeric particles. If these anions are effectively confined within the PVC-rich phase, their ability to freely diffuse and participate in inorganic SEI formation at the electrode interface becomes questionable. In other words, the proposed immobilization mechanism appears difficult to reconcile with the simultaneous claim that these same anions actively form an inorganic SEI layer. A clearer mechanistic rationale or additional supporting evidence would be necessary to resolve this discrepancy.

Response: Thanks very much for your insightful comments, As validated in our previous responses of Comment 1 and 2, the interaction between PVC and $TFSI^-$ does indeed restrict anion migration (Figure S7). However, it is worth mentioning that not all $TFSI^-$ anions are confined to the polymer phase. As shown in Figure S8, both VC and PVC can form weak hydrogen bonds with $TFSI^-$, and a small fraction of $TFSI^-$ remains within the oligomer phase, participating in the formation of the solvation structure. Additionally, the "outer solvation structure" described in our manuscript does not refer to a non-coordinated solvent molecule arrangement. Instead, due to competitive coordination effects, the Li^+-$TFSI^-$ interaction exhibits weakened coordination strength and an increased spatial distance, while $TFSI^-$ remains within the Li^+ solvation sheath (Figure R9). This suggests that $TFSI^-$ in the PVC-based electrolyte possesses a lower desolvation energy barrier, facilitating its desolvation at the interface and promoting the formation of an inorganic-rich SEI.

To further substantiate this point, we have conducted TOF-SIMS analysis on the lithium metal surfaces of the assembled Li//VC-LiTFSI//Li and Li//PVC-24h//Li cells after identical cycling periods (Figure 6g,h). The lithium metal surface from Li//PVC-24h//Li exhibits a thinner LiF layer compared to that from Li//VC-LiTFSI //Li, which can be attributed to the confinement of a significant portion of $TFSI^-$ within the polymer phase, limiting its decomposition at the lithium metal surface. Concurrently, the reduced intensity and thickness of the Li_2CO_3 signal indicate that $TFSI^-$ in the PVC electrolyte preferentially decomposes to form an inorganic-rich SEI, thereby suppressing further decomposition of solvent molecules. This observation is consistent with the lower

desolation barrier of TFSI⁻ in this system. The relevant descriptions have been added to lines 313-319 and 486-498 of the revised manuscript.

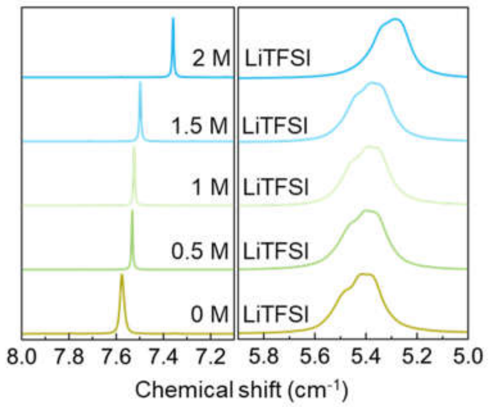

Figure S7. ^{1}H NMR spectra of PVC electrolytes with varying concentrations of LiTFSI.

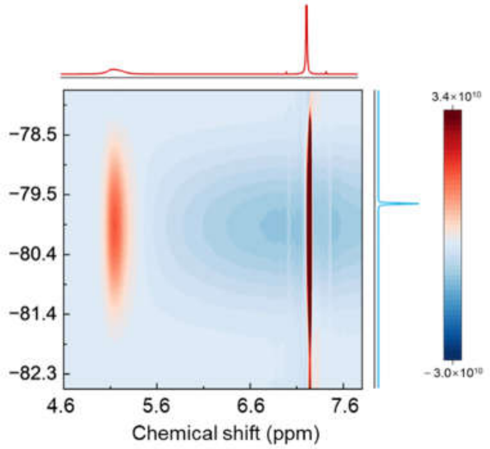

Figure S8. 2D ^{1}H–^{19}F HETCOR NMR spectra of PVC-24h electrolyte.

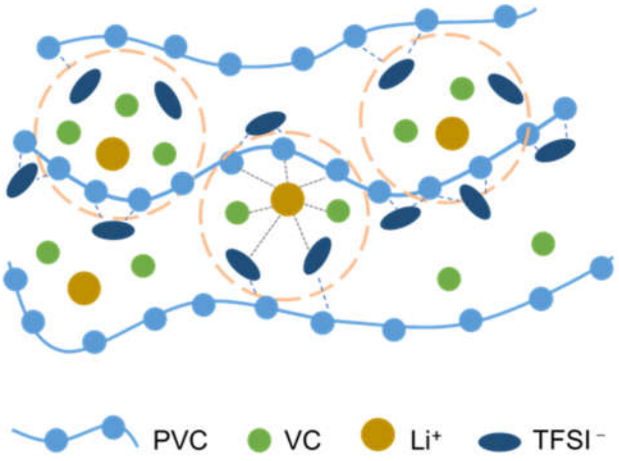

Figure R9. Illustration of the configuration of ionic solvates of PVC electrolyte.

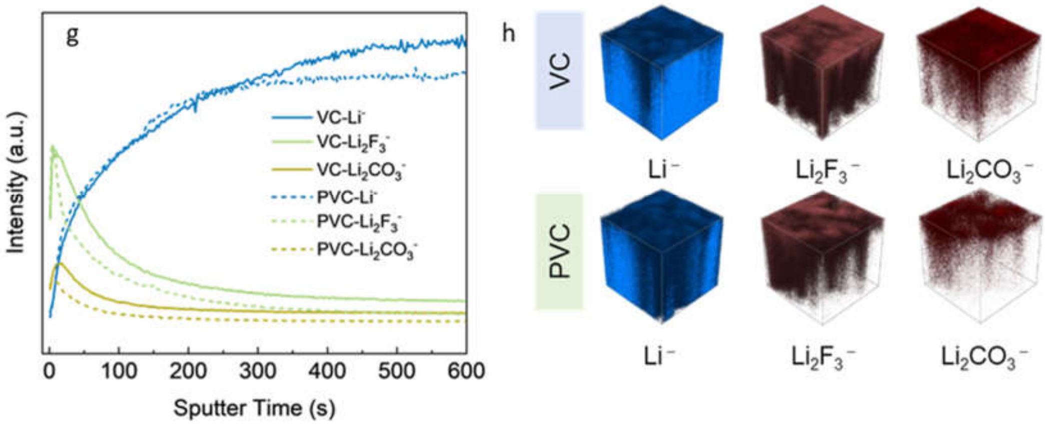

Figure 6. (g) The intensity evolution and (h) 3D renders of Li^-, $Li_2F_3^-$ and $Li_2CO_3^-$ in the TOF-SIMS sputtered volumes of VC-LiTFSI and PVC-LiTFSI induced SEI on the cycled lithium metal anodes.

Reviewer #1-Comment 11:

1. The ionic conductivity reported in the abstract (0.92 mS cm^{-1}) corresponds to PVC-12h, whereas PVC-24h is the main focus. This should be corrected for consistency.

2. In Fig. 1f, "oligmer" should be corrected to "oligomer."

3. Line 294: Please verify the correct figure reference (likely Fig. S20e instead of S18e).

4. The diffusion coefficient is shown in Fig. S19b, not S19c.

5. Line 471: "Preparation of electrodes" should be corrected to "Preparation of electrolytes."

Response: Thanks very much for your valuable comments. We are very sorry for these small language and formatting mistakes. Accordingly, we have carefully checked and made corresponding corrections in the revised manuscript.

Response to the Reviewer #2

General comment: The authors presents a new phase separation strategy to tailor the structure of Poly (Vinylene Carbonate) (PVC)-based electrolyte by lithium salts in a single-solvent, which is quite different with traditional polymerization induced phase separation process that inevitably requires the use of additional phases. More importantly, the precisely regulated separation degree by adjusting the polymerization time ensures tight interface contact between SPEs and electrode materials. Therefore, the assembled batteries exhibits high discharge capacity and superior cycling stability when cycled at a large current density of 5 C and 10 C under room temperature. The results benefit to promote the development of polymer lithium batteries. Overall, the experimental design is novel, and the findings are robust and interesting. I recommend the publication of the work after minor revisions.

Reviewer #2-Comment 1: Regarding the single-solvent phase separation behavior triggered by anions, during the process of regulating the degree of different phase separations in the electrolyte by lithium salts, does the state of the lithium salt anions change?

Response: Thanks very much for your valuable comments. To investigate the state evolution of lithium salt anions during the phase separation process, ^{1}H NMR spectra of PVC-based electrolytes with different LiTFSI concentrations are measured and shown in Figure S7. The results indicate that as increasing LiTFSI concentration, the H1s chemical shift in VC/PVC significantly moves upfield, which is associated with the coordination between $TFSI^{-}$ and hydrogen atoms. The results from two-dimensional NMR heteronuclear correlation spectroscopy (HETCOR) further confirm a notable interaction between H in the electrolyte and F in $TFSI^{-}$ (Figure S8), evidenced by distinct cross-peaks between the F atoms of $TFSI^{-}$ and the hydrogen atoms associated with the carbon-carbon double bonds after polymerization.

Additionally, Raman spectroscopy (Figure S6) of the produced various PVC electrolytes with varying degrees of phase separation show that as phase separation progresses, the Raman peaks corresponding to the -S-N-S- and $-CF_3$ groups from anions exhibit a marked shift to lower wavenumbers. This indicates a strengthened interaction between the polymer phase and $TFSI^{-}$, which consequently restricts the diffusion of $TFSI^{-}$ within the electrolyte. This conclusion is further corroborated by PFG-NMR measurements (Figure S22), which reveal a gradual decrease in the diffusion coefficient of F (from $TFSI^{-}$) with an increased degree of phase separation. Therefore, as the polymerization time increases, the

interaction between the polymer phase and TFSI⁻ anions in the PVC electrolyte is further enhanced. The stronger anchoring effect of PVC on TFSI⁻ further restricts its mobility. The relevant discussions have been added to lines 108-137 and 326-330 of the revised manuscript.

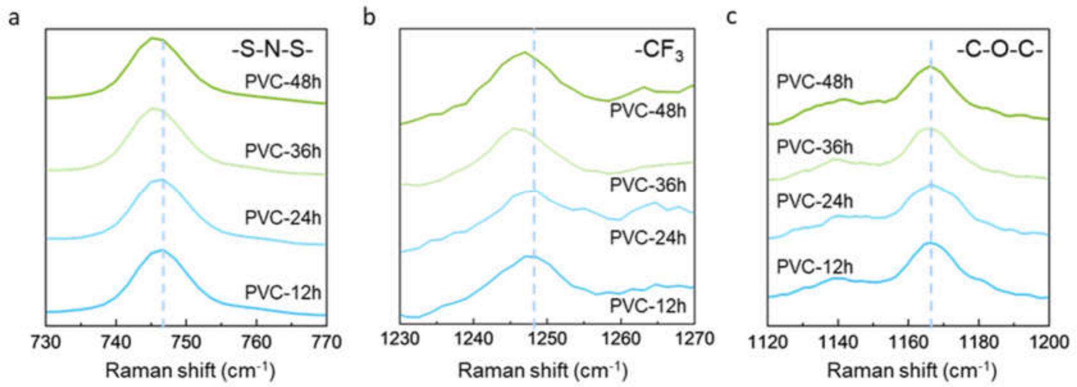

Figure S6. Raman spectra of (a) -S-N-S-, (b) $-CF_3$ and (c) -C-O-C- in PVC electrolytes with different polymerization time.

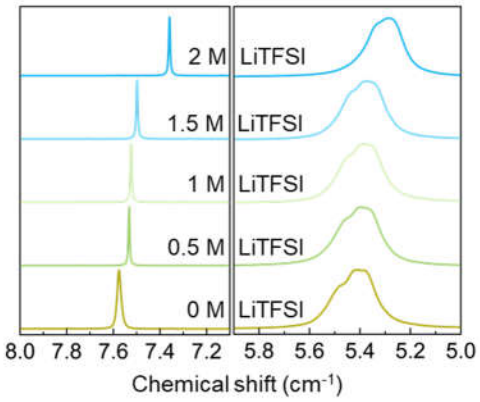

Figure S7. ^{1}H NMR spectra of PVC electrolytes with varying concentrations of LiTFSI.

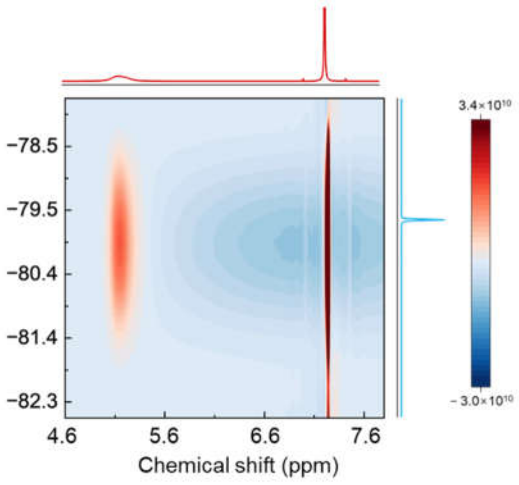

Figure S8. 2D ^{1}H–^{19}F HETCOR NMR spectra of PVC-24h electrolyte.

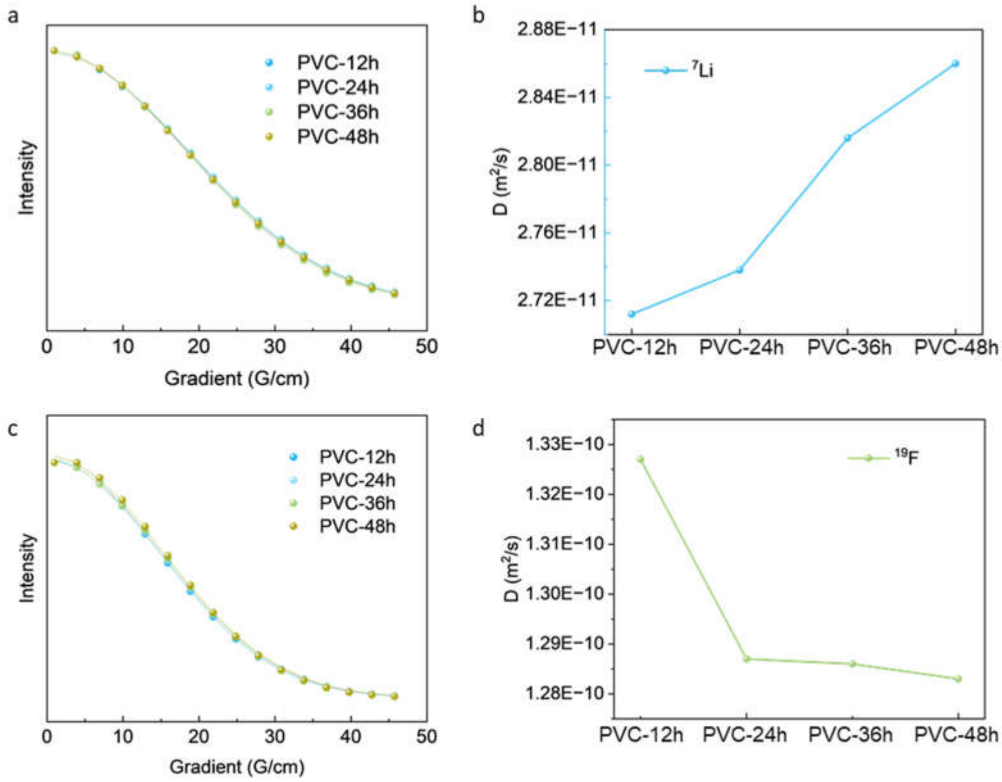

Figure S22. The PFG NMR echo decay profiles and diffusion coefficients for (a-b) ^{7}Li and (c-d) ^{19}F in PVC electrolytes with different polymerization time.

Reviewer #2-Comment 2: Whether the crystallinity of polymer electrolytes with different degrees of phase separation also has a significant impact on the carrier transport performance of the electrolyte should be discussed in the manuscript.

Response: Thanks very much for your valuable suggestions. Undoubtedly, the crystallinity of polymers is crucial for segmental motion and then charge carrier transport. Within crystalline regions, polymer chains are orderly and densely packed with strong intermolecular interactions, whereas amorphous regions feature loosely packed chains with greater mobility. Ion transport occurs primarily through the coordinated motion of polymer segments in the amorphous regions. Namely, excessively high crystallinity would reduce the pathways available for ion migration, leading to a decline in ionic conductivity. On the other hand, crystalline domains provide the mechanical strength necessary to prevent potential lithium dendrite penetration and subsequent short circuits during battery cycling.

To investigate the crystallinity of the produced various PVC electrolytes with different polymerization time, XRD analysis is performed. As shown in Figure S24, the PVC

electrolytes, regardless of their degree of phase separation, predominantly exhibit amorphous characteristics. SAXS results further reveal that an increased degree of phase separation leads to a more ordered structural arrangement (Figure 2h,i and S14). This indicates that modulating the phase-separation degree can enhance the regularity of polymer chain packing while maintaining the amorphous nature of the PVC electrolyte, thereby improving its mechanical integrity and tailoring ion conductivity simultaneously. The relevant discussions have been added to lines 342-345 of the revised manuscript.

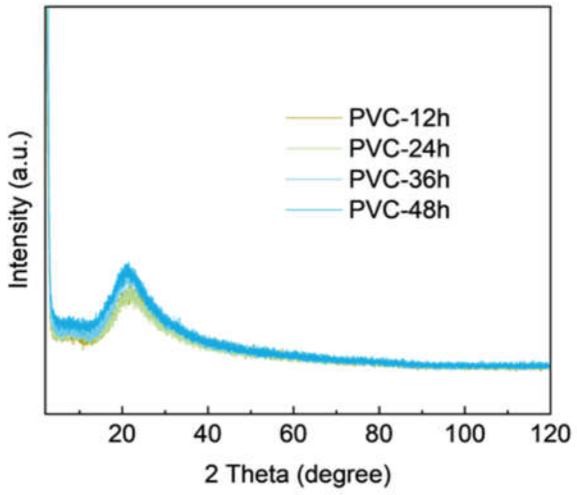

Figure S24. XRD patterns of various PVC electrolytes with different polymerization time.

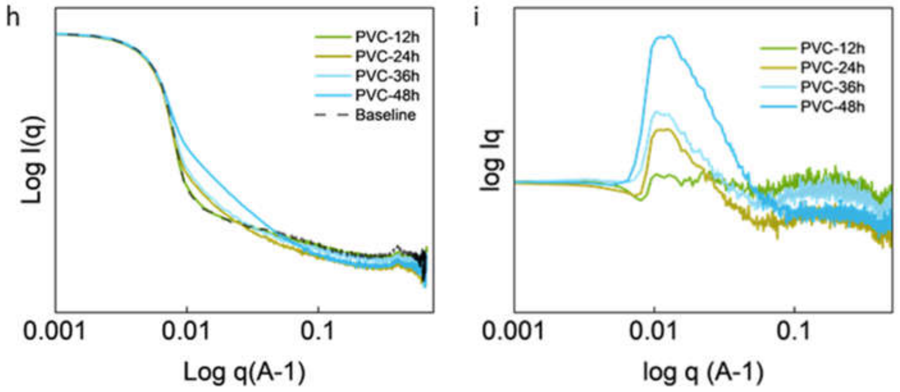

Figure 2. SAXS of various PVC electrolytes with different polymerization time (h) before and (i) after the baseline subtraction.

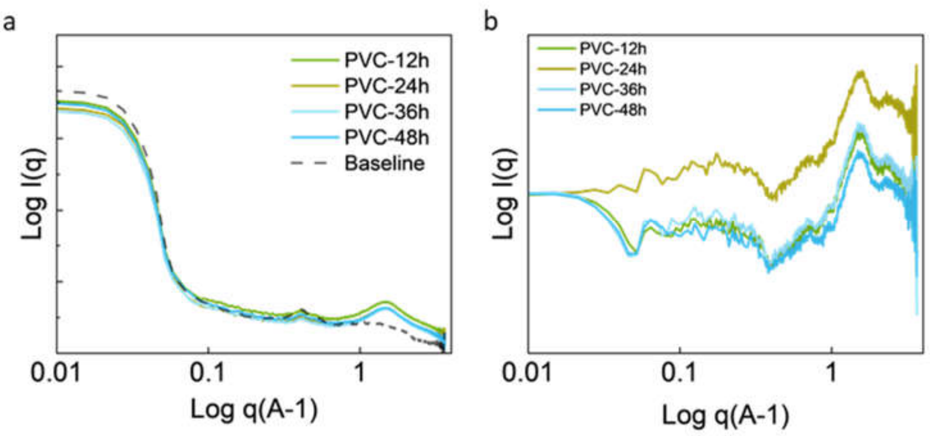

Figure S14. SAXS at large angles of various PVC electrolytes with different polymerization time (a) before and (b) after the baseline subtraction.

Reviewer #2-Comment 3: Details of the experiment is essential for the readers, such as the details of preparation for the test samples as well as the testing environment.

Response: Thank you very much for your kind suggestions. To ensure the accuracy and consistency of the collected data, all samples involved in the manuscript—except those requiring specific preparation conditions—are disassembled from coin cells under identical conditions and stored in an argon-filled glove box. Throughout testing and transfer process, the samples remains well-sealed to prevent exposure to air. Repeated experiments are conducted to verify the reliability of the acquired data. Detailed experimental conditions are documented in line 562-607 of the Supporting Information.

Reviewer #2-Comment 4: The assembled batteries exhibit excellent rate performance and cycle stability, but the intrinsic relationship between the performance and the separation regulation of the electrolyte needs to be discussed in detail.

Response: Thanks very much for your valuable suggestions. The excellent rate capability and cycling stability of the assembled batteries using PVC-based electrolyte originate from its unique phase-separated structure. This architecture effectively combines the high mechanical strength and electrochemical stability of the polymer phase with the high ion-transport properties of the oligomer phase. The single-solvent-induced phase separation strategy proposed in our work can precisely control the phase structure through specific interaction between the polymer and anion, and thus confine most of the anions in the polymer matrix (Figure S8). This confinement further enhances lithium-ion transport

efficiency, which is a prerequisite for the excellent rate performance of the PVC electrolyte-based batteries.

Meanwhile, the improved cycling stability of the PVC electrolyte-based batteries can be attributed to the enhanced mechanical properties of PVC electrolyte achieved by tuning the degree of phase separation, along with the formation of an inorganic-rich SEI. The weak hydrogen-bonding interaction between the polymer and anions introduces a competitive coordination effect between PVC and Li^+ ions (Figure R9), which reduces the mobility and desolvation energy barrier of $TFSI^-$, finally facilitating its decomposition at the interface to form SEI rich in LiF (Figure 6g,h) and then benefitting to improve the cycling stability of batteries. The corresponding discussions have been added in lines 108-137 and 486-498 of the revised manuscript.

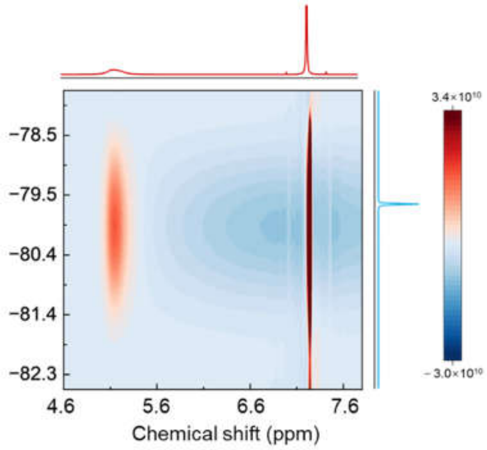

Figure S8. 2D ^1H–^{19}F HETCOR NMR spectra of PVC-24h electrolyte.

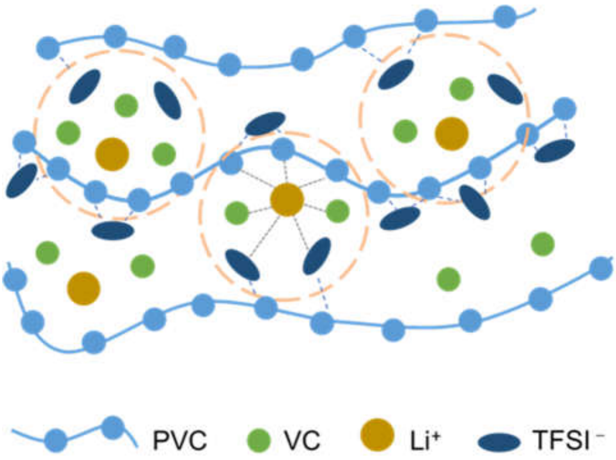

Figure R9. Illustration of the configuration of ionic solvates of PVC electrolyte.

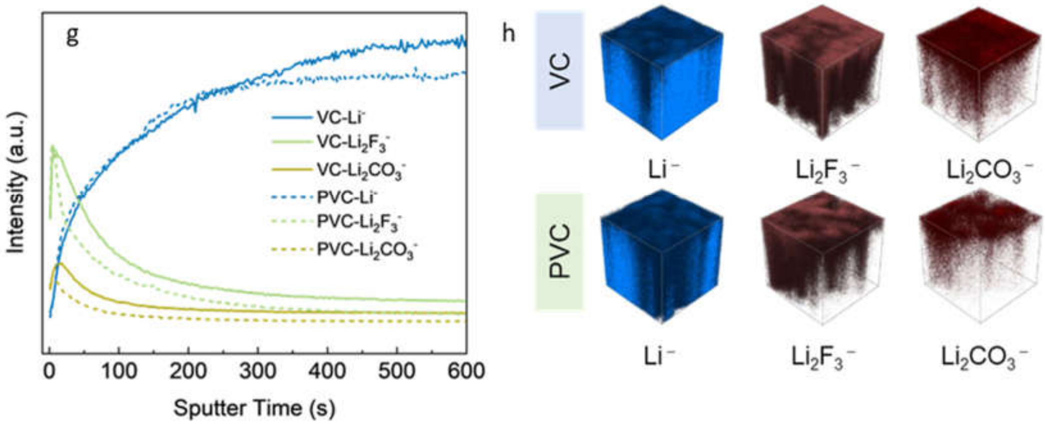

Figure 6. (g) The intensity evolution and (h) 3D renders of Li^-, $Li_2F_3^-$ and $Li_2CO_3^-$ in the TOF-SIMS sputtered volumes of VC-LiTFSI and PVC-LiTFSI induced SEI on the cycled lithium metal anodes.

Reviewer #2-Comment 5: As mentioned above, the phase separation strategy reported in this work is quite different with the traditional polymerization induced phase separation process. The advantages of such a single-solvent strategy should be discussed more elaborately as this is the core innovation of this work.

Response: Thanks very much for your valuable suggestions. The single-solvent induced phase separation mechanism proposed in our work is fundamentally different from previously reported PIPS process. Firstly, PIPS typically requires the pre-mixing of two physically incompatible components, such as a polymer monomer and an ionic liquid, as precursors. This not only increases material costs but also fixes the phase ratio prior to electrolyte synthesis, limiting process flexibility and adaptability. In contrast, our strategy only necessitates the addition of a lithium salt and a single monomer, offering a clear cost advantage. More importantly, phase separation in our system is in-situ induced by the specific interaction between the anion and the growing polymer chain, which not only enables precise solvation structure regulation, but also significantly enhances ion transport rate. Furthermore, such a strategy allows flexible tuning of the phase separation degree by simply adjusting polymerization time, making it adaptable to various operational requirements. The relevant descriptions have been added to lines 53-62 of the revised manuscript.

Reviewer #2-Comment 6: Some language polishing and formatting adjustments are

required for consistency (e.g., There is a formatting error in the vertical axis of Figure 2i in the manuscript).

Response**:** Thanks very much for your valuable comments. We are very sorry for these small language and formatting mistakes. Accordingly, we have carefully checked and made corresponding corrections in the revised manuscript.

Response to the Reviewer #3

General comment: The manuscript "Tailoring electrolyte phase separation for high-rate solid-state lithium metal batteries" presents an innovative in-situ polymerization strategy using a single lithium salt to induce controllable phase separation in a single-solvent system, constructing a high-performance dual-phase solid polymer electrolyte (SPE) for high-rate lithium metal batteries. The study combines theoretical calculations, multiple characterization techniques (XPS, NMR, Raman), microstructural characterizations (SEM, TEM, AFM), and electrochemical tests to elucidate the mechanism of salt-induced phase separation and the excellent electrochemical properties of electrolyte. The battery demonstrates remarkable cycling stability, retaining 90% capacity after 4000 cycles at 5 C. Therefore, the manuscript is considered suitable for publication in Nature Communications. However, a major revision is required before acceptance.

Reviewer #3-Comment 1: The dual-phase structure of the PVC polymer electrolyte includes polymer and oligomer phases, with a proposed Li^+ transport mechanism across both phases. However, the presence of residual VC monomer is not addressed. Please provide a detailed explanation regarding whether residual VC exists, and its contribution to ionic conductivity and transport mechanisms.

Response: Thanks very much for your professional insights. Indeed, the residual VC monomer is present in the produced PVC electrolyte and we classify it as a type of oligomer phase in the manuscript. The conversion rate of VC for the optimal PVC-24h electrolyte is calculated to be about 83.6% using the appropriate peak corresponding to the C=C bond in the solid-state ^{13}C NMR spectra (Figure 2g). This indicates that the combined mass fraction of oligomers and unreacted monomer in the electrolyte is about 16.4%. In addition, thermogravimetric analysis reveals a mass loss of only 8.8% before 250 °C for the PVC-24h electrolyte (Figure R5). Notably, even the PVC-12h electrolyte, which possesses the highest oligomer content, exhibits a minimal thermal mass loss of merely 12.4% up to 250 °C. These results confirm the relatively low content of oligomers and residual monomers within the produced PVC electrolytes.

Furthermore, PVC electrolytes regulated by different lithium salts are used to clarify the role of residual monomers in different electrolyte structures. As illustrated in Figure S4, the in-situ polymerization of VC exhibits divergent polymeric architectures with different conditions. Macroscopically, the polymerization process regulated by LiTFSI shows evident precipitation of solid particles, with the solution turning from transparent to turbid. In contrast, the LiDFOB-mediated polymerization system consistently remains

clear (Figure R2). Moreover, significant differences are observed in their microstructures: the LiTFSI-based electrolyte displays clear phase-separated morphology, featuring well-defined boundaries between different phases in both SEM and TEM images (Figure 1b and Figure 2a). Conversely, electrolytes incorporating LiDFOB exhibit a homogeneous bulk structure without phase separation (Figure S5). Furthermore, NMR analysis has been conducted to determine the conversion rate of the LiDFOB-containing electrolyte. The calculated conversion rate is only 44.5%, which is notably lower than that of the LiTFSI-regulated system (Figure R3). Despite its lower conversion rate, the PVC-LiDFOB electrolyte does not exhibit phase separation phenomenon, and the electrochemical impedance spectroscopy (EIS) of assembled SS//PVC-LiDFOB//SS cell is significantly higher than that with the phase-separated PVC-LiTFSI electrolyte (Figure R4). This indicates that under partial conversion condition, the unreacted oligomers do not spontaneously segregate around the polymerization zones to form a phase-separated structure. Instead, they disperse uniformly within the overall polymer phase, resulting in only a limited enhancement in ionic conductivity. In contrast, the LiTFSI-mediated electrolyte demonstrates clear phase-separation characteristics and a substantially improved ionic conductivity. Additionally, the phase-separated PVC electrolyte was annealed at 200°C for 5 hours to allow full evaporation of residual monomer. The assembled SS//PVC-LiTFSI//SS cell using the annealed electrolyte still remains lower impedance than that using the PVC-LiDFOB electrolyte (Figure R6). This confirms that the phase-separated structure substantially enhances the ionic conductivity of the PVC electrolyte. Furthermore, the electrochemical performance of the un-polymerized precursor under identical conditions have been evaluated. LSV shows the oxidation current of VC electrolyte increases significantly at 3.3 V (Figure S19). At a current density of 5C, the coulombic efficiency of the assembled LFP//VC-LiTFSI//Li cell decreases significantly (Figure R7), accompanied by evident electrolyte decomposition (Figure R8).

These results indicate that although the unreacted monomer possesses a lower activation energy (Figure 3b) and migration energy barrier (Figure 3h), it does not improve electrolyte performance; conversely, its poor electrochemical stability always leads to a reduction in coulombic efficiency. In conclusion, the proposed synergistic effect of the phase-separated structure architecture design in our work significantly enhances the overall electrochemical stability and ion conductivity of the PVC-electrolyte.

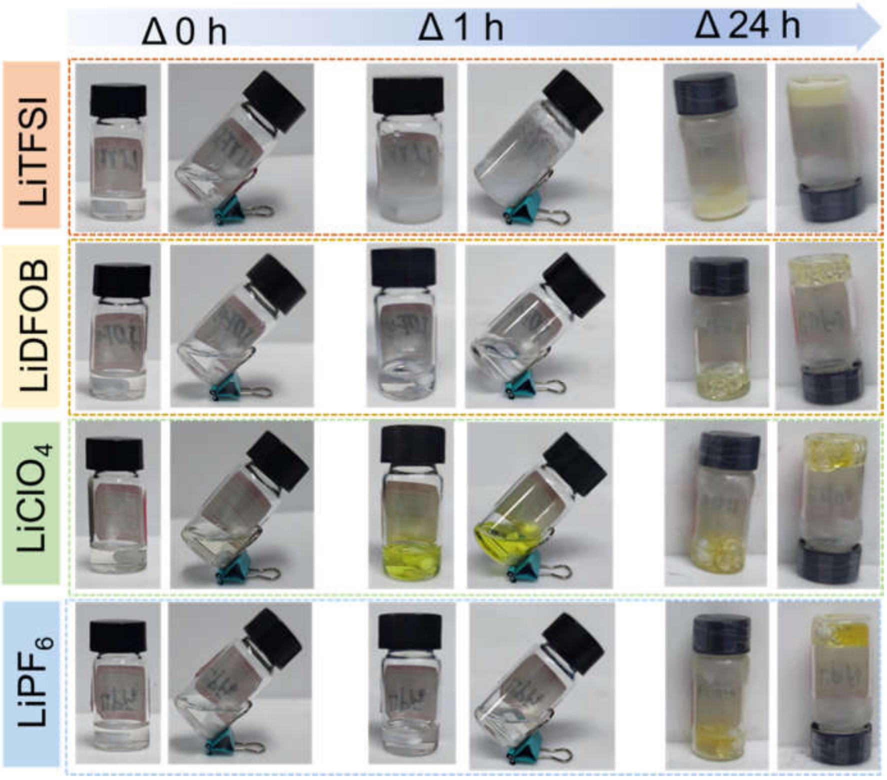

Figure S4. Optical images of PVC electrolytes regulated by different lithium salts at different polymerization time.

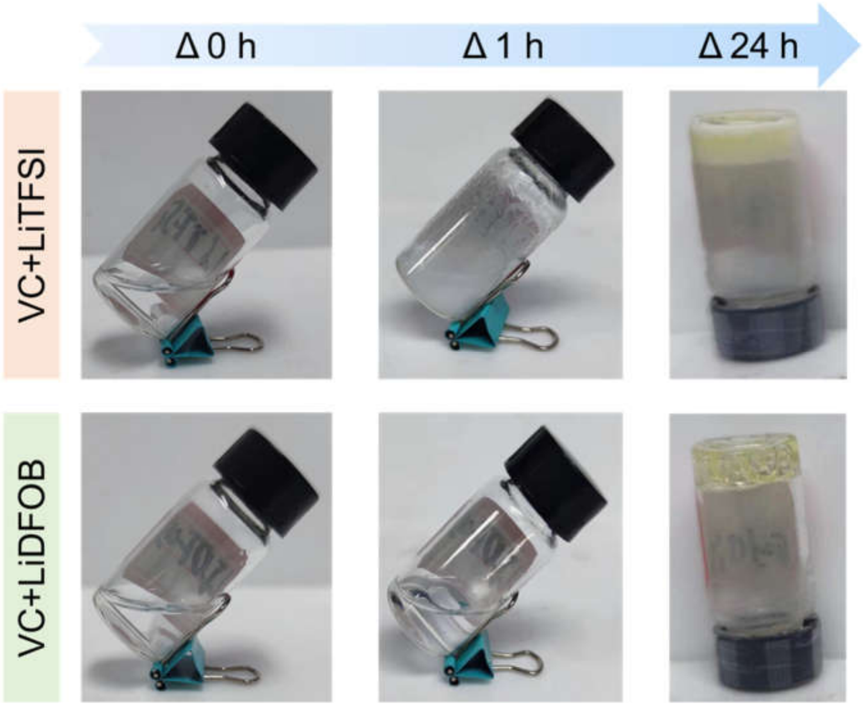

Figure R2. Optical images of LiDFOB-regulated and LiTFSI-regulated PVC electrolytes before and after polymerization at different time.

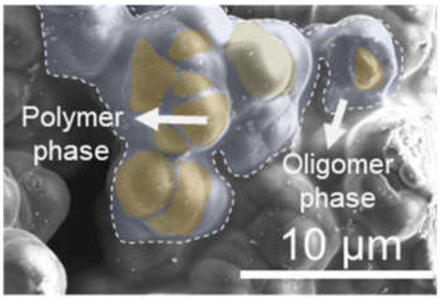

Figure 1. (b) SEM image of LiTFSI-regulated PVC electrolyte after in-situ polymerization.

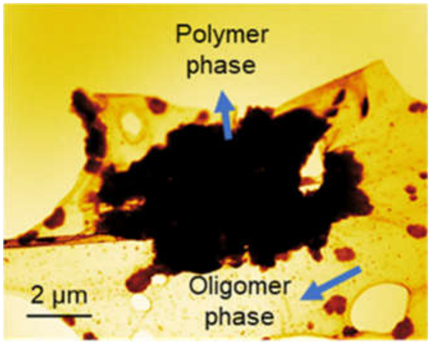

Figure 2. (a) High-resolution TEM image of PVC-24h electrolyte.

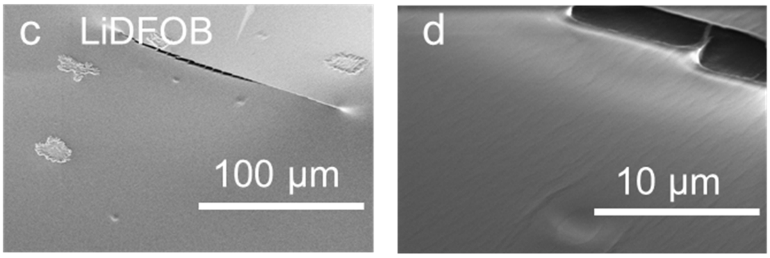

Figure S5. (c-d) SEM images of the produced PVC electrolyte with LiDFOB after in-situ polymerization.

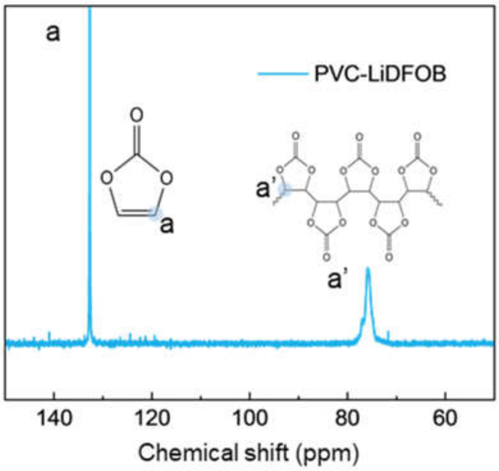

Figure R3. ^{13}C NMR spectrum of LiDFOB-regulated PVC electrolyte.

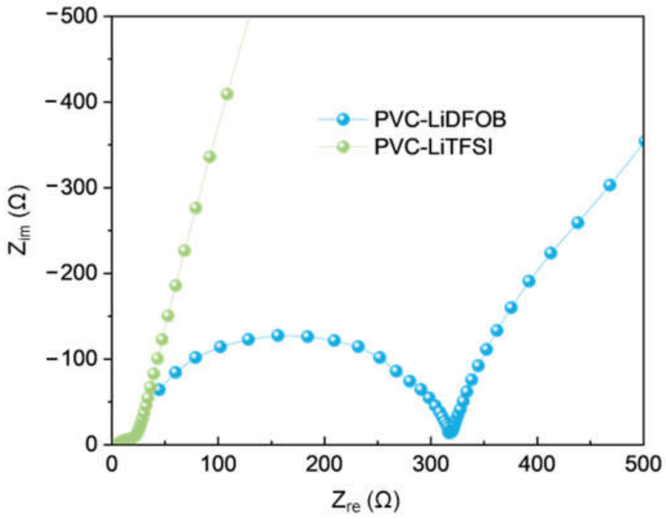

Figure R4. EIS curves of SS//PVC-LiDFOB//SS and SS//PVC-LiTFSI//SS cells at 25 ℃.

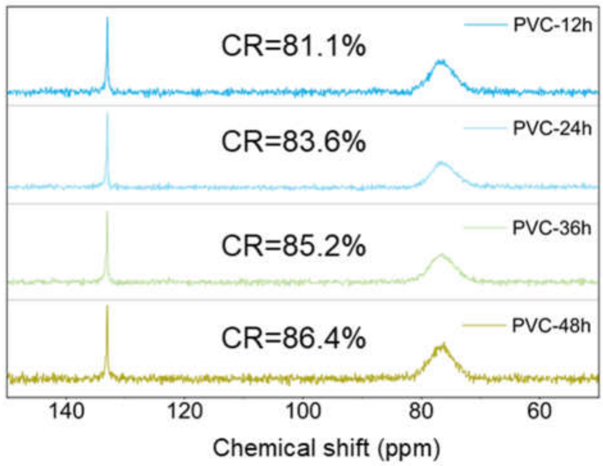

Figure2. (g) ^{13}C NMR spectra of PVC electrolytes with different polymerization time.

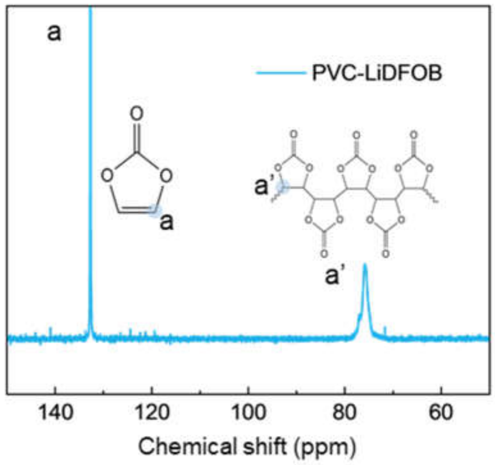

Figure R3. ^{13}C NMR spectrum of LiDFOB-regulated PVC electrolyte.

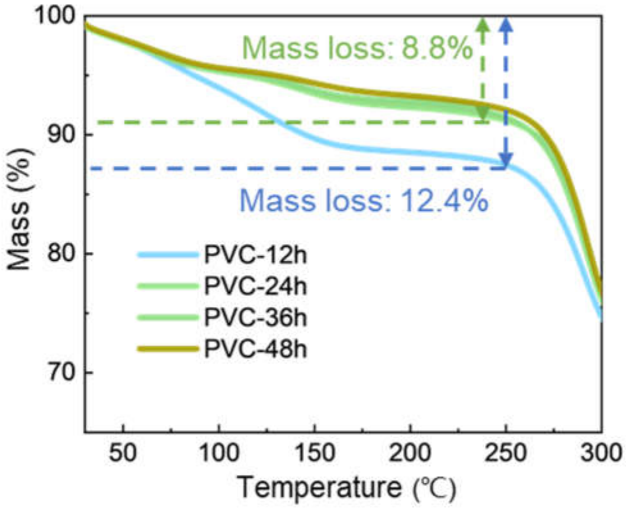

Figure R5. TG curves of various PVC electrolytes with different polymerization time.

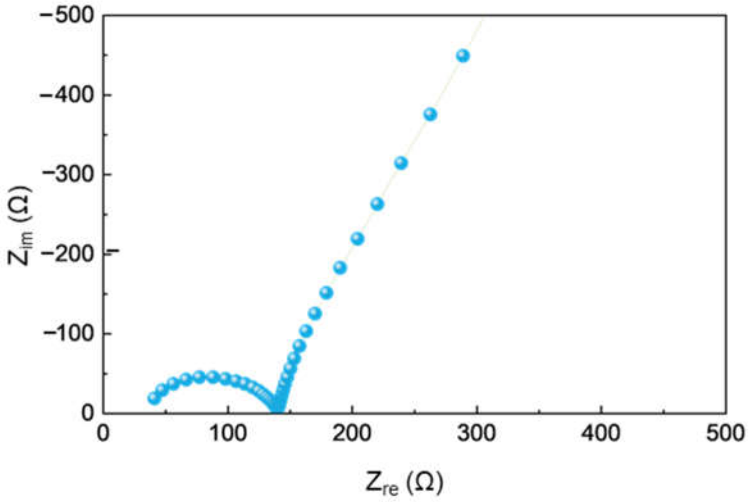

Figure R6. EIS curves of SS//PVC-LiTFSI//SS cell using the annealed PVC electrolyte at 25 ℃.

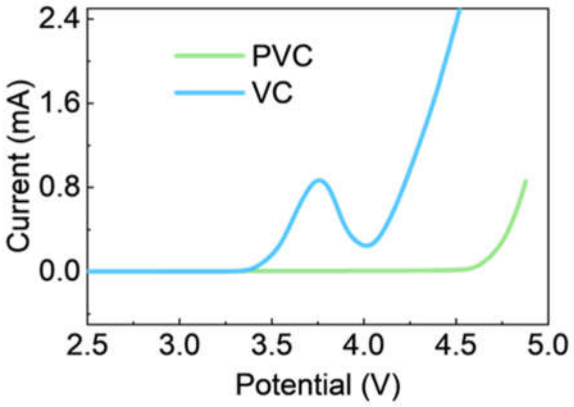

Figure S19. LSV profiles of VC and PVC electrolyte.

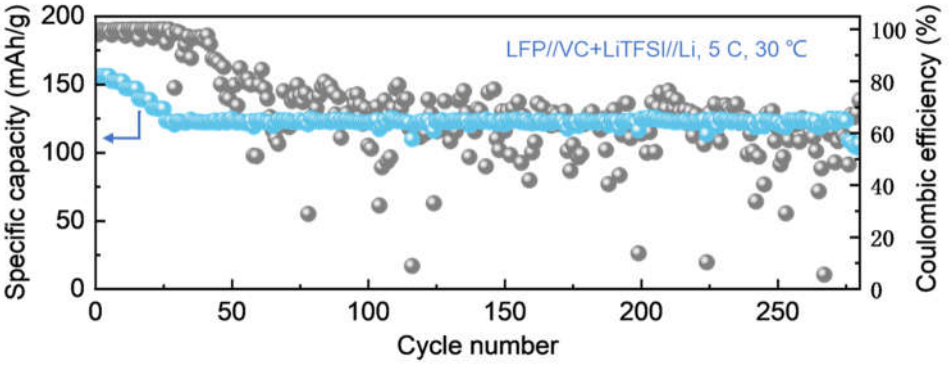

Figure R7. Cycle performance of LFP//VC-LiTFSI//Li cell at 5 C and 30 ℃.

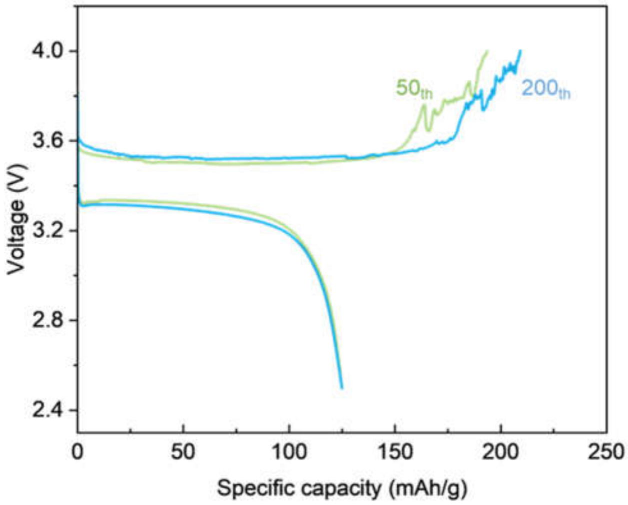

Figure R8. Charge-discharge curves of LFP//VC-LiTFSI//Li cell at 50_{th} and 200_{th} cycles.

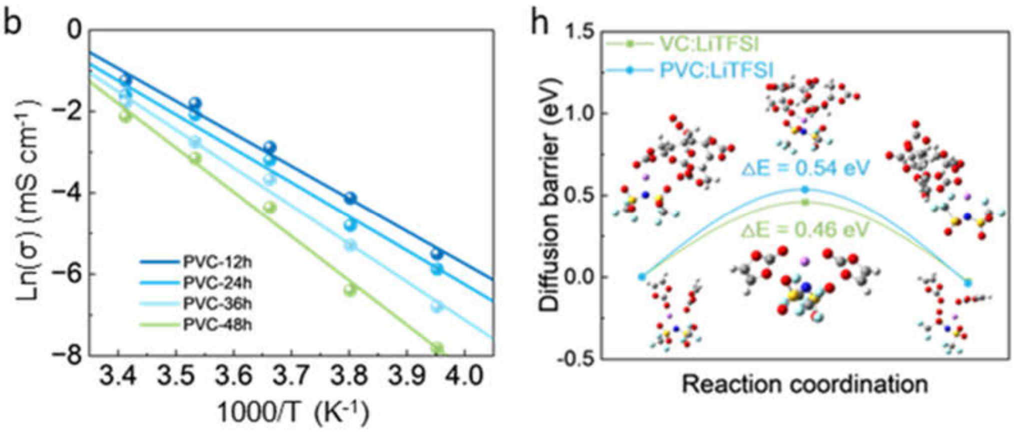

Figure 3. (b) Arrhenius plot at elevated temperatures of PVC electrolytes with different polymerization time. (h) The diffusion barriers of Li between the different coordination structures in polymer phase and oligomer phase.

Reviewer #3-Comment 2: This study controls the dual-phase structure by varying polymerization time and identifies 24 h as the optimal polymerization time. However, the ionic conductivity reported in the Abstract (0.92 mS cm^{-1}) corresponds to PVC-12h, not PVC-24h. Furthermore, it is unclear whether polymerization is fully terminated after 24 h. If polymerization stops, please explain how the stop is controlled. If it does not stop, could further polymerization in a sealed cell lead to continued changes in electrolyte structure and a decrease in ionic conductivity over time?

Response: Thank you very much for your kind suggestions. We apologize for the unclear description of the relationship between ionic conductivity and phase-separated samples in the previous manuscript. Corresponding revision has been made in lines 26-27 in the Abstract.

In our work, the PVC electrolyte was synthesized via thermally initiated in-situ polymerization of VC, the efficiency of which was strongly influenced by temperature. ^{13}C solid-state NMR results indicate that after 12 hours of polymerization, the conversion rate of VC reaches 81.1% (Figure 2g), implying that the majority of highly reactive VC monomers has participated in the polymerization. When the polymerization time was extended to 24 and 48 hours, the conversion rates slightly increase to 83.6% and 86.4%, respectively. The polymerization rate during the later stage is significantly slower than that in the initial 12 hours. This can be attributed to the rapid consumption of highly reactive VC monomers in the early stage, leading to the formation of long-chain polymers of PVC. The subsequent polymerization primarily involved in chain extension between polymer chains, forming higher molecular weight PVC, which is associated with a higher energy barrier that requires elevated temperatures to proceed effectively (Figure S16).

The synthesized PVC-24h electrolyte was further stored and tested at 30 °C, a temperature at which chain extension reactions are hindered effectively, resulting in an extremely slow polymerization rate. EIS measurements conducted after storing various PVC electrolytes at room temperature for 30 days reveal only a slight increase in impedance (Figure R10), indicating significantly suppressed further polymerization reaction and negligible changes in the physicochemical properties of the PVC electrolytes under ambient conditions.

Furthermore, the cycling performance and EIS of the assembled LFP//PVC-24h//Li cells are evaluated at different cycle numbers. As shown in Figure 6f, a small increase in impedance is observed over 1000 cycles, which is mainly associated with interface degradation caused by lithium stripping/plating. And the LFP//PVC-24h//Li cell

maintains a high capacity retention of 94.5% after 1000 cycles (Figure R11), demonstrating that the synthesized PVC-24h electrolyte almost does not undergo further polymerization during battery operation, which could otherwise lead to reduced ionic conductivity and cell capacity fading. The corresponding discussions have been added in lines 25-26 and 213-219 of the revised manuscript.

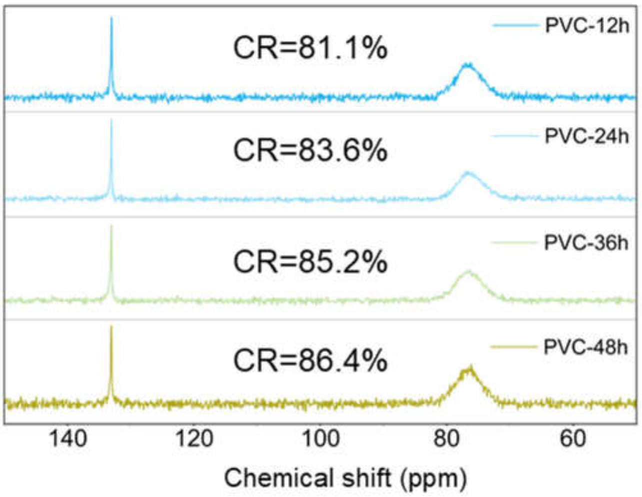

Figure 2. (g) ^{13}C NMR spectra of PVC electrolytes with different polymerization time.

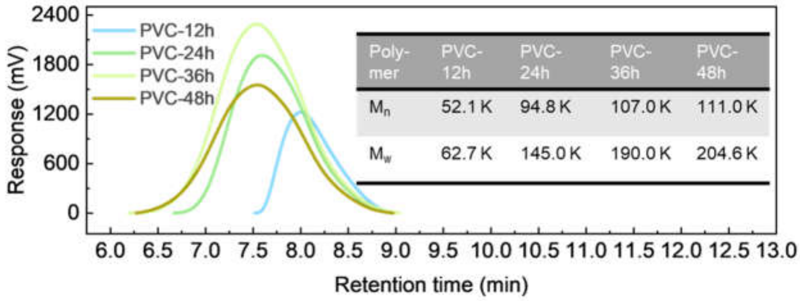

Figure S16. Molecular weights of PVC electrolytes with different polymerization time.

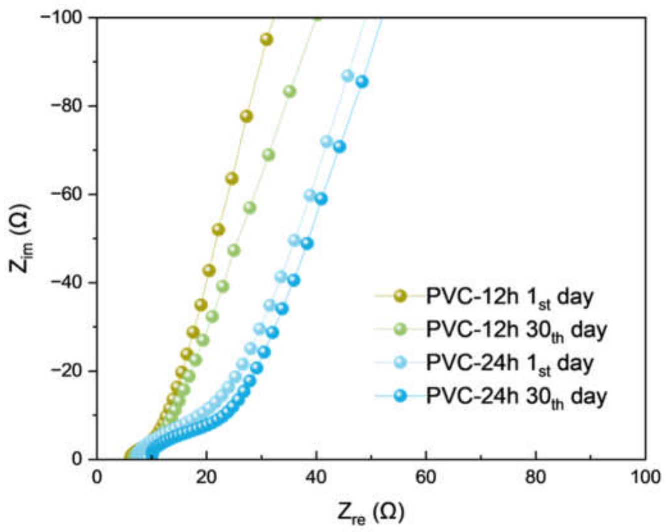

Figure R10. The electrochemical impedance spectra for SS//PVC-12h//SS and SS//PVC-24h//SS cells as the standing time.

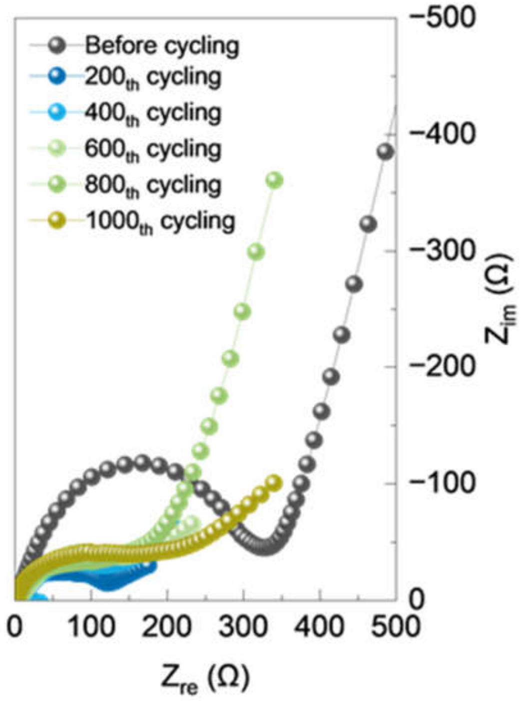

Figure 6. (f) The electrochemical impedance spectra for LFP//PVC-24h//Li cells at different cycles.

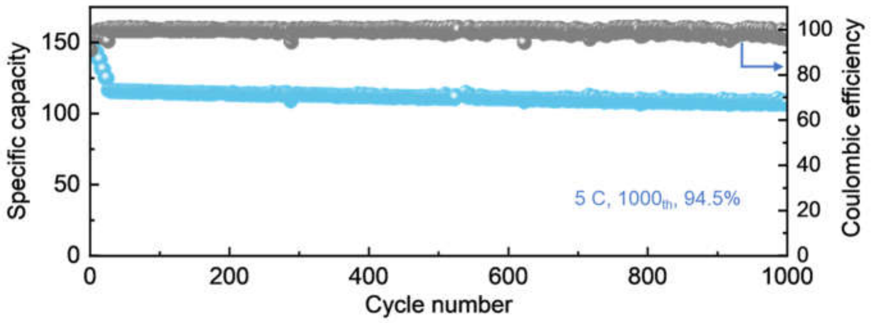

Figure R11. Cycling performance of LFP//PVC-24h//Li full cell at 5 C and 30 ℃.

Reviewer #3-Comment 3: The PVC-24h electrolyte shows excellent long-term cycling performance at high rates (1C, 5C, 10C for over 1000 cycles). However, the interfacial impedance evolution is only provided for up to 200 cycles (Fig. 6f). To better demonstrate interfacial stability during extended cycling, please supplement EIS data after long cycle numbers (e.g., after 500, or 1000 cycles).

Response: Thank you very much for your kind suggestions. To better illustrate the interfacial stability during long-term cycling, the EIS of the assembled LFP//PVC-24h//Li cells have been performed over 1000 cycles at a rate of 5C (Figure 6f). The results demonstrate that the cell show slight impedance increasement even after long-term cycling, corroborating good interfacial stability. The relevant results have been added to lines 486-488 of the revised manuscript.

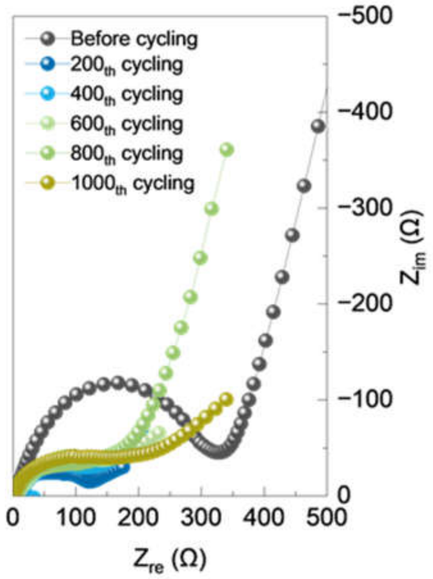

Figure 6. (f) The electrochemical impedance spectra for LFP//PVC-24h//Li cells at different cycles.

Reviewer #3-Comment 4: The PVC-24h electrolyte exhibits high ionic conductivity and Li^+ transference number. However, the Li|PVC-24h|Li symmetric cell shows a relatively high interfacial impedance (Fig. 4e). Please provide a reasonable explanation. Additional characterization on the Li metal/electrolyte interface evolution and rate performance tests in Li|PVC-24h|Li symmetric cells is recommended.

Response: We sincerely appreciate your valuable suggestions. Indeed, the assembled Li|PVC-24h|Li symmetric cell exhibits relatively high impedance before cycling. This is primarily attributed to the use of lithium metal electrodes that are employed as-received without surface polishing to remove the trace impurities (e.g., Li_2O, Li_2CO_3) and potential organic interphase layer on the surface. This conclusion is corroborated by TOF-SIMS analysis of the pristine lithium metal anodes (Figure R11). While, as cycling progresses, the formation of a stable SEI is responsible for a gradual decrease in cell impedance.

Accordingly, the rate capabilities of Li//PVC-24h//Li cells have been evaluated at different current densities (Figure S27). The Li//PVC-24h//Li cell demonstrates stable cycling performance at a current density and deposition capacity of 1 mA cm^{-2} and 1 mAh cm^{-2}, where only minor voltage fluctuation can be observed. This improved stability is attributed to the high mechanical strength of the polymer phase in the phase-separated PVC electrolyte, showing that the PVC-24h electrolyte with high ionic conductivity and t_{Li^+} can induce uniform lithium deposition and ensure good compatibility with lithium anode.

To further elucidate the interfacial evolution, the cycled lithium metal anodes have also been characterized by TOF-SIMS (Figure 6g,h) and XPS (Figure R12). It can be seen that the cycled lithium anode matched with the PVC-based electrolyte exhibits a thinner SEI with reduced organic content on the surface. This indicates that the anion-polymer interaction in the electrolyte lowers the desolvation energy barrier of the anions, promoting their participation in interfacial reactions to form an inorganic-rich SEI layer. Consequently, this process mitigates further side reactions between the electrolyte and the lithium metal anode. The relevant discussions have been added to lines 383-389 and 486-500 of the revised manuscript.

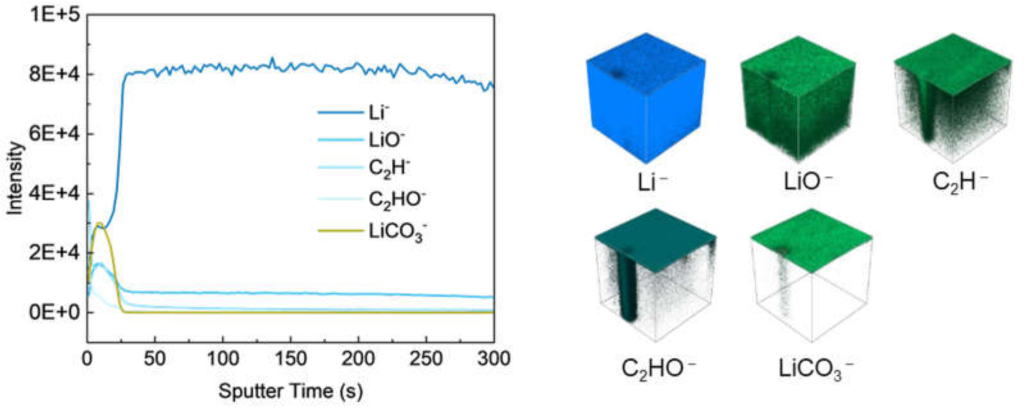

Figure R11. (a) The intensity evolution and (b) 3D renders of Li^-, $Li_2F_3^-$ and $Li_2CO_3^-$ in the TOF-SIMS sputtered volumes of pristine lithium metal.

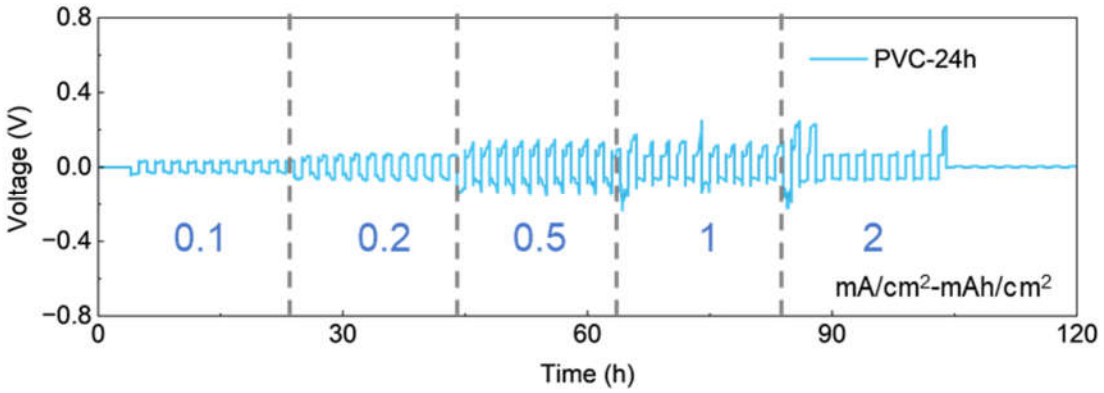

Figure S27. Rate performance of Li//Li symmetric batteries using PVC-24h electrolyte.

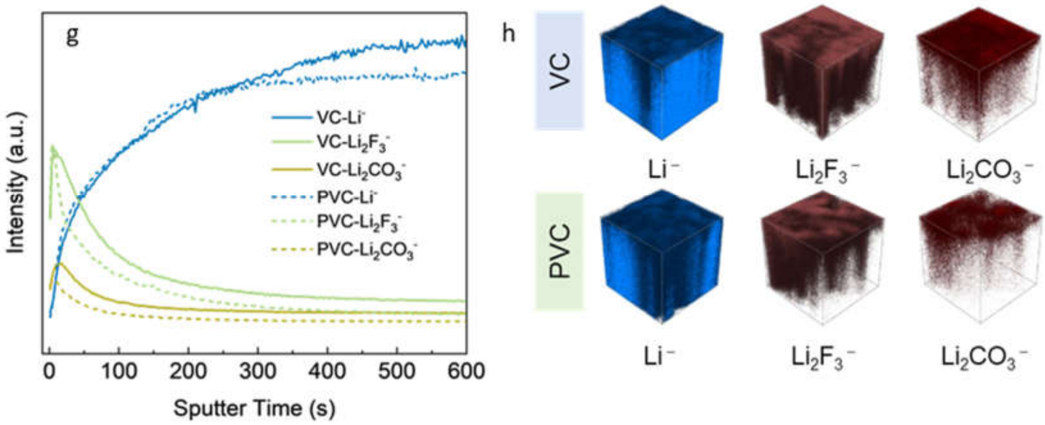

Figure 6. (g) The intensity evolution and (h) 3D renders of Li^-, $Li_2F_3^-$ and $Li_2CO_3^-$ in the TOF-SIMS sputtered volumes of VC-LiTFSI and PVC-LiTFSI induced SEI on the cycled lithium metal anodes.

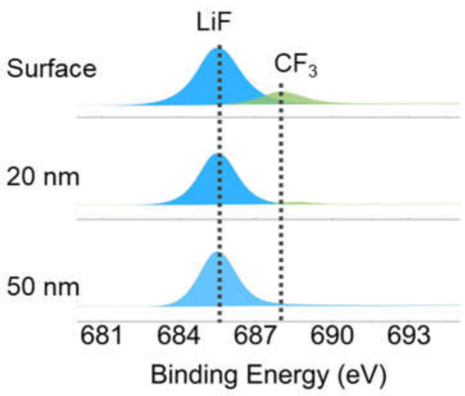

Figure R12. The F 1s XPS spectra of lithium metal anode at varying etching depths after 200 cycles at 1 C in LFP//PVC-24h//Li cells.

Reviewer #3-Comment 5: In this work, the polymer phase is the critical factor in improving the intrinsic oxidation stability of the electrolyte. Given the high oxidation stability reported (~4.5 V for PVC-24h), whether the PVC-24h electrolyte could be applied in high-voltage systems such as NCM-based batteries? A preliminary data and brief discussion on compatibility with higher-voltage cathodes (e.g., NCM811 or NCM622) would significantly strengthen the practical relevance of this work.

Response: Thanks very much for your professional insights. The NCM//PVC-24h//Li and LCO//PVC-24h//Li full cells have been assembled accordingly and tested in 2.8–4.4 V at 1 C. As shown in Figure R13a and R13c, the NCM//PVC-24h//Li full cell exhibits high specific capacity of 171.8 mAh/g at 1C with a superior capacity retention of 96.5% after 50 cycles at 30 °C. Besides, the LCO//PVC-24h//Li full cell exhibits high specific capacity of 152.7 mAh/g at 1C with a superior capacity retention of 96.9% after 50 cycles at 30 °C (Figure R13b), and the corresponding charge–discharge curves at the 1st and 50th (Figure R13d) cycles possess a minimal polarization and a high degree of overlap. The above results suggest good compatibility of the produced PVC electrolyte with high-voltage cathodes. Additionally, the current PVC-based electrolyte formulation does not incorporate additional additives. It is proposed that introducing small quantities of functional additives—such as FEC, LiDFOB, or LiBOB—benefits to further enhance its electrochemical stability under high-voltage conditions.

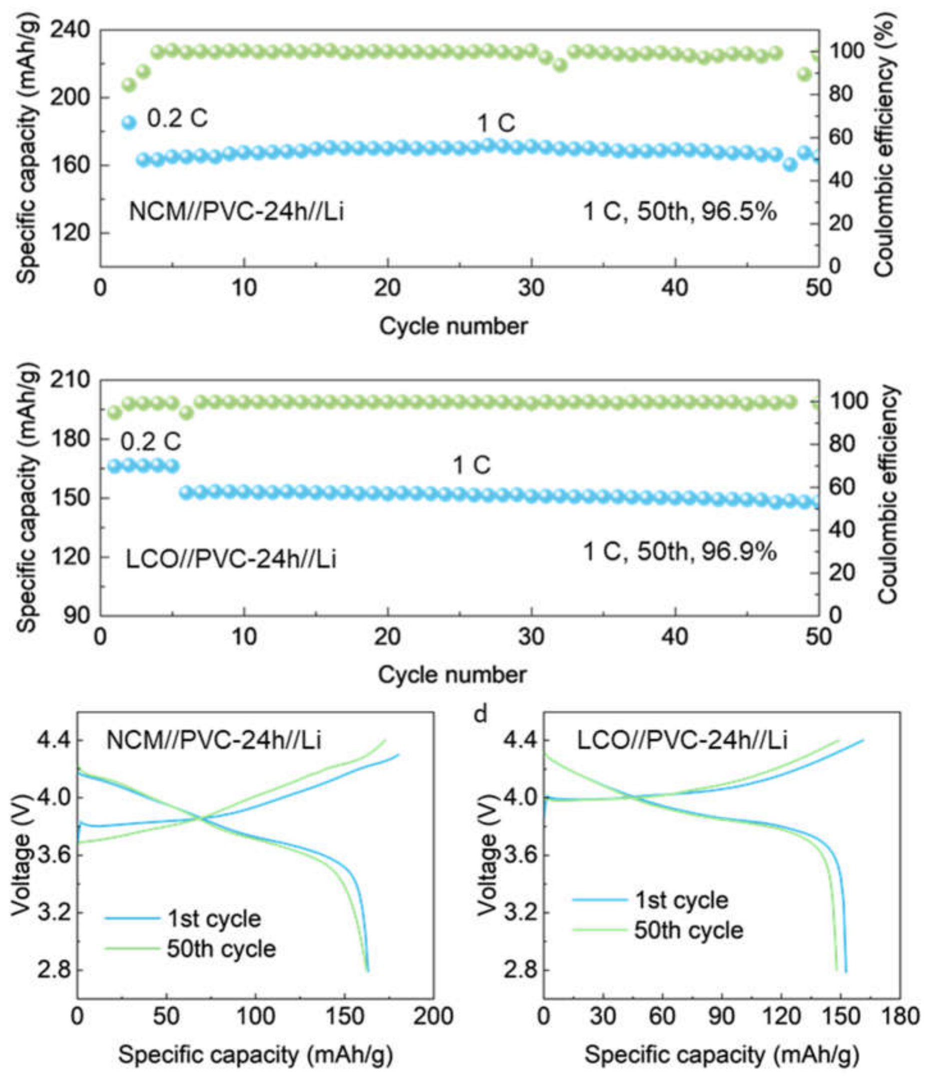

Figure R13. Cycle performance of (a) NCM//PVC-24h//Li and (b) LCO//PVC-24h//Li full cells at 1 C and 30 °C in 2.8–4.4 V. And the charge-discharge curves at 1st and 50th cycles of (c) NCM//PVC-24h//Li and (d) LCO//PVC-24h//Li full cells at 1 C and 30 °C.

Response to the Reviewer #4

General comment: In this manuscript, the authors describe a process to prepare poly(vinyl carbonate) (PVC) solid polymer electrolytes through a polymerization induced phase separation technique in vinylene carbonate. The resulting PVC is reported to have excellent electrochemical properties for application in solid-state lithium metal batteries. However, because this manuscript is motivated by the development of polymeric materials for solid-state lithium metal batteries, I believe it should be rejected as the authors do not convincingly show that the polymeric and/or oligomeric phase in the phase-separated PVC is primarily responsible for the transport and the energy storage performance. Rather, the NMR, Raman, and TGA data in Figures 2f, 2g, and 2j, respectively, suggest that substantial unreacted monomer, vinylene carbonate (VC), remains. The authors report a monomer conversion as high as only 65%, meaning 35% of the system is liquid. It is likely that residual unreacted VC is predominantly responsible for lithium transport and interfacial reactivity. It will be important to elucidate the relative roles of polymer, oligomer, and unreacted monomer in the system. Moreover, I don't think the novelty of this study warrants publication in Nature Communications, as several papers have reported on the in-situ polymerization of polymer-in-liquid systems or gel electrolytes with good energy storage performance (cf. 10.1016/j.xcrp.2020.100146 , 10.1002/advs.201600377, 10.1002/aenm.202300798,)

Reviewer #4-Comment 1: This manuscript is motivated by the development of polymeric materials for solid-state lithium metal batteries, but the authors do not convincingly show that the polymeric and/or oligomeric phase in the phase-separated PVC is primarily responsible for the transport and the energy storage performance.

***Response*:** Thanks very much for your constructive comments. We are very sorry that the previous incomplete description of unreacted monomers may have caused concern. We have carried out additional experiments to clarify this point more clearly. In this work, unreacted monomer is categorized as part of the oligomer phase. This is because the oligomer phase primarily consists of molecules that become less reactive in the later stages of polymerization. Their polymerization rate is significantly slow, allowing them to form a continuous, highly amorphous phase around the precipitated polymer phase during the polymerization stage.

In order to demonstrate the relationship between the phase-separated structure and the optimization of ion transport efficiency, PVC electrolytes regulated by different lithium salts are used to clarify the role of residual monomers in different electrolyte structures.

As illustrated in Figure S4, the in-situ polymerization of VC exhibits divergent polymeric architecture with different conditions. Macroscopically, the polymerization process regulated by LiTFSI shows evident precipitation of solid particles, with the solution turning from transparent to turbid. In contrast, the LiDFOB-mediated polymerization system consistently remains clear (Figure R2). Moreover, significant differences are observed in their microstructures: the LiTFSI-based electrolyte displays clear phase-separated morphology, featuring well-defined boundaries between different phases in both SEM and TEM images (Figure 1b and Figure 2a). Conversely, electrolytes incorporating with LiDFOB exhibit a homogeneous bulk structure without phase separation (Figure S5). Furthermore, NMR analysis has been conducted to determine the conversion rate of the LiDFOB-containing electrolyte. The calculated conversion rate is only 44.5%, which is notably lower than that of the LiTFSI-regulated systems (Figure R3). Despite its significantly lower conversion rate, the PVC-LiDFOB electrolyte does not exhibit phase separation phenomenon, and the electrochemical impedance spectroscopy (EIS) of assembled SS//PVC-LiDFOB//SS cell is significantly higher than that with the phase-separated PVC-LiTFSI electrolyte (Figure R4). This indicates that under partial conversion conditions, the unreacted oligomers do not spontaneously segregate around the polymer zones to form a phase-separated structure. Instead, they disperse uniformly within the overall polymer phase, resulting in only a limited enhancement in ionic conductivity. In contrast, the LiTFSI-mediated electrolyte demonstrates clear phase-separation characteristics and a substantially improved ionic conductivity. Additionally, the phase-separated PVC electrolyte was annealed at 200°C for 5 hours to allow full evaporation of residual monomer. The assembled SS//PVC-LiTFSI//SS cell using the annealed electrolyte still remains lower impedance than that using the PVC-LiDFOB electrolyte (Figure R6). This confirms that the phase-separated structure substantially enhances the ionic conductivity of the PVC electrolyte. Furthermore, the electrochemical performance of the un-polymerized precursor under identical conditions have been evaluated. LSV shows the oxidation current of VC electrolyte increases significantly at 3.3 V (Figure S19). At a current density of 5C, the coulombic efficiency of the assembled LFP//VC-LiTFSI//Li cell decreases significantly (Figure R7), accompanied by evident electrolyte decomposition (Figure R8). These results indicate that the unreacted monomer does not improve electrolyte performance; conversely, its poor electrochemical stability leads to a reduction in coulombic efficiency. In conclusion, the design of phase-separated structure of PVC-based electrolyte in our work significantly enhances the overall electrochemical stability and ion conductivity.

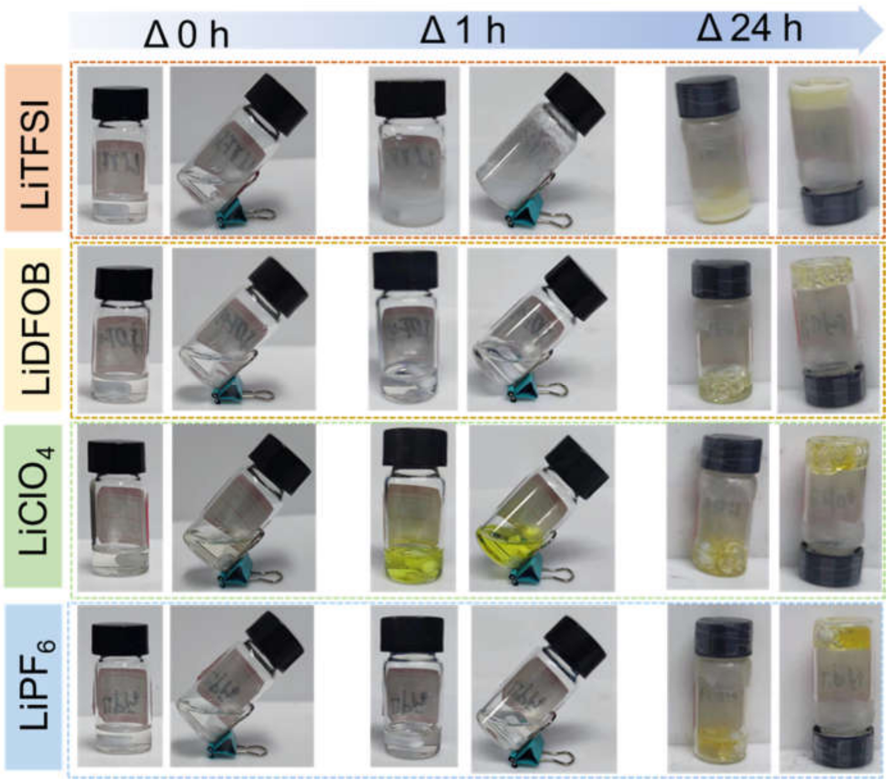

Figure S4. Optical images of PVC electrolytes regulated by different lithium salts at different polymerization time.

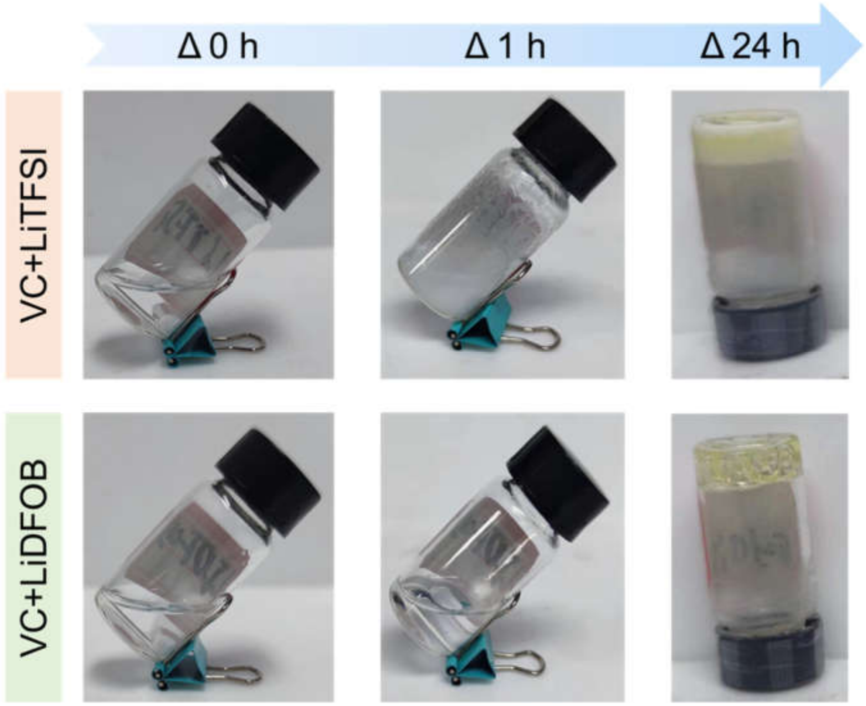

Figure R2. Optical images of LiDFOB-regulated and LiTFSI-regulated PVC electrolytes before and after polymerization at different time.

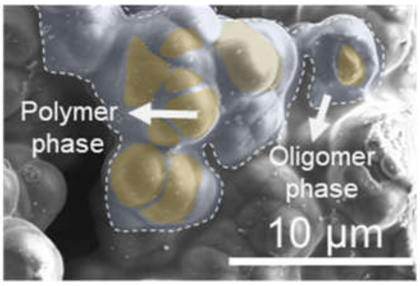

Figure 1. (b) SEM image of LiTFSI-regulated PVC electrolyte after in-situ polymerization.

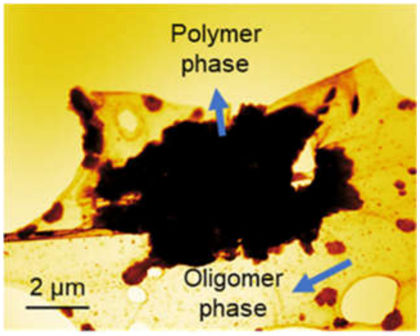

Figure 2. (a) High-resolution TEM image of PVC-24h electrolyte.

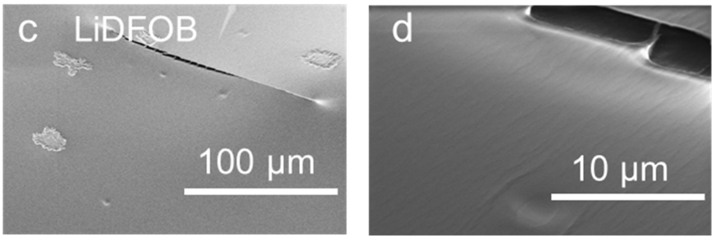

Figure S5. (c-d) SEM images of the produced PVC electrolyte with LiDFOB after in-situ polymerization.

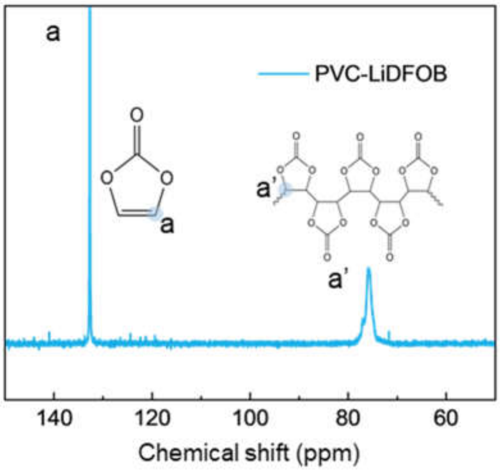

Figure R3. ^{13}C NMR spectrum of LiDFOB-regulated PVC electrolyte.

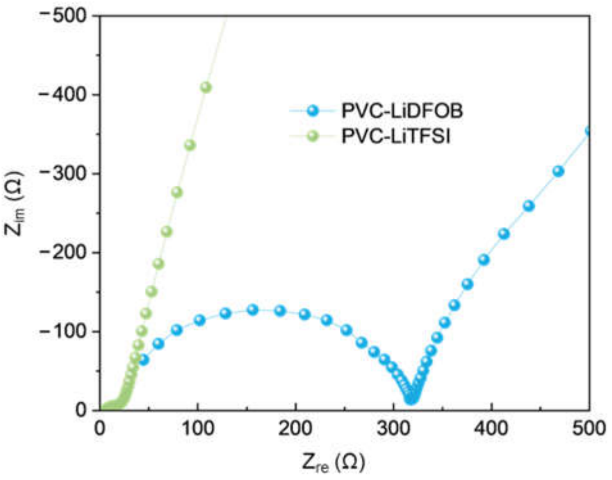

Figure R4. EIS curves of SS//PVC-LiDFOB//SS and SS//PVC-LiTFSI//SS cells at 25 ℃.

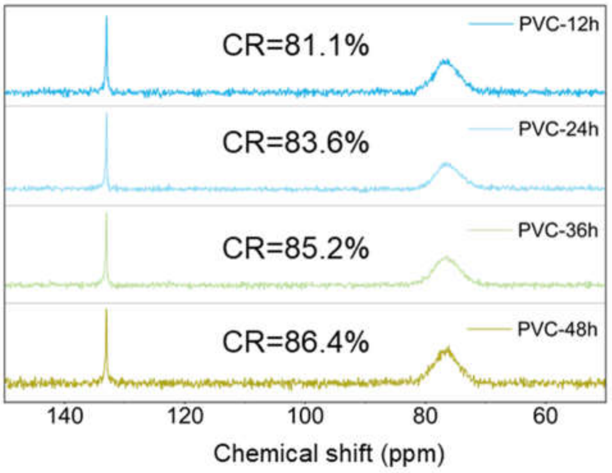

Figure2. (g) ^{13}C NMR spectra of PVC electrolytes with different polymerization time.

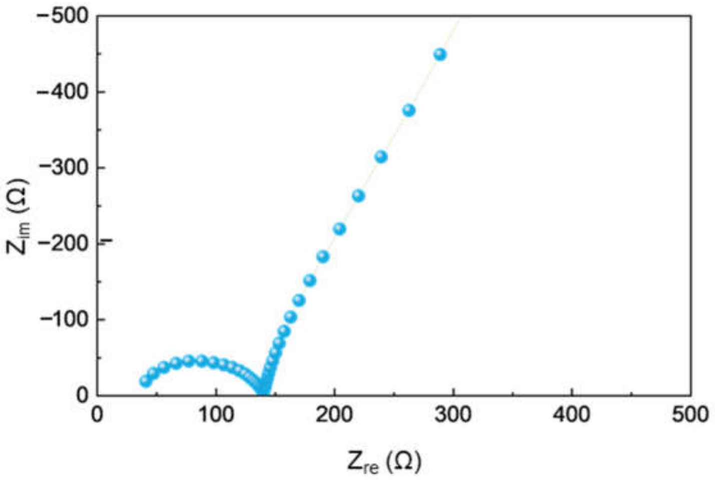

Figure R6. EIS curve of SS//PVC-LiTFSI//SS cell using the annealed PVC electrolyte at 25 ℃.

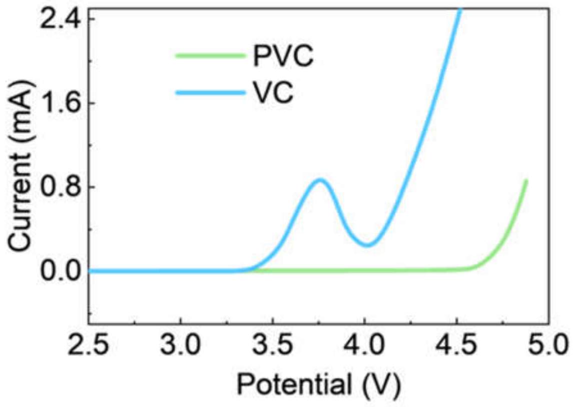

Figure S19. LSV profiles of VC and PVC electrolytes.

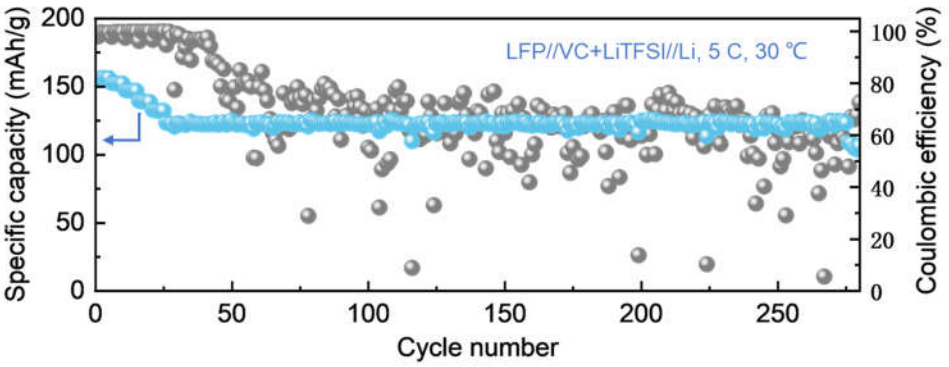

Figure R7. Cycle performance of LFP//VC-LiTFSI//Li cells at 5 C and 30 ℃.

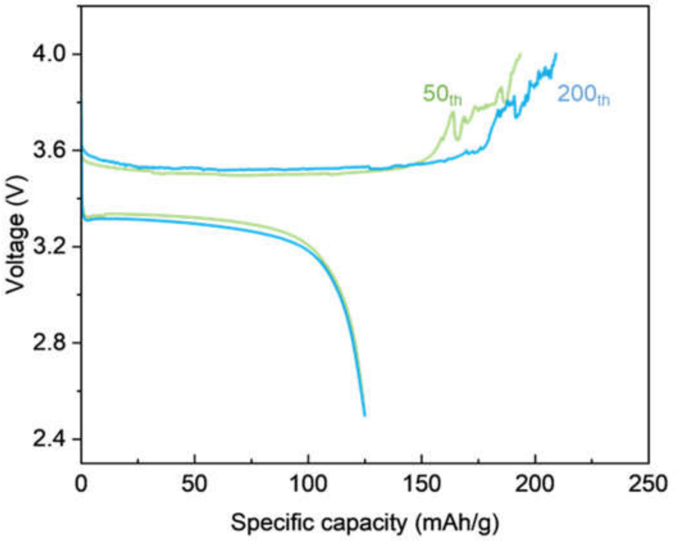

Figure R8. Charge-discharge curves of LFP//VC-LiTFSI//Li cells at 50_{th} and 200_{th} cycles.

Reviewer #4-Comment 2: Rather, the NMR, Raman, and TGA data in Figures 2f, 2g, and 2j, respectively, suggest that substantial unreacted monomer, vinylene carbonate (VC), remains. The authors report a monomer conversion as high as only 65%, meaning 35% of the system is liquid. It is likely that residual unreacted VC is predominantly responsible for lithium transport and interfacial reactivity.

Response: Thanks very much for your kind suggestions. We apologize for the deviations in calculating the conversion rate of the PVC-based electrolyte based on the indirectly related C=O groups. Accordingly, we have recalculated the conversion rate using the more appropriate peak corresponding to the C=C bond in the solid-state ^{13}C NMR spectra (Figure 2g). The conversion rate for the PVC-24h electrolyte is about 83.6%. This indicates that the combined mass fraction of oligomers and unreacted monomer in the electrolyte is 16.4%. In addition, thermogravimetric analysis reveals a mass loss of only 8.8% before 250 °C for the PVC-24h electrolyte (Figure R5). Notably, even the PVC-12h electrolyte, which possesses the highest oligomer content, exhibits a minimal thermal mass loss of merely 12.4% up to 250 °C. These results confirm the relatively low content of oligomers and residual monomers within the produced various PVC-electrolytes. In contrast, the previously reported PIPS (polymer-induced phase separation) electrolytes typically contain a liquid phase exceeding 40% (*Adv. Energy Mater.*, 2024, 14, 22,2400428; *Nat. Commun.*, 2023, 14, 2301; *Nature*, 2022, 601, 217). Therefore, our single-solvent-induced phase-separation strategy demonstrates a lower oligomer content alongside better electrochemical performance.

To analyze the impact of monomer residue on electrochemical performance, the EIS of PVC-LiDFOB electrolyte with low conversion rate has been tested. As shown in Figure R4, the impedance of cell using such a non-phase-separated electrolyte is significantly higher than that using the phase-separated PVC electrolyte. As mentioned previously (response to comment 1), the cell using the annealed PVC-LiTFSI electrolyte remains lower impedance than that using the PVC-LiDFOB electrolyte (Figure R6), confirming that the phase-separated structure substantially enhances the ionic conductivity of the electrolyte. In addition, the electrochemical investigations indicate that although the unreacted monomer possesses a lower migration energy barrier (Figure 3h), it does not improve electrolyte performance; conversely, its poor electrochemical stability always leads to a reduction in coulombic efficiency (Figures S19, R7 and R8). In conclusion, the design of the phase-separated structure enhances the ion conductivity and electrochemical stability of the PVC electrolyte simultaneously.

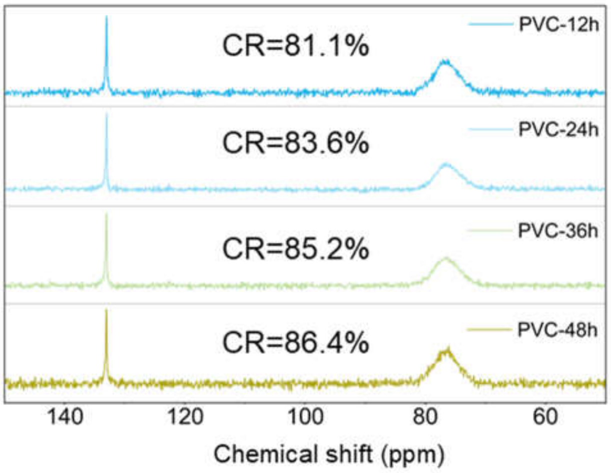

Figure 2. (g) ^{13}C NMR spectra of PVC electrolytes with different polymerization time.

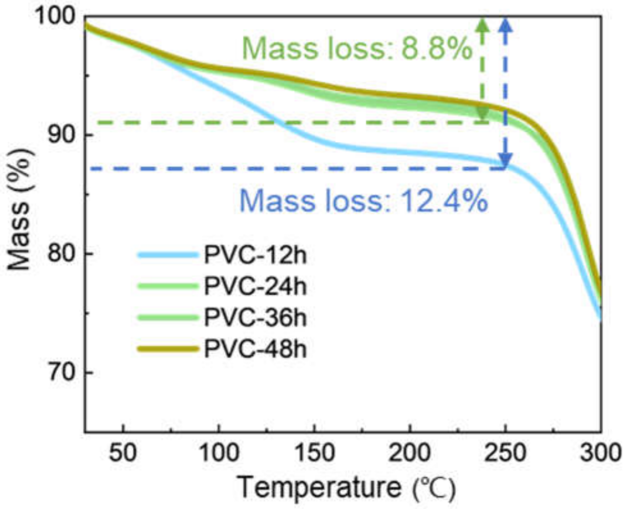

Figure R5. TG curves of various PVC electrolytes with different polymerization time.

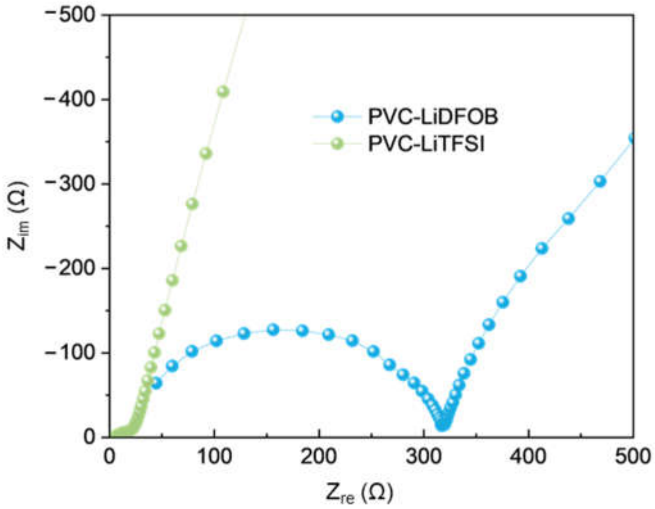

Figure R4. EIS curves of SS//PVC-LiDFOB//SS and SS//PVC-LiTFSI//SS cells at 25 ℃.

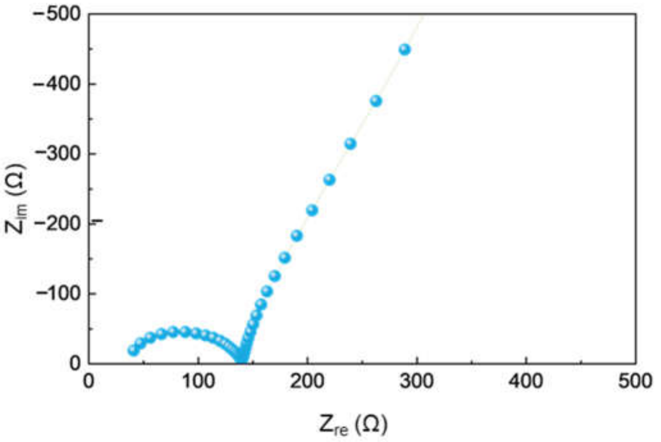

Figure R6. EIS curve of SS//PVC-LiTFSI//SS cell using the annealed PVC electrolyte at 25 ℃.

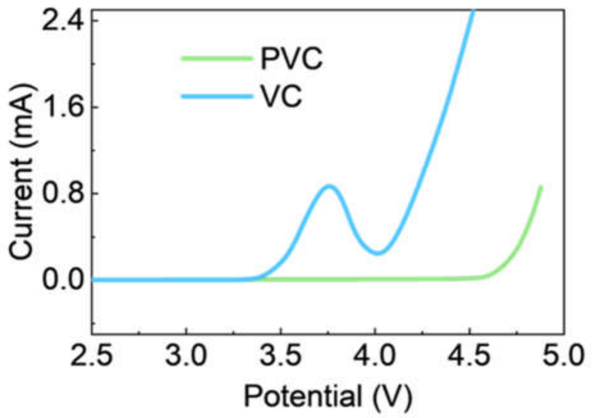

Figure S19. LSV profiles of VC and PVC electrolyte.

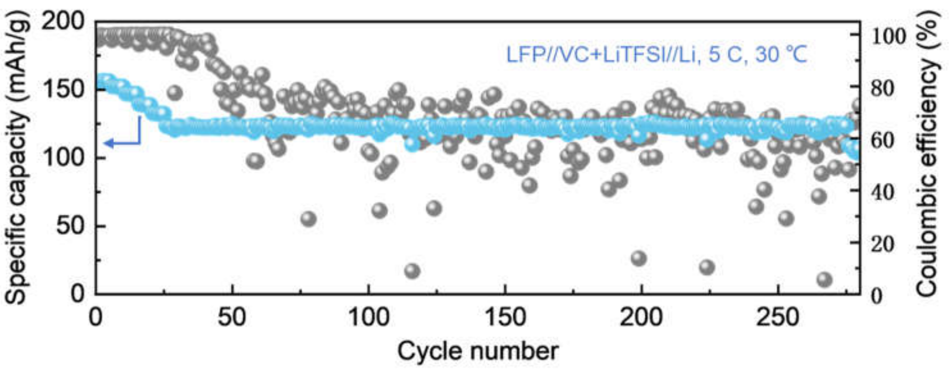

Figure R7. Cycle performance of LFP//VC-LiTFSI//Li cell at 5 C and 30 ℃.

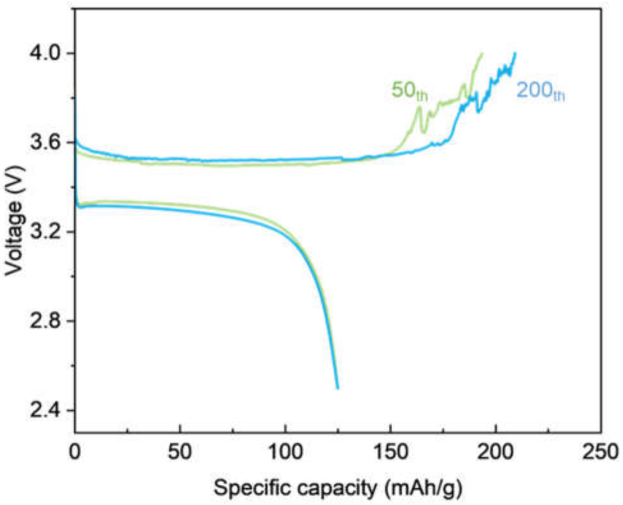

Figure R8. Charge-discharge curves of LFP//VC-LiTFSI//Li cells at 50_{th} and 200_{th} cycles.

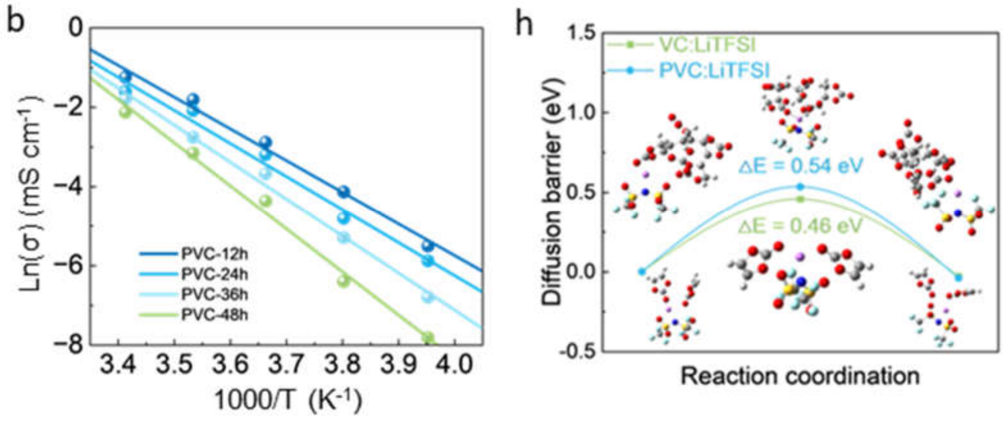

Figure 3. (b) Arrhenius plot at elevated temperatures of PVC electrolytes with different polymerization time. (h) The diffusion barriers of Li between the different coordination structures in polymer phase and oligomer phase.

Reviewer #4-Comment 3: It will be important to elucidate the relative roles of polymer, oligomer, and unreacted monomer in the system.

***Response*:** Thanks very much for your valuable suggestions. Undoubtedly, clarifying the relationship between polymers, oligomers, and unreacted monomers is of significant importance. In our work, the unreacted monomers are classify as part of the oligomer phase. This is because these small molecules possess low migration barriers (Figure 3h) and activation energies (Figure 3b), which endow the electrolyte with rapid ion transport ability. However, these small molecules exhibit poor electrochemical stability (Figure S19) and mechanical strength, making them prone to oxidation at the cathode and limiting

their inhibitory effect on the potential growth of lithium dendrite.

The presence of the polymer phase compensates for the shortcomings of the oligomer phase. Polymers possess higher mechanical strength (Figure S20), providing a degree of resistance to lithium dendrite penetration (Figure S27). Furthermore, the polymer phase exhibits greater electrochemical stability (Figure 3c). Importantly, the weak hydrogen-bonding interaction between the polymer phase and anions restricts anion mobility within the PVC electrolyte, thereby increasing the lithium-ion transference number and enhancing overall ion transport efficiency (Figure S8). Concurrently, this interaction between the polymer phase and anions modulates the solvation structure of lithium ions, resulting in a lower desolvation energy barrier for $TFSI^-$ (Figure R9). This promotes their participation in the formation of stable CEI (Figure 6a-c) and SEI (Figure 6g,h) layers, suppressing further electrolyte decomposition.

In conclusion, the oligomer phase facilitates rapid ion conduction, while the continuous polymer matrix provides mechanical robustness and interfacial stability, creating a synergistic effect essential for high-performance electrolytes. As a result, the unique advantages of this elaborately designed phase-separated structure enable PVC electrolytes to deliver outstanding electrochemical performance.

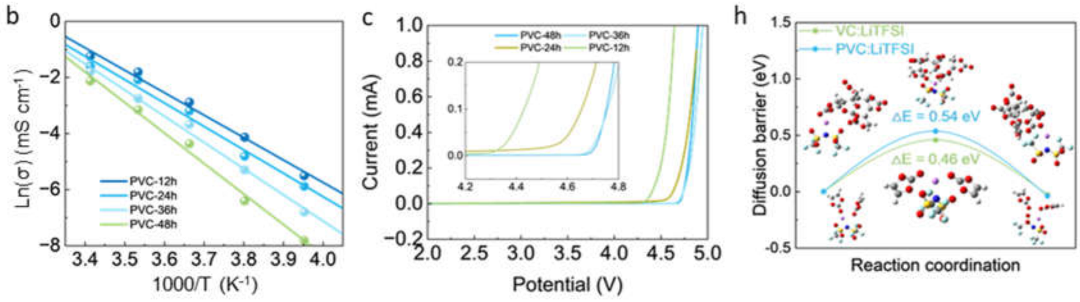

Figure 3. (b) Arrhenius plot at elevated temperatures and (c) LSV profiles of PVC electrolyte with different polymerization time. (h) The diffusion barriers of Li between the different coordination structures in polymer phase and oligomer phase.

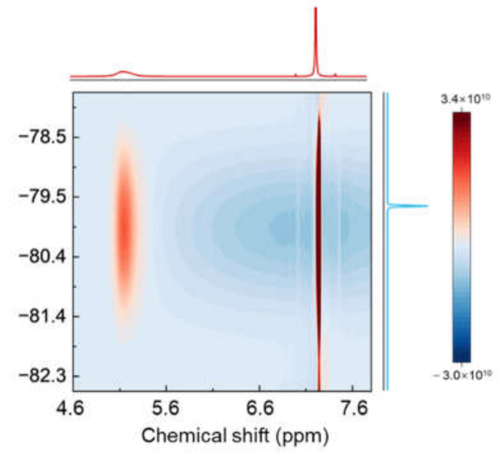

Figure S8. 2D ^{1}H–^{19}F HETCOR NMR spectrum of PVC-24h electrolyte.

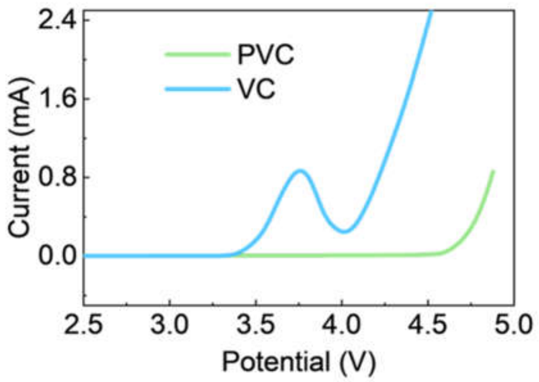

Figure S19. LSV profiles of VC and PVC electrolytes.

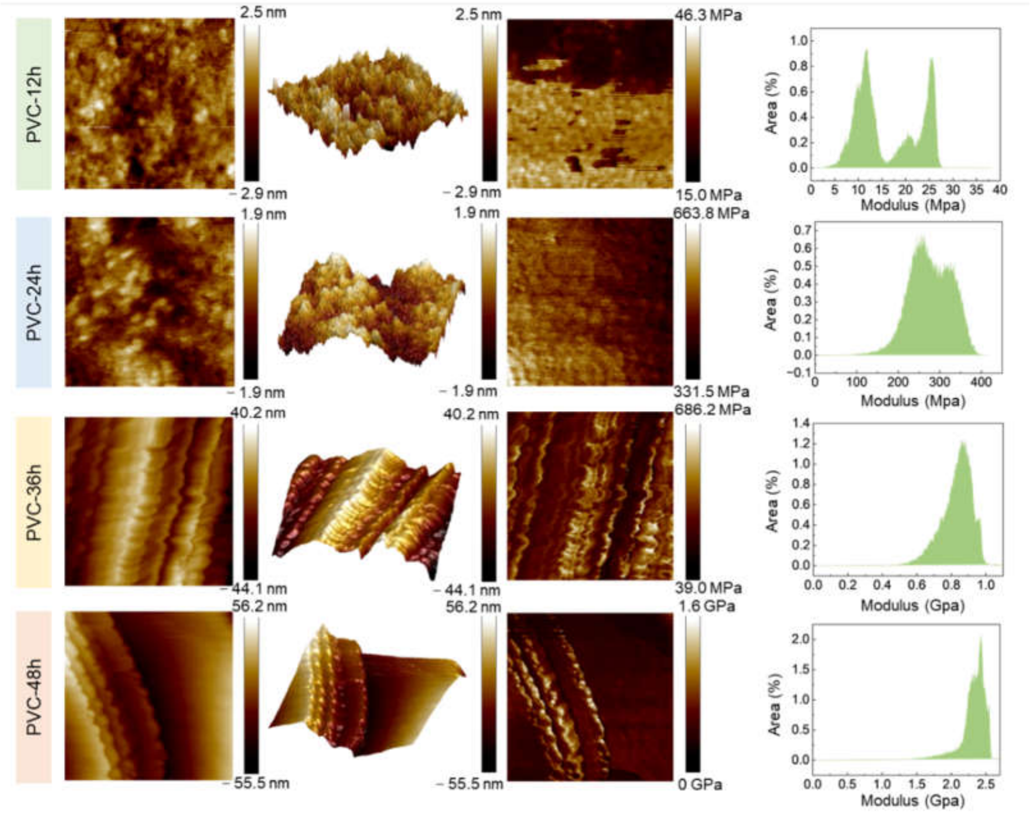

Figure S20. 2D, 3D AFM images and 2D Young's modulus distribution range of the produced PVC electrolytes with different polymerization time.

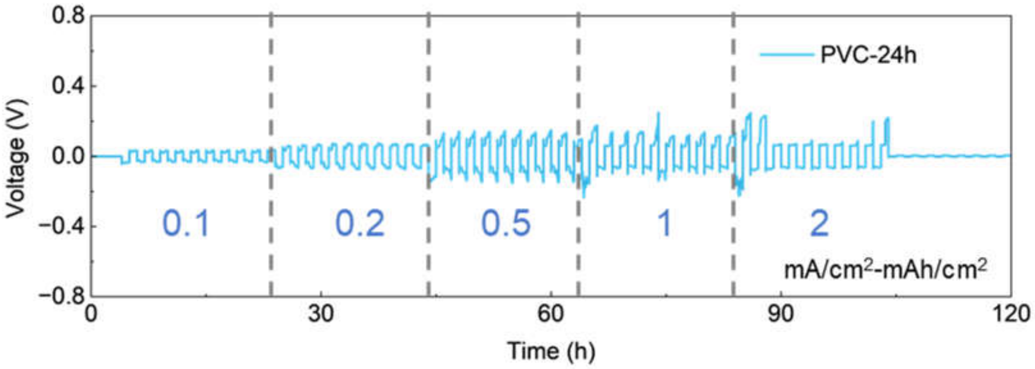

Figure 27. Rate performance of Li//Li symmetric batteries using PVC-24h electrolyte.

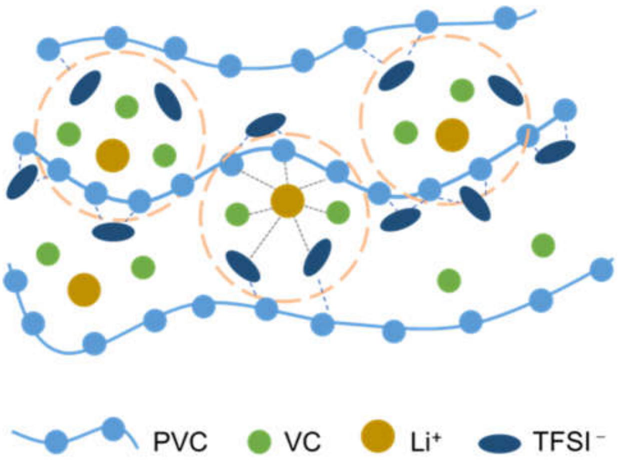

Figure R9. Illustration of the configuration of ionic solvates of PVC electrolyte.

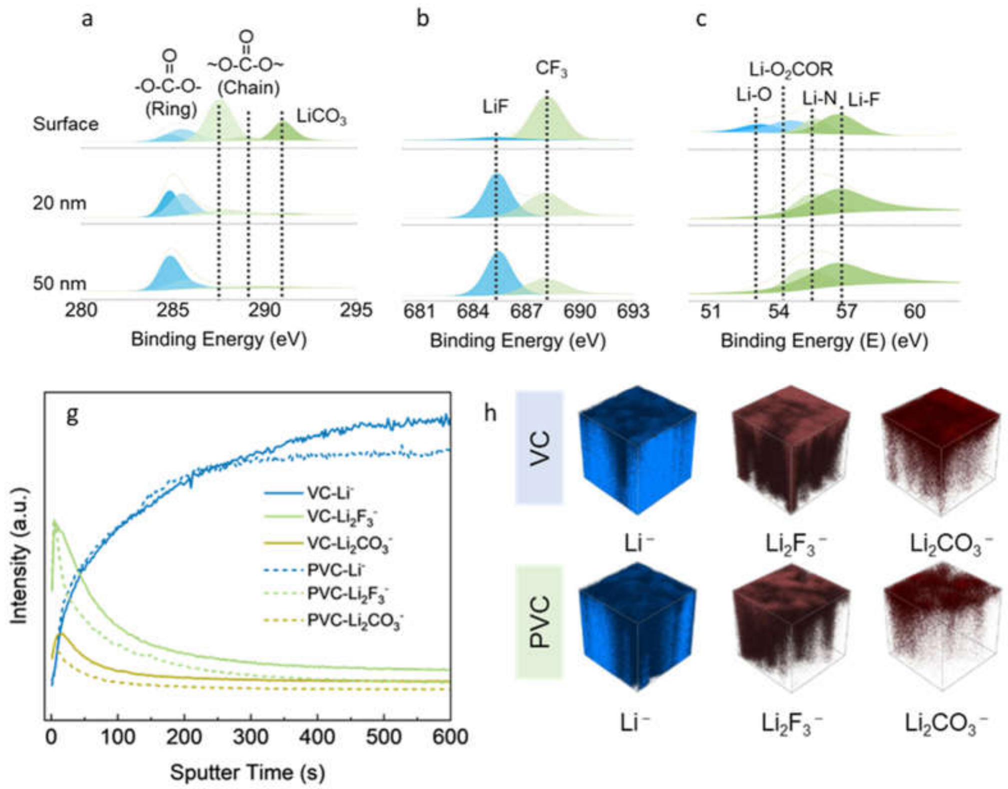

Figure 6. The (a) C 1s, (b) F 1s, and (c) Li 1s XPS spectra of LFP at varying etching depths after 200 cycles at 1 C in LFP//PVC-24h//Li cells. (g) The intensity evolution and (h) 3D renders of Li^-, $Li_2F_3^-$ and $Li_2CO_3^-$ in the TOF-SIMS sputtered volumes of VC-LiTFSI and PVC-LiTFSI induced SEI on cycled lithium anode.

Reviewer #4-Comment 4: Several papers have reported on the in situ polymerization of polymer-in-liquid systems or gel electrolytes with good energy storage performance

(cf.10.1016/j.xcrp.2020.100146, 10.1002/advs.201600377, 10.1002/aenm.202300798).

Response: Thanks very much for your comments. The referenced studies in your comments represent significant advances in electrolyte synthesis, particularly in combining solvents like ethylene carbonate (EC) and 1,3-dioxolane (DOL) for in-situ polymerization, as well as in developing in-situ polymerized poly(vinylene carbonate) (PVC)-based electrolytes. These works convincingly demonstrate the effectiveness of in-situ polymerization for synthesizing advanced solid-state electrolytes, with a primary focus on synthesis methodologies and their application. However, they do not involve the discovery and discussions about phase transformation process during polymerization process and the subsequent structural control of the resulting polymer electrolytes, which critically determine the electrochemical performance. In contrast, our work innovatively integrates a phase-separation structure with the in-situ polymerization process of single-solvent system.

Here we would highlight the superiority of our proposed strategy again. Phase separation is an effective way to increase the ionic conductivity and interface stability of SPEs. However, conventional polymerization-induced phase separation (PIPS) strategies enhance ion transport yet rely on external components such as deep eutectic solvents or ionic liquids, increasing cost and complexity, Moreover, PIPS is difficult to precisely control, particularly in terms of morphology and domain distribution, leading to poor reproducibility and limited scalability. Herein, a LiTFSI-mediated in-situ polymerization strategy is developed to induce controllable phase separation in a PVC electrolyte using a single solvent. Electrostatic interactions between lithium salts and the polymer drive self-organized dual phases that combine mechanical robustness with efficient ion transport. By simply varying the polymerization time, the polymerization degree and phase-separated morphology of electrolyte can be controlled precisely, and the mechanism underlying the anion-induced polymer phase separation—an aspect seldom addressed in previous studies, is elucidated in detail. Such a self-organized dual phases model endows the PVC SPEs with a tunable ionic conductivity from 0.20 to 0.92 mS/cm at room temperature and outstanding lithium ion transference number of 0.78. As a result, the assembled Li//PVC//Li symmetric cells demonstrate stable lithium stripping/plating behavior for up to 6000 hours at 30 °C. When applied in $LiFePO_4$//PVC//Li full cells, a discharge capacity of 121.4 mAh/g is achieved at 5 C and 30 °C with 90% capacity retention after 4000 cycles. The proposed anion-polymer interaction mechanism in this work offers a feasible solution to design advanced SPEs for high-rate solid-state lithium metal batteries at room temperature.

Responses to the Reviewers' comments and the Revision details

Manuscript ID: NCOMMS-25-94149A

Title: Tailoring electrolyte phase separation for high-rate solid-state lithium metal batteries

Journal: Nature Communications

Thanks very much for the reviewers' patient review and valuable comments with a clear goal to make the manuscript better. We have carefully revised our manuscript by taking full consideration of the reviewers' comments and made targeted revisions to this work. The point-by-point responses to the reviewers' comments and the revision details are as follows:

Response to comments from the Reviewer # 1:

General comment: The authors have thoroughly addressed this reviewer's concerns, and the manuscript has been significantly improved as a result. I now recommend its acceptance without further revision.

Response: Thanks very much for your professional insights and recognition. Your feedback has greatly contributed to the improvement of our manuscript.

Response to comments from the Reviewer # 2:

General comment: The revised manuscript has responded the comments accordingly. I suggest to accept it at current edition.

Response: Thanks very much for your professional insights and recognition. Your feedback has greatly contributed to the improvement of our manuscript.

Response to comments from the Reviewer # 3:

General comment: The manuscript entitled "Tailoring electrolyte phase separation for high-rate solid-state lithium metal batteries" presents an innovative phase separation

strategy using a single lithium salt to induce controllable phase separation in a single-solvent system. The article provides comprehensive explanations for the mechanism of anion-induced phase separation. Although detailed responses and a careful revision had been conducted, a major revision is still required before acceptance.

Reviewer #1-Comment 1: The phase-separated PVC electrolyte was annealed at 200 ⍰ for 5 hours to evaporate residual monomer. The ionic conductivity of the annealed electrolyte decreases significantly. This result strongly indicates that the residual monomer still plays an important role in ion conduction for the PVC-LiTFSI electrolyte. PVC-LiDFOB electrolyte has more residual monomer than the PVC-LiTFSI, however, it exhibits a relatively low ionic conductivity. The authors state that the PVC-LiDFOB electrolyte shows a single phase that leads to a low conductivity. The reviewer considers that the reason for the low conductivity may lies in the limited dissociation degree of LiDFOB in the electrolyte. Therefore, the contribution of the residual monomer to high ion conduction should be clarified.

Response: Thanks very much for your professional insights. We apologize for the insufficient clarification regarding the contribution of residual monomer to ionic conductivity in our previous manuscript. Corresponding experiments have been supplemented and the relevant discussions have been added to lines 266-271 and 287-292 of the revised manuscript.

It is worth mentioning that the presence of residual monomer is inherently unavoidable during the in-situ polymerization process, which exhibits high intrinsic ionic conductivity and benefits to enhance the overall ionic conductivity of the electrolyte through rational structural design[1]. This constitutes one of the key rationales for developing the phase-separated structure in our work. In our manuscript, the unreacted VC and low-molecular-weight PVC are categorized as oligomers. Although these oligomers exist in a solid state within the electrolyte, they possess solvent-like properties and facilitate rapid ion transport. Previously studies always regulate the electrochemical performance of electrolytes by introducing two immiscible components, which requires the addition of extra plasticizers (e.g., solvents, ionic liquids, or deep eutectic solvents)[2, 3, 4]. While, in our work, the unreacted VC from the polymerization process itself can act as an effective electrolyte additive and efficiently conduct lithium ions and participate in interfacial reactions, making it an ideal component for enhancing ionic conductivity of the electrolyte without introducing foreign species.

In this work, the phase transition behavior during the in-situ polymerization of VC is

regulated simply by the lithium salt, leading to the formation of a phase-separated PVC-based electrolyte (Figure 1a, b). Such an electrolyte features a highly efficient dual-ion conduction pathway (Figure 1f). The oligomer phase forms a continuous amorphous region surrounding the polymer phase, serving as a fast ion-transport channel and contributing substantially to efficient ion conduction (Figure R1a). Meanwhile, ion transport within the polymer phase relies on the segmental motion of polymer chains and the coupled/decoupled hopping of lithium ions (Figure R1b), which exhibits a higher migration energy barrier compared to transport in the oligomer phase and functions as a secondary conduction pathway (Figure 3h).

To clarify the contribution of residual monomers to high ionic conductivity of produced PVC-based electrolytes, we have quantified its content in the electrolyte via ssNMR (Figure 2g) and TGA (Figure R2a) measurements. The results indicate that the residual monomer content in PVC-based electrolytes increases from 13.6% to 18.9% as extending polymerization time from 12 to 48 hours. And the ionic conductivity of the electrolyte declines as decreasing the residual monomer content in the oligomer phase (Figure R2b), accompanied by a notable improvement in the electrochemical stability of the electrolyte (Figure 3c). This demonstrates that the designed phase-separated structure effectively integrates the high ionic conductivity of the oligomer phase with the electrochemical stability of the polymer phase, thereby imparting superior overall performance. Namely, by adjusting the polymerization time, the proportion of the oligomer phase and the degree of phase separation can be effectively tuned to optimize the electrolyte's properties.

It should be pointed out that residual monomers cannot form effective ion conduction pathways in a non-phase-separated polymer electrolyte even if they are uniformly distributed in the polymer matrix, resulting in limited enhancement of ionic conductivity. Namely, the unreacted monomers alone have limited effects on improving ionic conductivity. To validate this point more clearly, a non-phase-separated PVC-LiDFOB electrolyte was synthesized and compared firstly. Despite its significantly lower conversion rate (Figure 2c), the impedance of assembled SS//PVC-LiDFOB//SS cell is significantly higher than that with the phase-separated PVC-LiTFSI electrolyte (Figure 2d). Indeed, as mentioned by the reviewer, the limited dissociation ability of LiDFOB may lead to insufficient ionic conduction, thereby potentially undermining a clear demonstration of the efficacy of phase-separation strategy. In order to address this issue, Lithium bis(fluorosulfonyl)imide (LiFSI) with higher dissociation ability is selected as the lithium salt. As shown in Figure R3a, the PVC electrolyte incorporating with LiFSI does not undergo a phase separation behavior, and the polymerized electrolyte exhibits a

homogeneous bulk structure, demonstrating a distinct structural difference from the electrolyte formulated with LiTFSI (Figure R3b). One can clearly find that, similar to PVC-LiDFOB electrolyte, the PVC-LiFSI electrolyte also exhibits higher impedance and lower ionic conductivity than PVC-LiTFSI under identical testing conditions (Figure R3c). Furthermore, PVC-LiTFSI electrolyte was dissolved in a solvent to disrupt its phase-separated morphology. Subsequently, a defined mass fraction of unreacted monomer was added to the purified PVC electrolyte. The solution was then injected into a separator and heated at a specified temperature for 5 hours under an argon atmosphere to evaporate the solvent. Finally, a non-phase-separated PVC-LiTFSI electrolyte was produced (Figure R3d). The impedance of the non-phase-separated PVC-LiTFSI electrolyte increases significantly (Figure R3e). This is because such a PVC electrolyte prepared by solvent evaporation has high mechanical strength and rigid interface, which leads to the failure of the interface contact between the electrode and the electrolyte, and at the same time the unreacted monomers fail to form a continuous ion-conducting region. In conclusion, the unreacted monomers alone have limited effects on improving ionic conductivity, and inducing phase separation during the in-situ polymerization process is an essential process for enhancing the ion conductivity of PVC-based electrolytes.

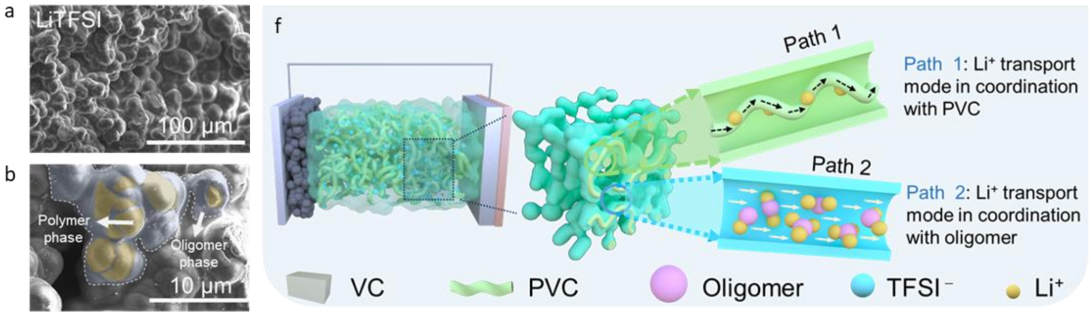

Figure 1. (a,b) SEM images at different magnification of LiTFSI-regulated PVC electrolyte after in-situ polymerization. (f) The schematic diagrams of lithium ion transport mode of LiTFSI-regulated PVC electrolyte.

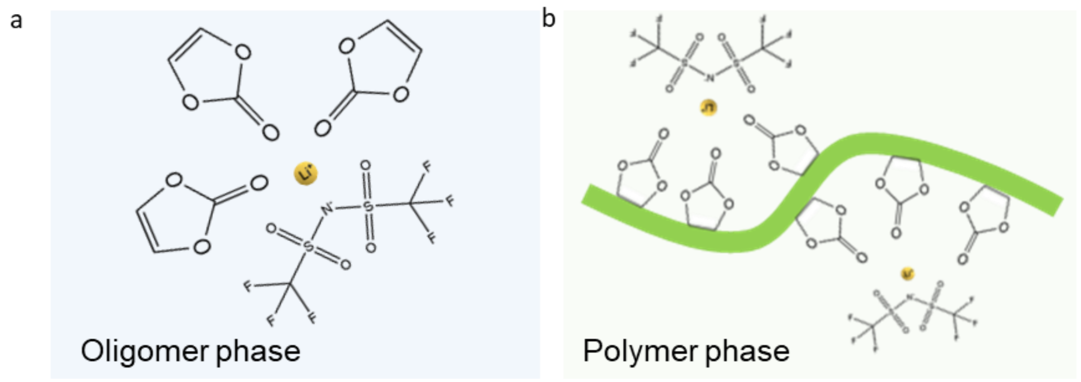

Figure R1. The lithium ion transport modes of LiTFSI-regulated PVC electrolyte. Explanation of solvation structures of (a) oligomer phase and (b) polymer phase in PVC electrolyte.

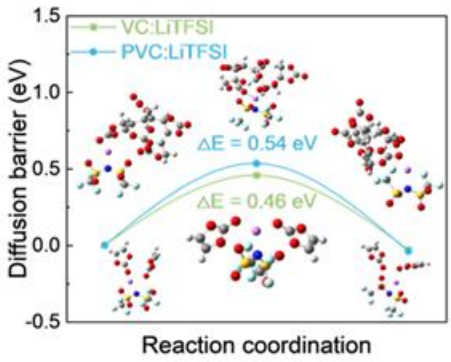

Figure 3. (h)The diffusion barriers of Li between the different coordination structures in polymer phase and oligomer phase.

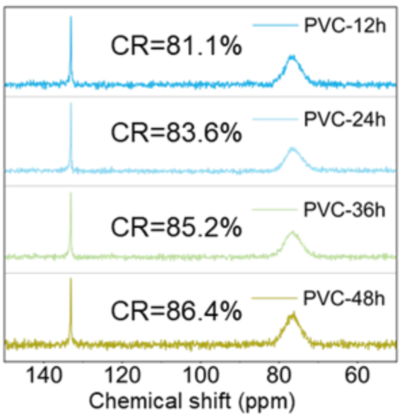

Figure 2. (g) ^{13}C NMR spectra of PVC electrolytes with different polymerization time.

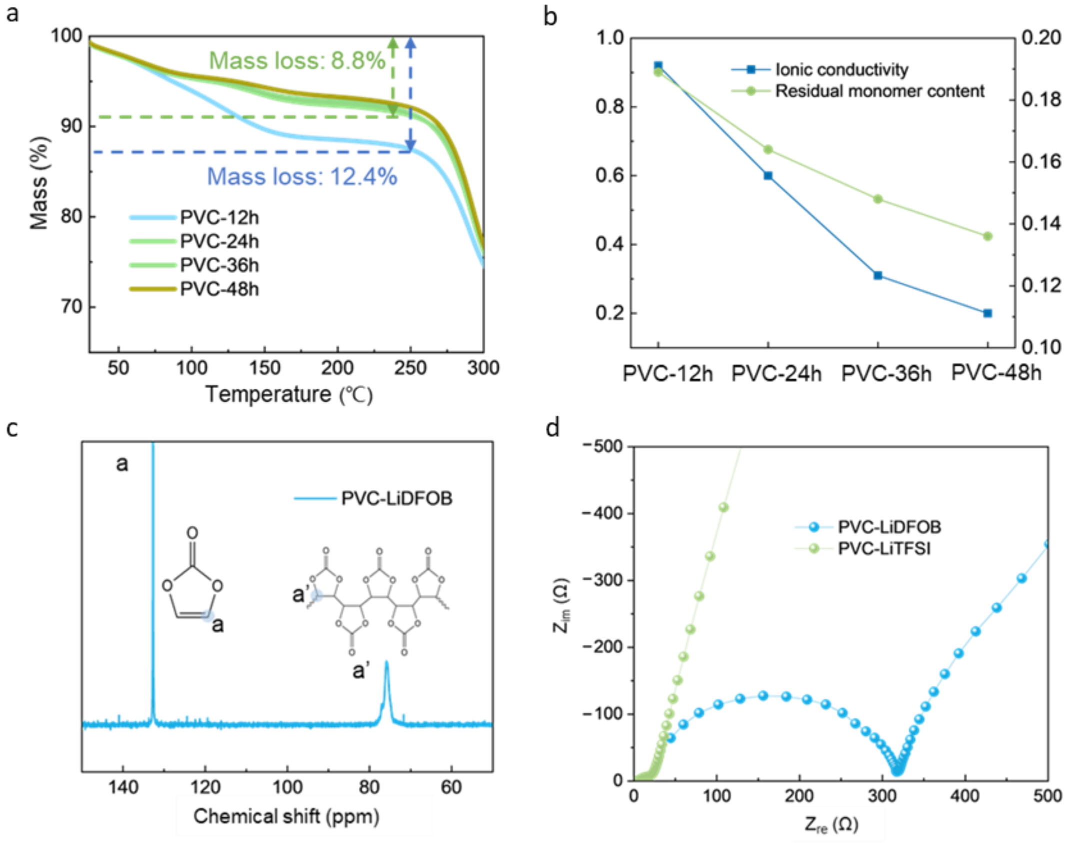

Figure R2. (a) TG curves of various PVC electrolytes with different polymerization time. (b) The ionic conductivity and the contents of residual monomers of PVC electrolytes with different polymerization time. (c) ^{13}C NMR spectrum of LiDFOB-regulated PVC electrolyte. (d) EIS curves of SS//PVC-LiDFOB//SS and SS//PVC-LiTFSI//SS cells at 25 ℃.

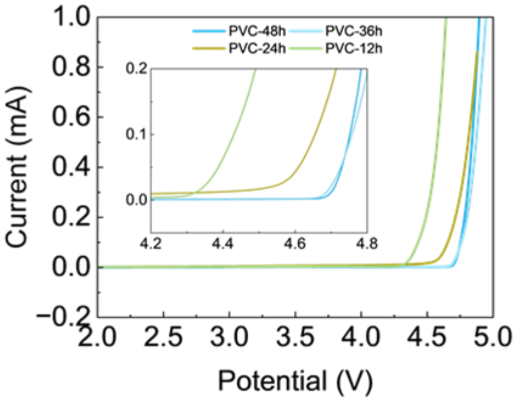

Figure 3. (c) LSV profiles of PVC electrolyte with different polymerization time.

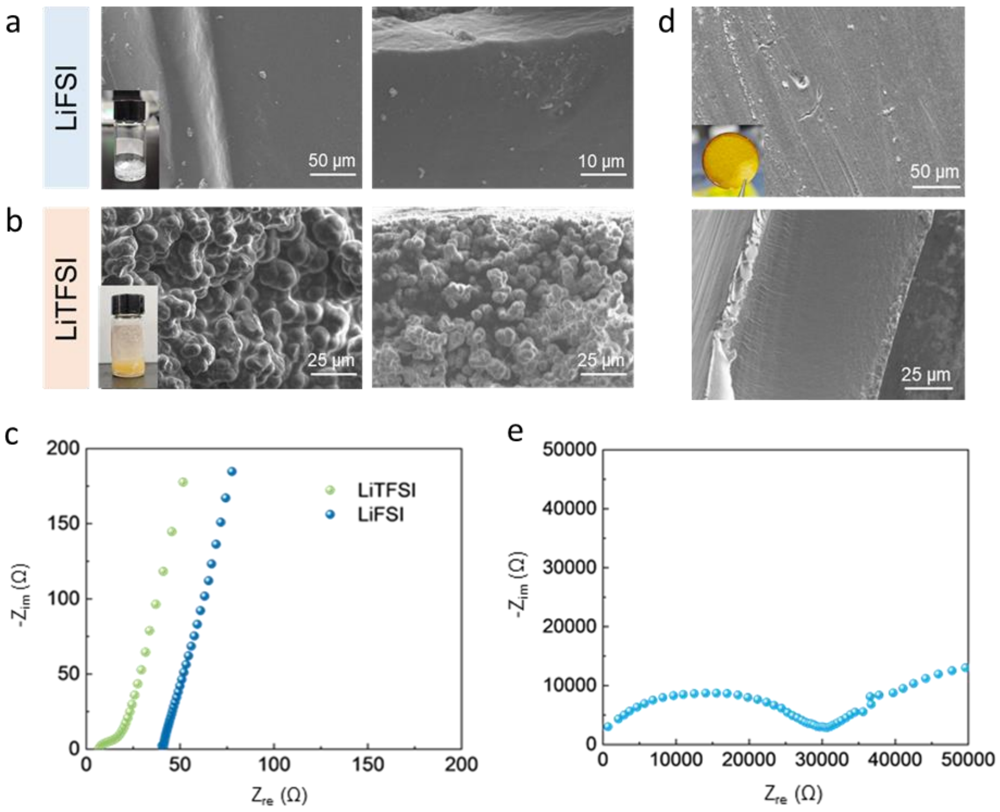

Figure R3. The corresponding optical images and SEM images of PVC electrolyte with (a) LiFSI and (b) LiTFSI. (c) EIS curves of SS //PVC// SS cells with LiFSI and LiTFSI at 25 ℃. (d) The corresponding optical images and SEM images of PVC electrolyte by solvent evaporation. (e) EIS curves of SS //PVC// SS cells with PVC electrolyte by solvent evaporation at 25 ℃.

Response to comments from the Reviewer # 4:

General comment: The authors have addressed my initial concerns, and I believe the manuscript is suitable for publication.

Response: Thanks very much for your professional insights and recognition. Your feedback has greatly contributed to the improvement of our manuscript.

References

1. Li P, *et al.* Efficiencies of Various in situ Polymerizations of Liquid Electrolytes and the Practical Implications for Quasi Solid‐state Batteries. *Angew Chem Int Ed* **62**, e202309613 (2023).

2. Li Z, *et al.* Fluorine–oxygen co-coordination of lithium in fluorinated

polymers for broad temperature quasi-solid-state batteries. *Nat Commun* **16**, 9265 (2025).

3. Xu W, Zhou L, Lu S, He J, Xu Y, Tian L. Fluorine-free gel polymer electrolyte for lithium oxide-rich solid electrolyte interphase and stable Li metal batteries. *Nat Commun* **16**, 9308 (2025).

4. Ye H, Wu B, Sun S, Wu P. A Solid‐Liquid Bicontinuous Fiber with Strain‐Insensitive Ionic Conduction. *Adv Mater* **36**, 2402501 (2024).
